# Novel Two-Step Classifier for Torsades de Pointes Risk Stratification from Direct Features

**DOI:** 10.3389/fphar.2017.00816

**Published:** 2017-11-14

**Authors:** Jaimit Parikh, Viatcheslav Gurev, John J. Rice

**Affiliations:** IBM T. J. Watson Research Center, Yorktown Heights, NY, United States

**Keywords:** Torsades de Pointes, machine-learning, classification and prediction, cardiac modeling, early afterdepolarization

## Abstract

While pre-clinical Torsades de Pointes (TdP) risk classifiers had initially been based on drug-induced block of hERG potassium channels, it is now well established that improved risk prediction can be achieved by considering block of non-hERG ion channels. The current multi-channel TdP classifiers can be categorized into two classes. First, the classifiers that take as input the values of drug-induced block of ion channels (direct features). Second, the classifiers that are built on features extracted from output of the drug-induced multi-channel blockage simulations in the *in-silico* models (derived features). The classifiers built on derived features have thus far not consistently provided increased prediction accuracies, and hence casts doubt on the value of such approaches given the cost of including biophysical detail. Here, we propose a new two-step method for TdP risk classification, referred to as Multi-Channel Blockage at Early After Depolarization (MCB@EAD). In the first step, we classified the compound that produced insufficient hERG block as non-torsadogenic. In the second step, the role of non-hERG channels to modulate TdP risk are considered by constructing classifiers based on direct or derived features at critical hERG block concentrations that generates EADs in the computational cardiac cell models. MCB@EAD provides comparable or superior TdP risk classification of the drugs from the direct features in tests against published methods. TdP risk for the drugs highly correlated to the propensity to generate EADs in the model. However, the derived features of the biophysical models did not improve the predictive capability for TdP risk assessment.

## 1. Introduction

*In-vitro* examination of drug effects on multiple cardiac ion channels and *in-silico* reconstruction of cardiac electrical activity from *in-vitro* experiments are two coupled components in the new paradigm of TdP risk assessment (Sager et al., 2014). At the molecular/ionic level, pharmacological TdP genesis is associated with drug-induced reduction in the net repolarizing current (Antzelevitch, 2007), which is manifested in prolongation of the QT interval in the body-surface ECGs. Drug-induced block of hERG (human Ether-à-go-go-Related Gene) channels, which gate the primary repolarizing current *I*_*Kr*_, is an acknowledged marker for TdP risk prediction. However, recent studies have shown that the classification that is based on the safety margins from the hERG channel assays has moderate concordance with QTc prolongation (Gintant, 2011) and TdP risk (Mirams et al., 2011; Kramer et al., 2013). Drug-induced modulation of non-hERG channels either mitigates (i.e., block of L-type voltage regulated calcium channel current *I*_*CaV*_ and inward late sodium current *I*_*NaL*_) or enhances (i.e., block of slow activating potassium current *I*_*Ks*_ or increase of *I*_*NaL*_) the pro-arrhythmic effects of hERG channel block (Bril et al., 1996; Antzelevitch, 2004; Lacerda et al., 2008; Towart et al., 2009; Fermini et al., 2016). Several multi-channel TdP risk classifiers have already been created (Mirams et al., 2011; Christophe, 2013, 2015; Kramer et al., 2013; Mistry et al., 2015; Okada et al., 2015; Lancaster and Sobie, 2016; Abbasi et al., 2017). Table 1 lists previously published classifiers that are based on several *in-vitro* ion channel assays.

**Table 1:** TdP classifiers based on *in-vitro* ion channel assays.

**Feature**	***In-silico* model**	**Classification**		**References**	***N*_Total_**	***N*_Correct_**
		**TDP+**	**TDP−**			
$\frac{IC_{50,hERG}}{EFTPC}$	NA	Feature < 30	Feature > 30	Redfern et al., 2003	52	NA
*APD*_90_	Ventricular myocyte models of rabbit, rat and human	LDA		Mirams et al., 2011	31	30
$-log\left(\frac{IC_{50,hERG}}{IC_{50,CaV}}\right)$	NA	LR		Kramer et al., 2013	55	50
$\frac{1+a_{0}BP_{CaV}+a_{1}BP_{Na,fast}}{1+a_{2}BP_{hERG}}$	NA	LR		Mistry et al., 2015	31 from (Mirams et al., 2011) 55 from (Kramer et al., 2013)	28
*TDR*	Human ventricular myocyte model	TDR profiles		Christophe, 2015	55 from (Kramer et al., 2013)	NA
$\frac{C_{Drug,Arrhythmia}}{EFTPC}$	3D FEM model of human heart	Feature < 200	Feature > 200	Okada et al., 2015	12	12
EADs	Human ventricular myocyte model	Waveform appearance		Abbasi et al., 2017	12 from (Okada et al., 2015)	11
*APD*_50_ & Diastolic *Ca*^2+^	Human ventricular myocyte models	SVM and PCA		Lancaster and Sobie, 2016	86 from (Kramer et al., 2013) and (Mirams et al., 2011)	75
$\frac{AUCI_{NaL,drug}}{AUCI_{NaL,control}}$ $+\frac{AUCI_{CaV,drug}}{AUCI_{CaV,control}}$	Human ventricular myocyte model	LDA		Li et al., 2017	12	12

*LDA, Linear Determinant Analysis; LR, Logistic Regression; SVM, Support Vector Machine; PCA, Principal Component Analysis; C_drug,EAD_, concentration of the drug that produces EAD; BP_x_, % block of the x (x = Na, fast, CaV, hERG) ion channels; TDR, transmural dispersion of repolarization; C_drug,Arrhythmia_, concentration of the drug that produces arrhythmia in the model; EAD, early after depolarizations; AUCI_x,drug/control_, area under the curve of the x (x = CaV, NaL) current transient at steady state action potential in the presence (drug) or absence of the drug (control). Table also lists the number of compounds analyzed in the study (N_Total_) and the number of correctly classified compounds (N_correct_)*.

The drug-induced changes in the ionic currents result in altering of action potential and calcium transient at the cellular level. These modulations can further trigger events in the cardiac cells, such as early or delayed afterdepolarizations (EADs or DADs), and increase heterogeneity in the electrical activity across the myocardium [i.e., increase in transmural dispersion of repolarization (TDR)]; both effects are thought to be the key determinants for TdP genesis (Wu et al., 2002; Antzelevitch, 2007). *In-silico* reconstruction of drug-induced responses of action potential and calcium transient at cellular or electrical activity at tissue levels could potentially provide better mechanistic insight. The classifiers that use the features from the *in-silico* simulations (derived features) have shown the capability to make good predictions (Table 1) of torsadogenic risk (Mirams et al., 2011, 2014; Christophe, 2013, 2015; Okada et al., 2015; Lancaster and Sobie, 2016; Abbasi et al., 2017; Li et al., 2017). However, in spite of providing better biological insights for TdP genesis, the role of computational models in improving TdP risk prediction is controversial as machine-learning/statistical analysis of the *in-vitro* ion channel measurements (direct features) have been shown to produce equally accurate TdP risk assessment (Kramer et al., 2013; Mistry et al., 2015).

The amount of drug-induced block of the channels depends on the compound's effective free therapeutic plasma concentration (EFTPC). Unfortunately, reported EFTPC values are highly variable (Redfern et al., 2003). The maximum EFTPC values, which is used to determine the ion channel block, also vary across the datasets (e.g., Moxifloxacin 3.5 μM in Crumb et al., 2016, 10.9 μM in Kramer et al., 2013). In addition, the actual free plasma concentrations of drugs in subjects could also differ because of inter-individual variations, impaired metabolism, and interactions with other drugs. In fact, drug concentrations could potentially be much larger than reported maximum EFTPC values. Researchers have employed different strategies to address the uncertainty in EFTPC. Direct and derived features have been evaluated at the drug's EFTPC, at supra-therapeutic drug concentrations (which is several times above maximum EFTPC), or across a wide range of drug concentrations (Christophe, 2013, 2015; Kramer et al., 2013; Mirams et al., 2014; Mistry et al., 2015; Okada et al., 2015; Lancaster and Sobie, 2016; Abbasi et al., 2017; Ando et al., 2017; Li et al., 2017). The range is obtained by titrating up the drug concentrations until a fixed threshold, until a predetermined increase in action potential prolongation is reached, or until EADs are triggered.

Here, we propose a new two-step method for TdP risk classification, referred to as Multi-channel Blockage at Early After Depolarization (MCB@EAD). The MCB@EAD classifier employs as inputs direct or derived features obtained at drug concentrations that produce critical hERG block (~60% block that generates pause-induced EADs in the biophysical models). We test the proposed classifier on several previously published datasets derived from *in-vitro* screening of the ion channels and on a large composite dataset comprising of all datasets. Finally, we examine the connection between TdP risk of the drugs and drug propensity to induce pause-dependent EADs. Our results show that MCB@EAD classification from the direct features performs better or equivalently to previously suggested methods including the classifiers built on derived features from biophysical models. We also highlight the link between the direct and derived feature based classifiers and demonstrate that TdP risk for the drugs highly correlates to the likelihood to produce EADs in the model.

## 2. Methods

Table 2 provides a brief summary for each of the analyzed datasets. More extensive descriptions of the datasets is provided in the Supplemental Material.

**Table 2:** Datasets analyzed for TdP risk. The total number of drugs in each dataset is listed in the “Number compounds” column.

**Datasets**	**Number compounds**	**Risk categorization tested**	**Figure/Table**
Dataset 1	31	TdP+: R1, R2, R3 categories (RCOD)	Figure 3, Table 4
(Mirams et al., 2011)			
Dataset 2	55	TdP+: R1, R2, R3, CM1, CM2, CM3 categories	Figure 3, Table 4
(Kramer et al., 2013)		or label warning (RCOD)	
Dataset 3	12	TdP+: R1, R2, R3 categories (RCOD)	Figure 3, Table 4
(Okada et al., 2015)			
Dataset 4	86	TdP+: CM1 and CH1 categories,	Figure 3, Table 4
(Lancaster and Sobie, 2016)		Drugs in CM2 and CM3 if label warning (RCOD)	
Dataset 5	30	TdP+: R1, R2, R3, CH1, CM1 category	Figures 2A,B, left panel, Figure 4
(Crumb et al., 2016)		or label warning	Tables 4, 5
		TdP+: CM1 and CM2	Figures 2A,B, right panel, Figure 4, Table 5
		TdP+: CM1	Table 5
		TdP+: CM1 and CM3	Table 5
Dataset 6	57	TdP+: CM1	Figure 3, Table 4
(Ando et al., 2017)			
Dataset 7	12	TdP+: CP1 and CP2 category	Figure 3, Table 4
(Li et al., 2017)		High: CP1 Intermediate: CP2 Low: CP3 (RCOD)	Figure 6
Dataset 8	197	TdP+ R1, R2, R3, CH1, CM1 category or label warning	Figure 5
(Combined Datasets 1, 2, 3, 5, 6, and 7)		TdP+: CM1 and CM2	Table 6
		TdP+: CM1	Table 6
		TdP+: CM1 and CM3	Table 6
Dataset 9	26	High: CP1 Intermediate: CP2 Low: CP3 (RCOD)	Figure 6
(Fermini et al., 2016)			
(*IC*_50,*channel*_ extracted from Datasets 1, 2, 3, 5, 6, and 7)			

“*Risk categorization tested” column refers to the TdP risk definition (see more info on definitions in the text) used to test/train the particular datasets, and the “Figure/Table” column list the corresponding Figure and Table numbers where the results of the particular dataset and risk categorization pair are reported. The IC_50_ values and risk category of individual drug for each datasets are also reported in the Supplemental Material*.

### 2.1. Torsadogenicity definition

The definition of drug groups according to their torsadogenic risk is critical for the development of the TdP risk classifiers. Different torsadogenic definitions from previous classification studies are listed below.

Redfern et al. assigned drugs to five categories based on the number of reports of TdP in humans, QT prolongation and TdP associated withdrawal from the market (Redfern et al., 2003). The five categories are:

- R1: Class Ia and III antiarrhythmics with QT prolongation as intended effect.
- R2: Drugs that have been withdrawn from the market due to unacceptable risk of TdP for condition being treated.
- R3: Drugs with numerous case reports of TdP in humans.
- R4: Drugs with isolated reports of TdP in humans.
- R5: Drugs with no published reports of TdP in humans when used alone.

Arizona Center for Education and Research on Therapeutics (AZCERT) maintains a list of drugs associated with QT prolongation/TdP risk (Woosley et al., 2017) (https://crediblemeds.org/) and has categorized the drugs into three groups:

- CM1: Drugs with known risk of TdP. These drugs prolong the QT interval and are clearly associated with a known risk of TdP, even when taken as recommended.
- CM2: Drugs with possible risk of TdP. These drugs can cause QT prolongation but currently there is lack of evidence for a risk of TdP associated with them when taken as recommended.
- CM3: Drugs with conditional risk of TdP. These drugs are associated with TdP but only under certain conditions of their use.

Champeroux et al. assigned drugs into three categories based on the number of reports of TdP cases associated with the drug (Champeroux et al., 2005):

- CH1: Drugs with numerous reports of TdP.
- CH2: Drugs causing QT prolongation and/or TdP at very low frequency.
- CH3: Drugs without reports of TdP or QT prolongation.

Based on a general consensus, a working group formed under the Comprehensive *in vitro* Proarrhythmia Assay (CiPA) initiative picked 28 compounds and categorized them into three groups (Colatsky et al., 2016; Fermini et al., 2016) for testing/training of the classifiers under the new CiPA paradigm:

- CP1: Drugs with high risk.
- CP2: Drugs with intermediate risk.
- CP3: Drugs with low risk.

To consistently compare with other methods we attempt to use the binary TdP definitions [i.e., a drug is either torsadogenic (TdP+) or non-torsadogenic (TdP−)] as in the original publications (see Table 2 and the Supplemental Material for further details regarding the exact risk definition used for the particular datasets). For dataset 7 where tertiary definition is reported, binary TdP definition was defined by placing CP1 and CP2 drugs into torsadogenic (TdP+) and CP3 drugs to non-torsadogenic (TdP−) categories. In the case of the merged dataset or the datasets that lacked binarized TdP definitions (Crumb et al., 2016), we assigned drugs as TdP+ or TdP− using a similar approach as in Lancaster and Sobie (2016). The drugs which fell in the known risk category (CM1) in the CredibleMeds database, R1, R2, and R3 category in Redfern et al. (2003) or drugs with several reports for TdP (CH1) (Champeroux et al., 2005) were assigned to TdP+. For the remaining drugs we categorized the drug as TdP+ if a warning for TdP associated with QT prolongation appeared on its package label (http://dailymed.nlm.nih.gov/). The risk categorization for the drugs are provided in the Supplemental Material. Paroxetine has a warning for TdP in the label and Imipramine was assigned to CH1 category in Champeroux et al. (2005). These two drugs were also assigned as TdP+ in Lancaster and Sobie (2016). Here, we defined them as TdP− as these two compounds are not directly associated with QT prolongation or TdP. These drugs inhibit CYP2D6 and increase plasma concentrations of TdP positive drugs such as Thioridazine (http://dailymed.nlm.nih.gov/). Sometimes alternative definitions were also considered and are explicitly defined in the manuscript.

### 2.2. Drug-induced ion channel block

The *in-vitro* ion channel assay data is converted to drug-induced block of ion channel (direct features) using

(1) $$\begin{array}{l} Block_{channel}=100\times \left(\frac{{C_{Drug}}^{h}}{{IC_{50,channel}}^{h}+{C_{Drug}}^{h}}\right), \end{array}$$

where *IC*_50,*channel*_ is the drug concentrations at which the whole-cell current through particular channels is reduced by half, *C*_*Drug*_ is the concentration of the drug and *h* is the Hill-coefficient. The Hill coefficient values were taken as reported in the original datasets. The *IC*_50_, Hill coefficients, and EFTPC values for each of the datasets are given in the Supplemental Material. Note that Hill coefficient values had little impact, and even fixing Hill coefficient to 1 for all the drugs did not significantly alter the observed classification accuracies (results not shown). The drug-induced blocks of ion channels are used as input features for the machine-learning based classifiers or utilized to scale the maximum conductance (*g*_*channel*_) of the particular ion channels in the *in-silico* models, assuming that drug-induced effect on multiple ion-channels are well represented by a simple conductance-block model (Mirams et al., 2011),

(2) $$\begin{array}{l} g_{channel,drug}=\left(1-Block_{channel}/100\right)\times g_{channel}. \end{array}$$

The effects of drug-induced modulation of multi-channel conductance (Equation 2) on the action potentials (AP) and calcium transients are simulated for all of the compounds using two versions of human ventricular myocyte models (O'Hara et al., 2011; Dutta et al., 2016). Ohara et al. model (OHR) (O'Hara et al., 2011) was picked as it has been chosen as the consensus base model for proarrhythmic risk assessment (Colatsky et al., 2016). OHR model was also shown to have the best predictive capability for TdP risk classification among the few tested models in Lancaster and Sobie (2016). We also utilized the modified version of the OHR model (OHRmv) which has been shown to better fit APD-rate dependence experimental data under drug block conditions, particularly improving the effect of *I*_*NaL*_ block on action potential prolongation (Dutta et al., 2016). Several derived features are extracted from AP and calcium transients, and TdP risk classifiers are constructed using these derived features. The details on the simulation protocols and the computations of the derived features are reported in section 2.6 that describes *in-silico* simulations.

### 2.3. Two-step classifier—multi-channel blockage at hERG EAD(MCB@EAD)

**Figure 1:**
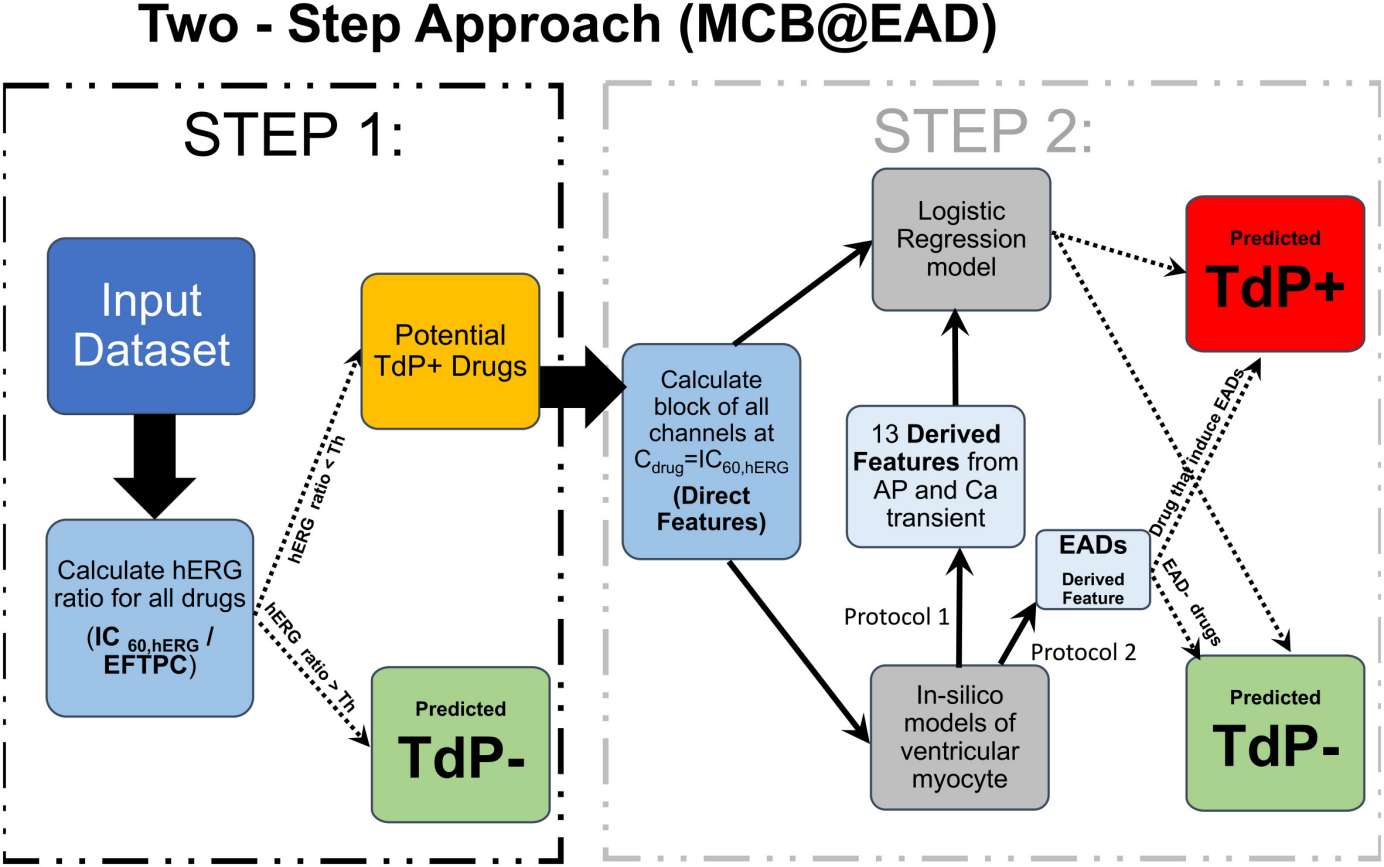
Schematic representation of the MCB@EAD two-step approach. *In-vitro* assay datasets are used to obtain the drug-induced blocks of multiple ion-channels at drug concentrations equal to *IC*_60,*hERG*_. hERG ratio ($\frac{IC_{60,hERG}}{EFTPC}$) is used as the classification criteria in the first step. Drugs that do not result in 60% hERG block at concentrations well above their maximum EFTPC are classified as non-torsadogenic (TdP−). hERG ratio thresholds of 50, 100, 150, and 200 were tested, and the one that provides the best TdP risk discrimination was chosen for the particular datasets. The remaining drugs are considered to be potentially torsadogenic and analyzed in the second step. The drug-induced blocks of multiple ion-channels at 60% hERG block concentrations (direct features) are used as inputs to the logistic regression model for TdP risk classification or used to simulate drug-induced changes in action potential (AP) and calcium (Ca) transients using different protocols. The derived features are extracted from the AP and Ca transients. These derived features are then used to train the logistic-regression model for TdP risk classification or used directly (e.g., in case of EADs) to classify drugs to TdP+ and TdP− categories.

We propose a two-step approach for TdP risk prediction. In the first step, we classified the drugs into non-torsadogenic and potentially torsadogenic categories. We performed the classification in the first step considering solely the block of hERG channels. Using a Redfern-like criteria (Redfern et al., 2003), we obtained the ratio between the drug concentration that produces 60% block of the hERG channel current (*IC*_60,*hERG*_) and the drug EFTPC (i.e., $hERGratio=\frac{IC_{60,hERG}}{EFTPC}$). The motivation for using *IC*_60,*hERG*_ was that the 60% block of hERG channel currents triggers pause-induced EAD in the mid cell type of OHR and OHRmv models (2 Hz pacing rate) at a quiescent interval greater than 700 ms. For example, a drug with hERG *IC*_60_ of 500 nM and EFTPC of 1 nM would yield a threshold of 500. Since this drug's EFTPC would be far from the critical hERG block concentration, we would classify this compound as non-torsadogenic. Previous classifiers based on EAD appearance for different datasets have shown a wide range of thresholds (approximately 30 × −200 × EFTPC) for achieving best TdP risk predictions (Christophe, 2013; Okada et al., 2015). Hence, we tested four different thresholds for hERG ratio of 50, 100, 150, and 200 for all the datasets and chose the one that gives the best classification accuracy. For the remaining drugs with EFTPC above the critical hERG block concentrations (hERG ratio less than the threshold), the role of multi-channel block was examined in the second step using logistic regression classifiers ignoring the EFTPC values of the drugs. The regression classifiers employed as inputs either the *Block*_*channel*_ of additional non-hERG ion channels (direct features) or the features derived from the simulated calcium transient and the AP in the ventricular myocyte models, at drug concentrations equal to *IC*_60,*hERG*_. Such two-step classifier partially solves the problem of EFTPC variability, restricting EFTPC usage only to the first step to primarily discard the drugs that produce insufficient hERG block at extremely high concentrations by classifying them as non-torsadogenic. Therefore, the moderate variations in EFTPC values of the drugs would only matter for a very small population of the drugs with hERG ratio close to the threshold in the classification. A summary of the two-step approach is given in Figure 1.

In order to compare the performance of the classifiers based on the two-step approach to the performance of the classifiers based on features obtained at actual drug EFTPC concentrations, we also constructed TdP risk classifiers using the direct and derived features at reported maximum EFTPC of the drugs. They are referred in the current paper as one-step classifiers (hERG ratio is not utilized for these classifiers).

### 2.4. Classifiers

We utilized statistical/machine-learning models for binary classification of the drugs into TdP+ or TdP− categories. The binarized torsadogenic definitions for each drug were used to train/test the classifier models. Here, we used Logistic regression model. SVM and neural network models were also tested and resulted in comparable classification accuracies (results are shown in the Supplemental Material). Python's scikit-learn package (Pedregosa et al., 2011) (http://scikit-learn.org/stable/) was used to train/test different models. Here, we present results for logistic regression models only as other methods produced similar results. The generalized model equation is described as

(3) $$\begin{array}{l} logit\left(TdP\right)=\frac{1}{1+exp^{-\left(\beta_{0}+\sum_{i}^{n}\beta_{i}Feature_{i}\right)}}, \end{array}$$

where *Feature* represents the input metrics to the model (either direct feature or the derived feature), *n* is the number of input features used to train/test the model, and β_0_ and β*i* (*i* = 1, 2, .., *n*) are the parameters to be determined. The predictive power of the model was evaluated by the leave-one-out (LOO) cross validation technique.

### 2.5. Two-dimensional TdP risk map

A two-dimensional TdP risk map with hERG ratio ($\frac{IC_{60,hERG}}{EFTPC}$) on the x-axis and summation of one or more features (block of *I*_*CaV*_ and *I*_*NaL*_) on the y-axis were constructed for visualization of the two-step (MCB@EAD) classifier. The hERG ratio threshold and regression coefficients from the two-step classifier are used to generate the two-dimensional risk maps. The hERG ratio (step 1 in the two-step classifier) that provides the best classification in the two-step classifier is used to set the threshold along the x-axis. Drugs that fall in the region above the hERG ratio threshold are considered to be non-torsadogenic. For the drugs with hERG ratio less than the threshold, the coefficients of the logistic regression model in step 2 of the two-step classifier are used to determine the threshold along the y-axis of the risk map. An example of separating hyperplane that would be obtained from the second step of the two-step classifier is given by

(4) $$\begin{array}{l} \beta_{I_{CaV}}block_{I_{CaV}}\;+\;\beta_{I_{NaL}}block_{I_{NaL}}\;+\;\overset{n}{\underset{i=3}{\sum }}\beta_{feature,i}\;feature_{i}\\ +\beta_{intercept}\;=\;0 \end{array}$$

where *block*_*I*_*CaV*__ and *block*_*I*_*NaL*__ are the blocks of *I*_*CaV*_ and *I*_*NaL*_, respectively. *feature*_*i*_ are additional input features of the model, such as drug trapping parameters. β_*I*_*CaV*__, β_*I*_*NaL*__, and β_*feature, i*_ represent the regression coefficients. The regression coefficients obtained from the step 2 are normalized to the coefficient β_*I*_*CaV*__ for the *I*_*CaV*_ block. This gives

(5) $$\begin{array}{l} block_{I_{CaV}}+\frac{\beta_{I_{NaL}}}{\beta_{I_{CaV}}}block_{I_{NaL}}+\overset{n}{\underset{i=3}{\sum }}\frac{\beta_{feature,i}feature_{i}}{\beta_{I_{CaV}}}=-\frac{\beta_{intercept}}{\beta_{I_{CaV}}}. \end{array}$$

Thus, the left hand side of Equation (5) is plotted on the y-axis, and the ratio $\frac{\beta_{intercept}}{\beta_{I_{CaV}}}$ determines the threshold along this axis. For example, if only block of *I*_*CaV*_ is taken into consideration, the risk map has the values of *I*_*CaV*_ along the y-axis and hERG ratio along the x-axis.

Ternary classification for Dataset 7 requires multiple hyperplanes with different regression coefficients to separate the high, low and intermediate risk drugs. To represent the ternary classification in a two-dimensional risk map similar to the binary classification, we summed the different features (*Feature*_*sum*_) assuming identical weights for each of the features (i.e., β_1_ = β_2_ = .. = β_*n*_ equal to β_*f*_) reducing the classification model to $\frac{1}{1+exp^{-\left(\beta_{0}+\beta_{f}Feature_{sum}\right)}}$. For Dataset 7, assuming identical weights for different features resulted in similar accuracy to a multinomial logistic regression classifier while providing a simpler visualization in one two-dimensional plot as in the binary classification. The ratio $\frac{-\beta_{0}}{\beta_{f}}$ obtained after training the model is used to set the two thresholds along the y-axis. Along the x-axis arbitrary hERG ratio of 25, the value slightly greater than the maximum hERG ratio observed for high risk drugs in Li et al. (2017) and Fermini et al. (2016), was utilized to separate low and intermediate risk drugs from the high risk drugs. The hERG ratio of 150 obtained from the merged dataset was utilized to separate the high and intermediate risk drugs from the low risk drugs.

### 2.6. *In-Silico* simulations

The alteration in the action potential and calcium transients at the cellular level arising from drug-induced multi-channel block were simulated for all the compounds (Dataset 8) using the OHR model. Using a similar approach as in Lancaster and Sobie (2016), simulations were carried out at three pacing rates (0.5, 1, and 2 Hz) for each of the endo, mid and epi cell types resulting in 9 simulations per drug, and 13 metrics were obtained from the AP and *Ca*^2+^ transients. Simulations were carried out for 1,000 beats to allow the models to reach the steady state. The 13 metrics obtained from the *in-silico* models are listed below:

- Upstroke velocity
- Peak voltage
- Action potential at half maximum duration (APD50)
- Action potential duration at −60 mV (APD @ −60 mV)
- Action potential duration at 90% repolarization (APD90)
- Resting voltage
- Action potential triangulation (AP triangulation), calculated as APD90 - APD30
- Diastolic calcium level (diastolic ${\left[Ca^{2+}\right]}_{i}$)
- Amplitude of the calcium transient (amplitude of CaT)
- Peak value of intracellular calcium (peak ${\left[Ca^{2+}\right]}_{i}$)
- Calcium transient duration at half maximum duration (CaTD50)
- Calcium transient duration at 90% repolarziation (CaTD90)
- Triangulation of the calcium transient (CaT triangulation), calculated as CaTD90 - CaTD30 (calcium transient duration at 30% repolarization)

We systematically construct the classifiers on each of the 13 derived features extracted from the action potentials and calcium transients at two different drug concentrations (i.e., at *C*_*Drug*_ = *EFTPC* and *C*_*Drug*_ = *hERG* *IC*_60_).

**Figure 2:**
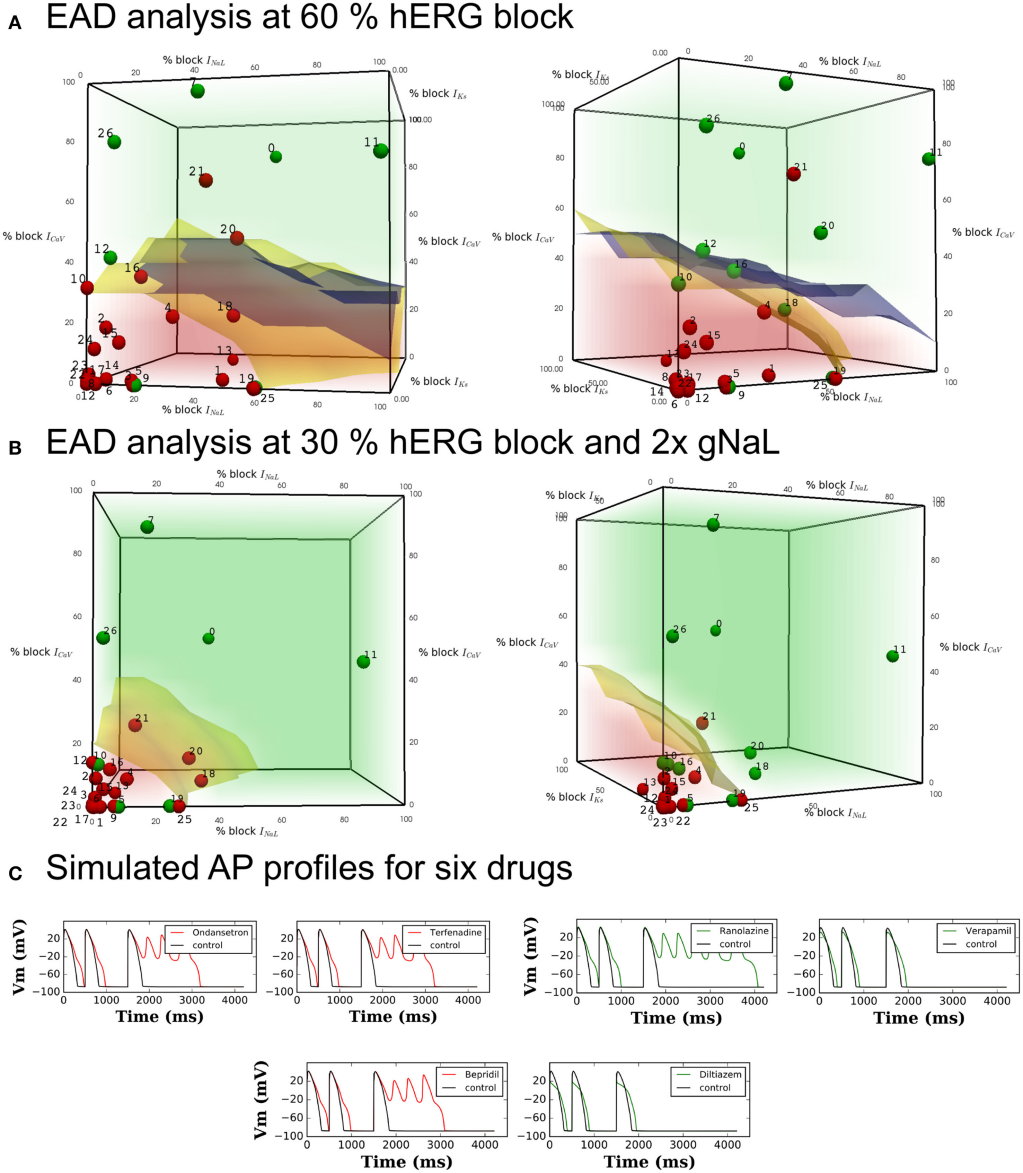
Pause-induced EAD generation: combination of 0–100% *I*_*CaL*_, *I*_*NaL*_, and *I*_*Ks*_ blocks tested at **(A)** 60% hERG block in the OHR and OHRmv **(B)** 30% hERG block in the OHRmv that is modified to simulate LQT3 through increase in late sodium current conductance by 2-fold. Red regions cover the set of combinations of the blocks of the three channels that induced EADs in the model. Green region covers the set of combinations where EADs did not appear. Blue and yellow surfaces represent the separation between the EAD+ and EAD− region in OHR and OHRmv, respectively. The blue and yellow surfaces are plotted together in both plots in **(A)** to allow for easy comparision, but note that the red and green regions corresponds to the results for the OHR model. Block of *I*_*CaL*_, *I*_*NaL*_, and *I*_*Ks*_ at drug concentrations equal to 60 and 30% hERG block are calculated from *in-vitro* patch-clamp data in Crumb et al. (2016) using (Equation 1) and represented as dots in the parameter space of **(A,B)**, respectively. The drugs with torsadogenic potential were plotted as the red dots, while drugs with no known torsadogenicity risk are plotted as the green dots for two different TdP definitions. Definition 1 (left panels) - TdP+: Drugs in R1, R2, R3, CH1, CM1 or torsade label. Definition 2 (right panels) - TdP+: Drugs in CM1 and CM2. The data points for the Dataset 5: 0-Amitriptyline, 1-Azithromycin, 2-Bepridil, 3-Chloroquine, 4-Chlorpromazine, 5-Cibenzoline, 6-Cisapride, 7-Diltiazem, 8-Dofetilide, 9-Flecainide, 10-Lopinavir, 11-Mexiletine, 12-Mibefradil, 13-Moxifloxacin, 14-Nilotinib, 15-Ondansetron, 16-Propafenone, 17-Quinidine, 18-Quinine, 19-Ranolazine, 20-Ritonavir, 21-Saquinavir, 22-Sertindole, 23-Sotalol, 24-Terfenadine, 25-Toremifene, 26-Verapamil. Three drugs (Amiodarone, Lidocaine and Rufinamide) with hERG ratio greater than 200 were not included in the plot. **(C)** Transient AP profiles at 200th beat obtained from simulation of six drugs in the OHR model. Red and green traces show that the drug is defined as TdP+ and TdP−, respectively.

The onset of TdP is usually preceded by a sudden reduction of the heart rate (i.e., by pauses or long cycle lengths) (Neal Kay et al., 1983; Viskin et al., 2000). Here, we test the generation of pause-induced EADs, that are implicated as triggers of TdP (Viswanathan and Rudy, 1999; Liu and Laurita, 2005), in simulations of drug-induced multi-channel blockage in the ventricular myocytes models (O'Hara et al., 2011; Dutta et al., 2016). The basic protocol was similar to that in Viswanathan and Rudy (1999), where stimulation of the cell is carried out 200 times at a constant cycle length of 500 ms. After 200 stimuli, an additional stimulus was applied following a pause of 1,000 ms. Drug-induced EAD development was tested at drug concentrations = *IC*_60,*hERG*_ in the mid cell type. EAD analysis was also performed at *C*_*drug*_ = *IC*_60,*hERG*_ for combinations of *I*_*CaV*_, *I*_*NaL*_, and *I*_*Ks*_ blocks ranging from 0–100% with step of 10% resulting in a set of 1,000 simulations. TdP risk prediction was carried out using ability of drugs to induce EADs as a classification criteria (EAD+: drugs that induce EADs at 60% hERG block concentrations, EAD−: drugs that do not induce EADs at 60% hERG block concentrations). Figure 1 illustrates the classification based on the drug EAD risk. The drugs with hERG ratio greater than the hERG ratio threshold determined for the particular dataset (using the two-step approach on the direct features) were considered EAD−. For the remaining drugs the block of ion-channels was calculated at drug concentration equal to *IC*_60,*hERG*_ and overlaid on the parametric space obtained from EAD analysis at varying combinations of *I*_*CaV*_, *I*_*NaL*_ and *I*_*Ks*_ blocks (Figure 2A), for both the OHR and OHRmv models, to determine whether a drug will induce EAD or not at 60% hERG block concentrations.

The system of ordinary differential equations were solved using the rapid integration scheme (a combination of forward Euler, Rush-Larsen method, Rush and Larsen, 1978 and adaptive time-step) proposed in the original model (O'Hara et al., 2011). For the EAD simulations rapid integration scheme proposed in O'Hara et al. (2011) yielded different results to gold standard simulations with fixed time step of 0.001 ms. Hence, for the EAD simulations we utilized forward Euler method with a time step of 0.001 ms. Execution scripts were written in C++.

## 3. Results

### 3.1. Drugs TdP risk highly correlates to EAD propensity

A short-long cycle length (i.e., pause) often precedes the onset of TdP (Neal Kay et al., 1983; Viskin et al., 2000). The pause is known to facilitate the formations of EADs (Viswanathan and Rudy, 1999; Liu and Laurita, 2005). Here, we test the effects of drug-induced block of different channels on triggering of pause-induced EADs. Block of hERG channel causes prolongation of action potential and can result in the generation of EADs. In the OHR and the OHRmv models, the amount of hERG block required to induce pause-induced EADs is reduced with increase in the duration of the pause. hERG block by 57 and 55% induced EADs in the mid-cell paced at 2 Hz (500 ms pacing cycle length) following a 700 ms pause in the OHR and OHRmv models, respectively. The amount of hERG block required to induce EADs in the OHR and OHRmv model following 1,000 ms pause is reduced to a 47 and 46% block, respectively. Under these critical blocks of hERG channel, modifications of other channels may promote or inhibit the EADs. First, we tested individually the effects of block of six ion-channel currents (*I*_*CaV*_, *I*_*NaL*_, *I*_*Ks*_, *I*_*K*1_, *I*_*Na,fast*_, and *I*_*to*_) in promoting or inhibiting pause-induced EADs at fixed blocks of hERG channels that are marginally above (60%) and below (40%) the critical value (48%) of hERG block that is required to induced EADs in the models. At 60% hERG block, block of *I*_*CaV*_ (> 30% for both the OHR and OHRmv models) and *I*_*NaL*_ (> 60% for the OHRmv model) resulted in suppression of EADs. The remaining five channels had no inhibitory effects. At 40% hERG block, blocks of *I*_*Ks*_ and *I*_*K*1_ led to induction of EADs with lower block of *I*_*Ks*_ currents promoting triggering of EADs compared to *I*_*K*1_. The block of remaining five channels did not result in EADs at 40% hERG block.

For visualization of combined effects of the channels on EAD induction, we performed EAD analysis for varying combinations of block of the three most sensitive non-hERG channels (*I*_*CaV*_, *I*_*NaL*_, and *I*_*Ks*_) regulating EAD generation, each ranging from 0 to 100% at *C*_*drug*_ = *IC*_60,*hERG*_. Figure 2A represents the EAD test for the models. The red region represents the set of combinations of blocks for these currents that resulted in the EADs in the OHR model (EAD+ region). The region in green covers the parameter subspace where no EADs were observed in the OHR model (EAD− region). Separation of the EAD+ region and EAD− region is outlined by the blue and yellow surfaces for the OHR and OHRmv model, respectively (Figure 2A). Among the non-hERG channels, block of *I*_*CaV*_ had the highest modulatory effects on EAD generation under normal conditions. Under critical hERG block (60%), block of *I*_*CaV*_ by more than 30% resulted in suppression of the EADs in both the OHR and OHRmv models (Figure 2A). Block of *I*_*NaL*_ in the absence of the block of *I*_*CaV*_ did not result in suppression of the EADs in the OHR model. Even in the OHRmv model with improved *I*_*NaL*_ formulation, block of more than 60% of the late sodium current was required to suppress pause-induced EADs. Block of *I*_*Ks*_ currents increased the amount of block of *I*_*NaL*_ and *I*_*CaV*_ currents required to prevent EADs. A similar parametric space for EAD generation was also analyzed under enhanced late sodium currents (Figure 2B), associated with LQT3. In the OHRmv model the conductance of the late sodium current was doubled. Block of the hERG channel by 30% was enough to induce EADs in the OHRmv model under enhanced late sodium currents. EAD generation was examined at this 30% hERG block and is shown in Figure 2B. For the simulations with enhanced late sodium currents, the difference in the effects of *I*_*NaL*_ and *I*_*CaV*_ was significantly reduced. At 30% hERG block in the OHRmv model, block of greater than 20 and 30% was required for EAD suppression by *I*_*CaV*_ and *I*_*NaL*_, respectively (Figure 2B).

Table 3 lists the accuracy of TdP risk prediction using EADs as the classification criteria. As an illustration, the data points for the drugs in one of the datasets (Dataset 5) are overlaid on the the parametric space in Figure 2A at hERG blocks of 60% to visualize the agreement between drugs EAD and TdP risks. The drugs with positive TdP risk are shown in red, while the drugs with negative TdP risk are shown in green. Actual AP profiles were also simulated for drugs in Datasets 5 and 7 at 60% hERG block concentrations taking into account the block of all seven channels. Figure 2C gives representative examples of the AP profiles for the multi-channel block of six of the drugs from Dataset 7 simulated in the OHR model. Our results show a good concordance between drugs torsadogenic risk and its propensity to induce EADs (i.e., most of the torsadogenic drugs resulted in pause-induced EADs in the models while no EADs are observed for majority of the non-torsadogenic drugs at 60% hERG block drug concentrations) across all the datasets. Majority of the datasets (Datasets 1, 2, 4, 6) contains values of blocks for two non-hERG channels (*I*_*CaV*_, *I*_*Na,fast*_) of which only *I*_*CaV*_ had an impact on EAD occurrence in the OHR and OHRmv models. Hence, for these datasets, OHR and OHRmv models give identical accuracies as the drugs with greater than 30% *I*_*CaV*_ block would result in EAD suppression in both of these models. For the Datasets 3, 5, and 7, drug-induced blocks of multiple ion channels are reported. However, very few drugs among these datasets are located in the region between the EAD risk decision surfaces for the OHR (blue surface) and OHRmv (yellow surface) models (i.e., the region in the parameter space that would result in a different prediction between the OHR and OHRmv models, Figure 2A). Hence, similar accuracies are observed for both of the models across all datasets. For the Dataset 7, Ranolazine (TdP−) was the only drug that was located on the negative side of the EAD risk decision surface for the OHRmv and to the positive side of the decision surface for the OHR, and hence predicted correctly by the OHRmv model but not by the OHR model. On the contrary for Dataset 5, Quinine (TdP+) ended up on the negative side of the EAD risk separating surface for the OHRmv and to the positive side of the EAD risk decision surface of the OHR model resulting in its incorrect prediction using the OHRmv model (Figure 2A).

**Table 3:** Accuracy of drug classification based on EAD.

**Datasets**	**Accuracy: OHR**	**Accuracy: Modified OHR**
Dataset 1 (Mirams et al., 2011)	97	97
Dataset 2 (Kramer et al., 2013)	87	87
Dataset 3 (Okada et al., 2015)	84	84
Dataset 4 (Lancaster and Sobie, 2016)	87	87
Dataset 5 (Crumb et al., 2016)	83	80
Dataset 6 (Ando et al., 2017)	83	83
Dataset 7 (Li et al., 2017)	92	100
Dataset 8 (Merged Dataset)	83	83

### 3.2. Binary TdP risk discrimination from direct features

Although the biophysical models can provide mechanistic insights underlying TdP genesis, the benefits of using biophysical models in terms of classification is unclear. Here, we wanted to examine the performance of the classifiers built on direct features using the proposed method. The predictive power of the TdP risk classifiers built on the direct features using the proposed method (MCB@EAD) is shown in Table 4 (two-step classifier column). Classification scores on the direct features at EFTPC are reported for comparison (one-step classifier column). The predictive ability of the two-step classifier was comparable or better than those for the classifiers built on the various features in the original datasets and also better than those for the one-step classifier. Most datasets comprise *in-vitro* assay data for drug-induced block of *I*_*Kr*_, *I*_*Na,fast*_, and *I*_*CaV*_ (Datasets 1, 2, 4, and 6). Drug-induced blocks of additional channels were reported in three datasets (Datasets 3, 5, and 7). The classifiers built using the block of *I*_*Kr*_ and *I*_*CaV*_ as inputs provided high TdP risk prediction scores (Table 4). Utilizing the block of *I*_*NaL*_ as an additional input feature improved the prediction for the two datasets (Dataset 3 and Dataset 7) by classifying correctly one more drug, Ranolazine, as TdP−. On the contrary, in Dataset 5, addition of the block of *I*_*NaL*_ to the features reduced the number of correctly classified drugs by one (classifying Ritonavir incorrectly as TdP−). Taking into account the block of additional ion channels (*I*_*Na,fast*_ for Datasets 1, 2, 4, 6 and *I*_*Na,fast*_, *I*_*Ks*_, *I*_*to*_, *I*_*K*1_ for Datasets 3, 5, 7) did not provide any further improvement in the performance of the classifier for all datasets.

**Table 4:** Binary TdP classifier scores of one-step and two-step classification on the direct features for the eight datasets.

**Datasets**			**One step classifier**	**Two step classifier**	**Channel currents**
**Datasets**	***N*_*Drugs*_**	**Original model Accuracy (Feature)**	***C*_*drug*_ = *EFTPC***	***C*_*drug*_ = *IC*_60,*hERG*_**	
Dataset 1 (Mirams et al., 2011)	31	97 (*APD*_90_)	90 (97)	100 (100)	*I*_*Kr*_& *I*_*CaV*_
Dataset 2 (Kramer et al., 2013)	55	91 ($-log\left(\frac{IC_{50,hERG}}{IC_{50,CaV}}\right)$)	89 (82)	95 (95)	*I*_*Kr*_ & *I*_*CaV*_
Dataset 3 (Okada et al., 2015)	12	100 ($\frac{C_{Drug,Arrhythmia}}{EFTPC}$)	58 (66)	92 (92)	*I*_*Kr*_ & *I*_*CaV*_
			66 (66^*^)	100 (92^*^)	*I*_*Kr*_, *I*_*CaV*_ & *I*_*NaL*_
Dataset 4 (Lancaster and Sobie, 2016)	86	87 (*APD*_50_ & Diastolic *Ca*^2+^)	85 (85)	89 (90)	*I*_*Kr*_ & *I*_*CaV*_
Dataset 5 (Crumb et al., 2016)	30		83 (83)	83 (83)	*I*_*Kr*_ & *I*_*CaV*_
			80 (83^*^)	80 (83^*^)	*I*_*Kr*_, *I*_*Cav*_ & *I*_*NaL*_
Dataset 6 (Ando et al., 2017)	36	83 (APD prolongation and EAD (iPSCs))	86 (83)	86 (86)	*I*_*Kr*_ & *I*_*CaV*_
Dataset 7 (Li et al., 2017)	12	100 $\left(\frac{AUCI_{NaL,drug}}{AUCI_{NaL,control}}+\frac{AUCI_{CaV,drug}}{AUCI_{CaV,control}}\right)$	92 (92)	92 (92)	*I*_*Kr*_ & *I*_*CaV*_
			100 (92^*^)	100 (100^*^)	*I*_*Kr*_, *I*_*CaV*_ & *I*_*NaL*_
Dataset 8	197		79 (79)	85 (86)	*I*_*Kr*_ & *I*_*CaV*_

*The total number of drugs in the particular dataset are reported in the N_Drugs_ column. Table also lists the feature used for classification in the original methods and corresponding scores if applicable. The last column shows a list of channel currents used to construct the classifiers. For comparision with the classification accuracy obtained with the direct features, we reported the highest accuracy obtained for the classifiers from the 13 derived features in isolation at different pacing rate/cell type in the bracket in One step and Two step classifier columns*.

* *The derived features were extracted from simulations of the drug-induced block of all the reported ion channel currents in the in-vitro assay datasets*.

**Figure 3:**
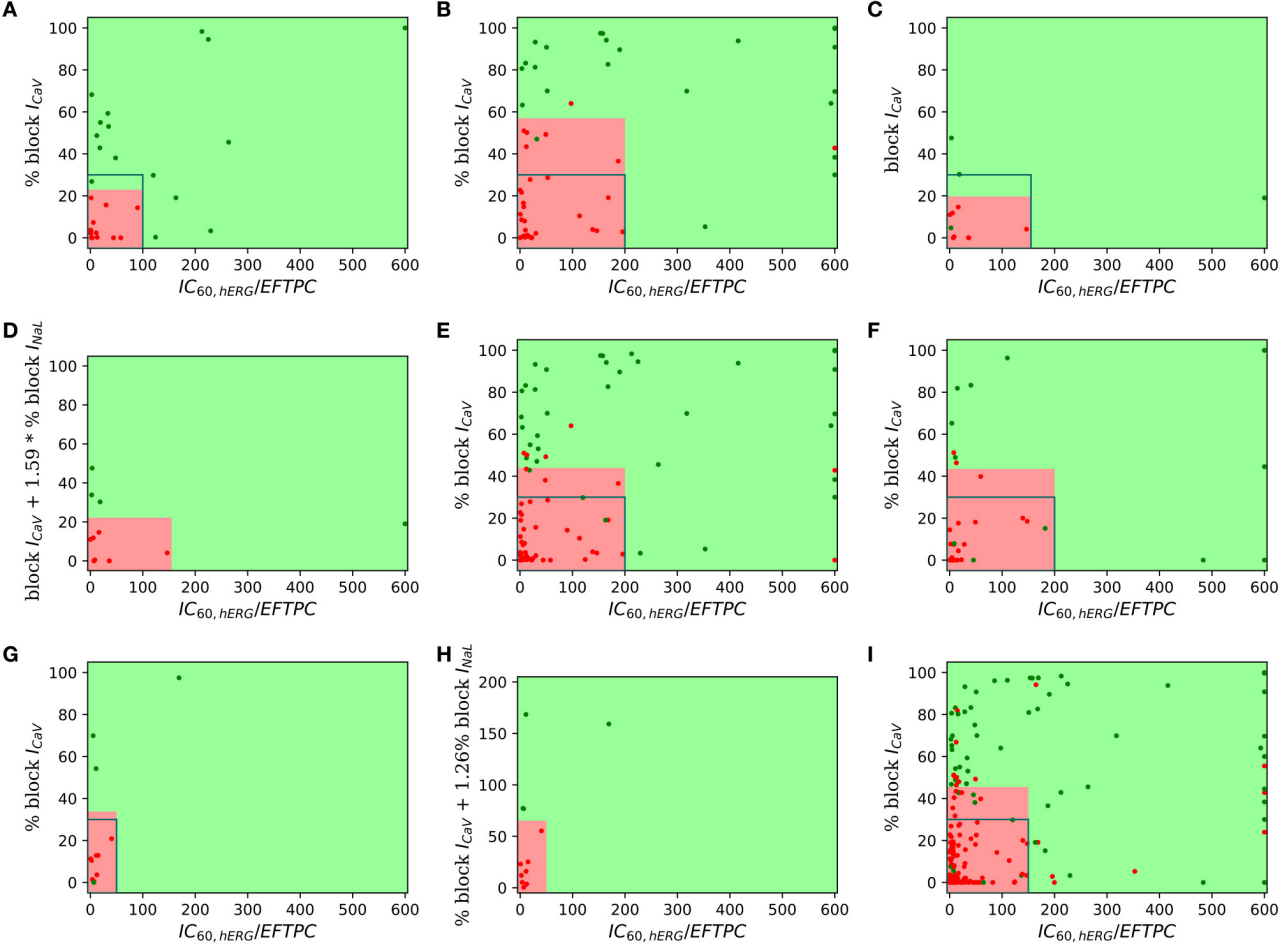
Two-dimensional TdP risk map; blocks of channels are on y-axes and hERG ratio on x-axes for **(A)** Dataset 1 (Mirams et al., 2011), **(B)** Dataset 2 (Kramer et al., 2013), **(C)** Dataset 3 (Okada et al., 2015), **(D)** Dataset 3 (*I*_*Kr*_, *I*_*CaV*_, *I*_*NaL*_) (Okada et al., 2015), **(E)** Dataset 4 (Lancaster and Sobie, 2016), **(F)** Dataset 6 (Ando et al., 2017), **(G)** Dataset 7 (*I*_*Kr*_, *I*_*CaV*_) (Li et al., 2017), **(H)** Dataset 7 (*I*_*Kr*_, *I*_*CaV*_, *I*_*NaL*_) (Li et al., 2017), and **(I)** Dataset 8 (merged dataset). The regions in green are low risk regions and the regions in red are high risk areas. Red dots (•) indicate TdP+ drugs and green dots (•) indicate TdP− drugs. For comparsion purposes, we superimpose a blue rectangular outline that shows the separation between EAD+ and EAD− regions of parameter space. For binary classification the high and intermediate risk drugs in Dataset 7 were assigned TdP+ and the low risk drugs were assigned to TdP−.

**Figure 4:**
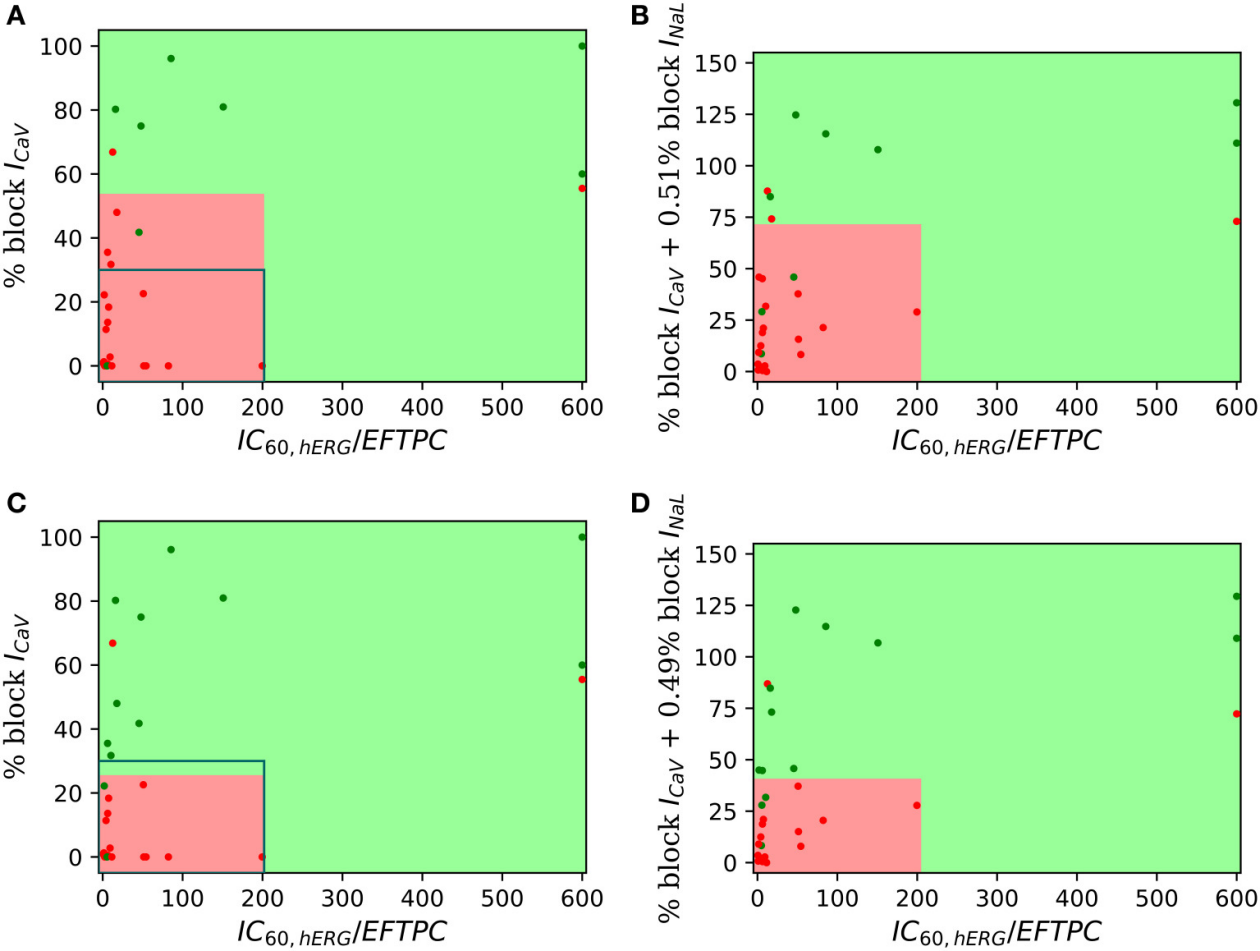
Two-dimensional TdP risk map for Dataset 5 (Crumb et al., 2016). The region in green is a low risk region and the region in red is a high risk area. Red dots (•) indicate TdP+ drugs and green dots (•) indicate TdP− drugs. Blue rectangle outlines the separation of EAD+ (<30% CaV block) and EAD− (> 30% CaV block) regions. The misclassified drugs are **(A)** Ranolazine (CM3, CP3), Cibenzoline (R5), Mibefradil (R4), Saquinavir (CM2) and Amiodarone (CM1, R1); **(B)** Cibenzoline, Ranolazine, Mibefradil, Ritonavir (CM3), Saquinavir and Amiodarone; **(C)** Cibenzoline, Ranolazine, Quinine (CM3), Saquinavir and Amiodarone; and **(D)** Cibenzoline, Ranolazine, Chlorpromazine (CM1), Saquinavir and Amiodarone. **(A,B)** TdP Definition 1: TdP+: Drugs belonging to R1,CM1,CH1 category or drug label has TdP warning. **(C,D)** TdP Definition 2: TdP+: Drugs belonging to CM1 or CM2 category.

For visualization of our two-step approach we presented two-dimensional risk maps. Drug-induced block of the *I*_*CaV*_ or the sum of *I*_*CaV*_ and *I*_*NaL*_ blocks is plotted against the hERG ratio ($\frac{IC_{60,hERG}}{EFPTC_{drug}}$) for each drug in a two-dimensional risk map (Figure 3). The values of the hERG ratio (x-axis) and the block of *I*_*CaV*_ (y-axis) that provide the best discrimination vary widely across the datasets (Figure 3). Using the threshold of 22 and 100 for the block of *I*_*CaV*_ and hERG ratio, respectively, resulted in the perfect classification for Dataset 1 (Mirams et al., 2011). However, for Dataset 2 the hERG ratio threshold of 200 and threshold for *I*_*CaV*_ of 57 provided the best classification. Looking at the risk maps in Figure 3 we can see that the thresholds that provide best classification accuracy vary across datasets resulting in variations in the obtained high risk zones across datasets. For datasets 3, 5, 7 where we also considered block of *I*_*NaL*_ as one of the input feature in addition to the block of *I*_*CaV*_ the value on y-axis of the risk map were reported using Equation (5). A two-dimensional risk map for Dataset 5 for two alternate TdP definitions is shown in Figure 4. On the two-dimensional risk map with only *I*_*CaV*_ block on the y-axis, we also highlight, using a blue rectangular outline, the separation between the region with EAD presence and absence observed in the *in-silico* simulation at varying *I*_*CaV*_ blocks. For example, at critical hERG block simulations in the model at block of *I*_*CaV*_ less than 30% would result in EADs. Block of *I*_*CaV*_ by more than 30% results in suppression of the observed EADs. There is approximate correspondence between the EAD observance region in the *in-silico* model and the high torsadogenic region obtained from classifying the direct-features using machine learning.

### 3.3. Derived vs. direct features as predictors for TdP risk

**Figure 5:**
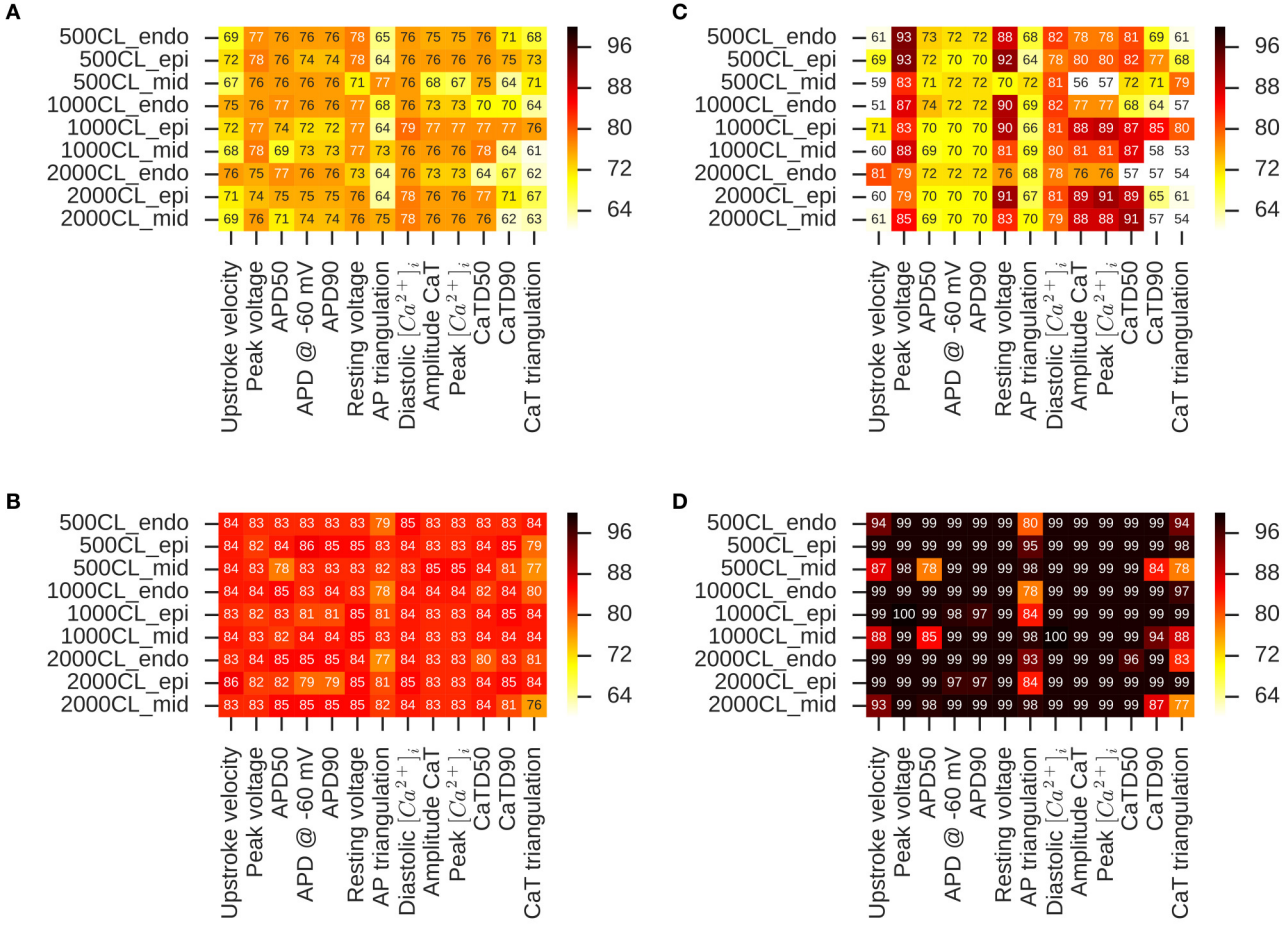
Heat maps of leave-one-out cross validation scores for logistic regression classifiers built on 13 features extracted from the APs and *Ca*^2+^ transients in OHR model simulations. Drug-induced multi-channel block evaluated in the mid, endo and epi cell types at 500, 1,000, and 2,000 ms pacing rates. TdP risk classification at **(A)** EFTPC drug concentrations and **(B)** drug concentrations equal to hERG *IC*_50_. EAD induction classification at **(C)** EFTPC drug concentrations **(D)** drug concentrations equal to hERG *IC*_50_.

We examine the performance of classifiers from various derived features extracted from the simulated action potential and calcium transient in predicting TdP risk. Drug-induced multi-channel block was simulated in the OHR model for all the compounds in the merged dataset (Dataset 8) considering the block *I*_*Kr*_ and *I*_*CaV*_ channels. Simulations were also carried out taking into account drug-induced block of all the channels with available *IC*_50_ values. Taking into account the block of additional channels resulted in similar accuracies and are reported in the Supplemental Material. Figure 5 shows the performance of the logistic-regression classifier to discriminate TdP+ and TdP− drugs (Figures 5A,B) and to predict drug-induced EADs (Figures 5C,D). The classifiers were built on 13 features extracted from the steady-state APs and *Ca*^2+^ transients (for 3 cell types at 3 pacing rates). The derived features were extracted at either EFTPC of the drugs (Figures 5A,C) or at concentrations at which each drug would produce 60% hERG block (*C*_*drug*_ = *IC*_60,*hERG*_) (Figures 5B,D). Among the various derived features obtained at *C*_*drugs*_ = *EFTPC*, diastolic *Ca*^2+^ levels provided the best discrimination score between the TdP+ and TdP− drugs (~79% accuracy at 1 Hz in epi cell type, Figure 5A) in agreement with a previous report (Lancaster and Sobie, 2016). Classifier was also built using *APD*_50_ and Diastolic *Ca*^2+^ together as inputs (the combination that provided the best prediction in Lancaster and Sobie, 2016) but did not give an improved classification for the merged dataset. Several derived features performed well (>90% accuracy) for EAD risk prediction (Figure 5). However, at drug concentrations equal to *IC*_60,*hERG*_ (*C*_*drug*_ = *IC*_60,*hERG*_), each of the 13 features from the *Ca*^2+^ transient and AP provided high classification scores (~85% maximum) for TdP risk assessment (Figure 5C). The highest accuracy for each of the features was obtained at different pacing rates (0.5, 1, and 2 Hz) and for different cell types (endo, mid and epi). The maximum classification scores to discriminate the drugs that induced EAD in the model from the drugs that did not induce EADs was 100% (Figure 5D) for each of the features. Our results suggest that at fixed hERG block concentrations where trigger events such as EADs arise, several derived features obtained from the model including features from the *Ca*^2+^ transient can provide good TdP risk prediction. These derived features also highly correlate with EADs (Figure 5). However, the derived features extracted from simulations of the drug-induced effects in ventricular myocyte model did not result in much improvement in TdP risk assessment over the classifiers built on the direct-features using the proposed method (Table 4). Moreover, combining the direct and derived features to build the TdP risk classifiers also did not improve the classification performance (results are not shown).

### 3.4. Direct features perform well to allow tertiary risk classification

**Figure 6:**
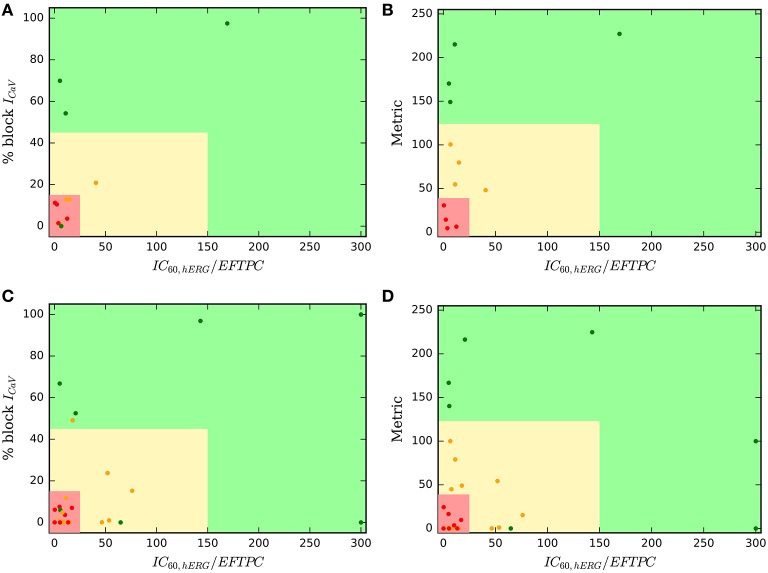
Two-dimensional TdP risk map for Dataset 7 (Li et al., 2017) **(A,B)**, and drugs in Fermini et al. (2016) **(C,D)**. The region in green is a low risk region. Yellow region is an intermediate risk region, and the region in red is a high risk area. Red dots (•) indicate high risk drugs, green dots (•) indicate low risk drugs and orange dots (•) indicate intermediate risk drugs. Metric = % block CaV + % block NaL + degree of drug trapping.

Our results show that classifiers built on the direct features serve as excellent predictors of TdP risk of the drugs categorized into binary risk groups. However, a working committee under the CiPA initiative led by FDA has recently categorized 28 drugs into tertiary risk categories (low, medium, and high risk compounds) (Colatsky et al., 2016; Fermini et al., 2016). Hence, we test the predictive capabilities of the classifier based on direct features to classify the drugs categorized into tertiary risk categories. Figures 6A,B show a two-dimensional risk map for the 12 drugs in Li et al. (2017). These drugs have been analyzed previously using modified OHR model that incorporate dynamic hERG channel interactions. The 12 drugs are a subset of the 28 drugs categorized into three risk categories (CP1, CP2, and CP3) under the CiPA initiative. As a first step, we developed a TdP risk map using only the block of *I*_*CaV*_ channel. The hERG ratio threshold of 150 and the threshold of *I*_*CaV*_ block of 45%, the values that provided best classification for the merged dataset, were utilized for classifying the low high and intermediate risk drugs from low risk drugs Figure 6A. Arbitrary value of hERG ratio and *I*_*CaV*_ block of 25 and 15, respectively (a value greater than the maximum hERG ratio and maximum block of *I*_*CaV*_ among the high risk drugs in Datasets 7 and 9) was utilized to separate high risk drugs from the low and intermediate risk drugs. Three of the four drugs in low risk (CP3) category were classified correctly. Ranolazine was the only misclassified drug. The boundaries of red zone were defined to include all high risk drugs and hence all the drugs from CP1 category were correctly classified. However, several drugs in intermediate risk were incorrectly classified. Next, we built regression classifier using as input metric the sum of block of *I*_*CaV*_, *I*_*NaL*_ channels and the degree of drug-trapping parameter which was shown to be essential to improve risk prediction of intermediate risk drugs in the original dataset. Figure 6B shows the risk map built using this metric. The threshold along the y-axis was obtained from the regression coefficients. The hERG ratio threshold of 25 and 150 were utilized as before. Including the degree of drug trapping characterized by open-bound/closed-bound ratio for the drugs at steady-state (Li et al., 2017) as one of the features in addition to the blocks of *CaV* and *NaL* channels, resulted in the perfect separation of the 12 drugs in 3 categories (Figure 6B).

We employed the same approach to test all of the 28 drugs (Fermini et al., 2016) categorized in CP1, CP2, and CP3 categories under the CiPA initiative. *In-vitro* assays for 12 of these 28 drugs were reported in Crumb et al. (2016) and analyzed in Li et al. (2017). To augment this dataset, we extract the *IC*_50_ values for hERG and *CaV* blocks from Datasets 1, 2, 3, 5, 6, 7 resulting in characterization of 26 of the 28 drugs categorized under the CiPA initiative. Two drugs (Azimilide and Loratidine) were absent in all of the datasets analyzed here and hence were not taken into consideration. We used the mean value of the block if the drug was present in more than one dataset. The final dataset of the 26 drugs is reported in the Supplemental Material. Figure 6C show the two-dimensional risk maps for the 26 drugs with hERG ratio on the x-axis and the block of *CaV* on the y-axis. The 45% *I*_*CaV*_ block threshold and hERG ratio threshold of 150 yielded almost perfect binary classification (high and intermediate vs. low risk drugs) with only 2 (Ranolazine and Tamoxifen) drugs of the 8 from the CP3 category and one drug (Clozapine) of the 16 drugs from CP1 and CP2 cateogry (high and intermediate risk drugs) classifying incorrectly. However, no clear separation was observed amoung the drugs in CP1 and CP2 categories, with several drugs from CP2 category ending up in the high risk region. Considering additional features such as *I*_*NaL*_ and the degree of drug trapping (if either of the features were not available their value was set to zero) and utilizing the threshold obtained from training the Dataset 7 (Figure 6B) resulted in only 3 intermediate risk drugs (Clarithromycin, Domperidone, Droperidol) and 1 low risk drug (Tamoxifen) of the 26 drugs to be misclassified (Figure 6D). The classifier already performs well in spite of testing data from heterogeneous sources with some missing values. Further refinement of the method may be possible when a dataset is available with all 28 drugs characterized with a consistent methodology.

### 3.5. Diverse definition of drugs torsadogenicity lead to different prediction accuracies

Different binary definitions for the drug's torsadogenic risk have been used across the literature (Lancaster and Sobie, 2016; Ando et al., 2017; Wiśniowska and Polak, 2017). Tables 5, 6 list the different classification accuracy scores obtained for four different binary TdP definitions (Datasets 5 and 8, respectively). Classifiers were constructed on the block of one or multiple-ion channels as inputs at critical hERG block concentrations (*C*_*drug*_ = *IC*_60,*hERG*_). The various definitions not only resulted in variability of classification scores (Tables 5, 6), but also changed the role of different ion channels in accurate TdP risk classification (Table 5). Using the block of *I*_*CaV*_ currents provided the best accuracy scores under two of the four definitions (Target 1 and Target 2) for Dataset 5. Including the effects on additional ion-channels did not improve classification scores for these two definitions. Taking into account the block of late sodium currents in addition to *CaV* channels, provided the best classification accuracy for remaining two of the four TdP definitions (Target 3 and 4) (Table 5).

**Table 5:** Accuracy scores of TdP classifiers on the direct features for Dataset 5 under four different TdP definitions.

**Crumb et al., 2016**	**Target 1**	**Target 2**	**Target 3**	**Target 4**
	**2-step** *C*_*drug*_ = *IC*_60,*hERG*_**, direct features**			
Step1:*IC*_60,*hERG*_/*EFTPC*	73	60	60	60
Step1:*IC*_60,*hERG*_/*EFTPC*	83	83	70	70
Step2:% block *I*_*CaV*_				
Step1:*IC*_60,*hERG*_/*EFTPC*	80	83	83	76
Step2:% block *I*_*CaV*_ & *I*_*NaL*_				
Step1:*IC*_60,*hERG*_/*EFTPC*	80	83	83	76
Step2:% block *I*_*CaV*_, *I*_*NaL*_ & *I*_*Ks*_				
	**2-step** *C*_*drug*_ = *IC*_60,*hERG*_**, Derived Features**			
Step1:*IC*_60,*hERG*_/*EFTPC*	83	83	83	76
Step2: Derived features				

*Target1: TdP+ = CM1, CH1, R1, R2, R3, and FDA label QT prolongation and torsade warnings. Target 2: TdP+ = CM1 and CM2. Target 3: TdP+ = CM1. Target 4: TdP+ = CM1 and CM3. Row reporting the maximum accuracies obtained using the derived features is reported for comparision with the direct features*.

**Table 6:** Accuracy scores of TdP classifiers on the direct features for Dataset 8 under four different TdP definitions.

**Dataset 8**	**Target 1**	**Target 2**	**Target 3**	**Target 4**
	**2-step** *C*_*drug*_ = *IC*_60,*hERG*_**, direct features**			
Step1:*IC*_60,*hERG*_/*EFTPC*	77	69	64	66
Step1:*IC*_60,*hERG*_/*EFTPC*	85	77	74	73
Step2:% block *I*_*CaV*_				
Step1:*IC*_60,*hERG*_/*EFTPC*	85	76	74	72
Step2:% block *I*_*CaV*_ & *I*_*Na,fast*_				
	**2-step** *C*_*drug*_ = *IC*_60,*hERG*_**, derived features**			
Step1:*IC*_60,*hERG*_/*EFTPC*	86	76	74	73
Step2: Derived features				

*Target1: TdP+ = CM1, CH1, R1, R2, R3, and FDA label QT prolongation and torsade warnings. Target 2: TdP+ = CM1 and CM2. Target 3: TdP+ = CM1. Target 4: TdP+ = CM1 and CM3. Row reporting the maximum accuracies obtained using the derived features is reported for comparision with the direct features*.

## 4. Discussion

Evaluation of drug-induced alterations in multiple cardiac ion-channel currents to determine the drug's torsadogenic potential is currently under investigation through initiatives like CiPA (Comprehensive *In-vitro* Proarrhythmia Assay) (Sager et al., 2014; Fermini et al., 2016). We have developed a novel two-step method (MCB@EAD) for classification of drugs according to their torsadogenic risk. Using the proposed method, we examined the drug effects at fixed hERG block (i.e., 60% block) concentrations for all tested compounds. This approach allows to isolate the effects of hERG and non-hERG channels in the classification problem. The proximity of the drug's EFTPC to the concentration that results in the critical hERG block provides one of the metrics for determining the drug's TdP risk. For the drugs that induce this critical hERG block at concentrations below a set threshold, the drug-induced effects on non-hERG channels provide an additional metric to determine pro-arrhythmic risk independently of the drug's EFTPC. Our classifier shows improved or equivalent prediction to existing methods. However, one of the advantages compared to previous studies is that the direct and derived features based MCB@EAD classifiers were tested on several *in-vitro* assay datasets reported previously, as well as on a large composite dataset obtained by merging the different datasets together. One of the important findings of the study was that MCB@EAD TdP classifiers from the direct features provides excellent TdP risk prediction and performs identical to the TdP classifiers from the derived features, which are extracted from complex biophysical models. Although the derived features provided by the biophysical models did not improve the predictive capability for TdP risk assessment, the biophysical models helped determine the amount of block that generates EADs (i.e., the concentration at which the direct features are analyzed using the MCB@EAD classifier). The proposed method not only performs comparably or better than the previous classifiers (Table 4) across various *in-vitro* assay datasets published previously, but also highlights the link between direct and derived feature based classifiers. The results also show strong correlation between the drugs that generate EADs and the drugs with positive TdP risk.

### 4.1. Ion-Channels critical for TdP risk prediction

Although the role of multiple ion-channels have been suggested for improved TdP risk prediction, classifiers have been primarily built on the blocks of *I*_*Kr*_, *I*_*CaV*_, and *I*_*Na,peak*_ currents (Mirams et al., 2011; Christophe, 2013, 2015; Kramer et al., 2013; Lancaster and Sobie, 2016). A recent assay reports the drug-induced effects on seven ion channels (Crumb et al., 2016) providing an opportunity to identify the ion channels that are important for pro-arrhythmic risk assessment. The results of our parametric simulations of EAD indicate a potential role of block of *I*_*CaV*_, *I*_*NaL*_, and *I*_*Ks*_ currents, in addition to *I*_*Kr*_, for determination of torsadogenic risk of the drugs (Figure 2). It should be noted that the EAD simulations results are highly dependent on the ventricular myocyte model. For example, block of late sodium current in the OHRmv plays a more prominent role in regulation of AP sensitivity to EADs as compared to the OHR model (Figure 2A). The *I*_*Ks*_ plays a much bigger role in Ten Tusscher and Panfilov model (Ten Tusscher and Panfilov, 2006) than OHR model in regulation of APD as shown in Mirams et al. (2014). The present datasets have limited examples of block of *I*_*CaV*_, *I*_*NaL*_, and *I*_*Ks*_ currents in the same compounds. Moreover, among the non-hERG channels, the regulatory effect of *I*_*CaV*_ block was the highest with the block of *I*_*CaV*_ by only 30% resulting in EAD suppression at critical hERG current block (Figure 2). The classifiers constructed on the block of *I*_*Kr*_ and *I*_*CaL*_ provided the best discrimination between torsadogenic and non-torsadogenic drugs for the majority of the datasets tested here, including the dataset where drug-induced effects on seven ion channels were reported (Figure 2, Tables 4, 5). Our results suggest that among different channels, examination of block of *I*_*CaV*_ and *I*_*Kr*_ might be the most critical for TdP risk prediction. Relative block of *I*_*CaV*_ and *I*_*Kr*_ (among the three currents measured in the *in-vitro* assay) was shown to provide the best risk prediction, with no role of peak/fast sodium currents in improving the classification in Kramer et al. (2013).

Examination of late sodium block can be important for the drugs with low to moderate *I*_*CaV*_ block as these drugs would be predicted TdP+ if only *I*_*Kr*_ and *I*_*CaV*_ block are considered for risk prediction. Moderate to high block of *I*_*NaL*_ by these drugs can result in suppression of EADs (Figure 2) indicating lower TdP risk. Earlier datasets did not report values for *I*_*NaL*_ block. Dataset 5 reports the value of drug-induced block of seven ion-channels, including the block of *I*_*NaL*_. Among the drugs with low to moderate *I*_*CaV*_ in Dataset 5, only three drugs [Ranolazine(CM3), Toremifene(CM2) and Quinine(CM3)] have greater than 30% *I*_*NaL*_ block at critical hERG block concentrations. For the limited data with inconsistent risk categorization, taking into account the *I*_*NaL*_ block did not improve predictive power of the classifiers (Tables 4, 5). A small improvement in TdP prediction was observed for Datasets 3 and 7 (Okada et al., 2015; Li et al., 2017) when considering drug-induced block of *I*_*NaL*_ as one of the input features by correctly classifying Ranolazine (the only drug with high late sodium block in absence of *I*_*CaV*_ block) in both the datasets (Table 4 and Figure 3). The limited data and inconclusive/minor improvement in torsadogenic risk classification make it difficult to ascertain the role of ion channels such as *I*_*NaL*_ and *I*_*Ks*_ in predicting TdP risk.

### 4.2. Predictive power of direct vs. derived features

*In-silico* biophysical models can be thought of as a complex non-linear transfer function, which translates the drug-induced multi-channel block effects at channel level (input) to alterations in APs and calcium transients at cellular/tissue levels (output). Several *in-silico* electrical biophysical models of human ventricular cell models have been published over the last decade (e.g., Ten Tusscher and Panfilov, 2006; Grandi et al., 2010; O'Hara et al., 2011; Himeno et al., 2015). TdP risk classification on features extracted from the drug-induced responses in isolated cell (Mirams et al., 2011; Christophe, 2013, 2015; Lancaster and Sobie, 2016), tissue (Trenor et al., 2013; Kubo et al., 2017) or organ level (Okada et al., 2015) computational models can provide physiological/mechanistic insights. Moreover, *in-silico* models serve as an excellent tool for evaluation of drug-safety in diseased conditions (Trenor et al., 2013; Kubo et al., 2017). Our simulations in the OHRmv model under pathological conditions (enhanced late sodium currents) reveal that EAD can appear at significantly lower drug concentrations as only 30% hERG block was required to induce pause-induced EADs under pathological conditions compared to 60% hERG block under normal conditions. Moreover, the modulatory effects of non-hERG channels on EAD induction was also significantly different for simulations under pathological conditions (Figure 2). On the other hand, biophysical models show considerable differences in their formulations and can lead to different predictions based on the chosen model. Simulations of EAD generation show a significant difference between the surfaces separating EAD+ region from EAD− region, that are obtained from the OHR and OHRmv models (Figure 2). Recently, several efforts have been carried out for optimization of *in-silico* cardiac cell models for pro-arrhythmia risk assessment (Dutta et al., 2016; Mann et al., 2016; Li et al., 2017).

Statistical/machine learning classifiers that use measured *in-vitro* block of multiple cardiac channels (direct features) as their input (Kramer et al., 2013; Mistry et al., 2015) demonstrated comparable accuracy as compared to TdP risk classifiers built on derived features, questioning the need of additional complexity provided by the *in-silico* models. On the contrary, the study by Okada et al. suggested the need of highly detailed three-dimensional cardiac models for pro-arrhythmic risk assessment and showed relatively low predictive ability using the direct features and also certain derived features from the *in-silico* lumped parameter (zero-dimensional) cellular models (Okada et al., 2015). Derived features from *in-silico* simulations that incorporate dynamic drug-hERG channel interactions were shown to improve prediction of TdP risk (Li et al., 2017). For all the datasets tested here, including the datasets in Okada et al. (2015) and Li et al. (2017), we showed that the classifiers built on the direct features performed equally or better than the previously developed classifiers on the derived features (Table 4). Our results show that for currently available *in-vitro* assay datasets simple models based on the direct features can provide similar accuarcy to more complex models based on derived features. It should be noted that our two-dimensional risk classifiers on the direct features also utilized insights gained from the computational models (the direct features are examined at critical hERG block concentrations where EAD can arise in the *in-silico* models). Our parametric simulation for EAD induction highlights one of the possible reasons for the insignificant improvement in predictive power of classifiers built on the derived features from the *in-silico* models. Although a non-linear surface is obtained from the *in-silico* models separating the EAD+ and EAD− regions (Figure 2), a hyperplane

(6) $$\begin{array}{l} a\times block_{I_{CaV}}+b\times block_{I_{NaL}}+c\times block_{I_{Ks}}+d=0 \end{array}$$

constructed using direct features can result in nearly identical separation, where *a*, *b*, *c*, and *d* are the parameters of the hyperplane and *block*_*I*_*CaV*__, *block*_*I*_*NaL*__, and *block*_*I*_*Ks*__ are the values of block of *I*_*CaV*_ *I*_*NaL*_, and *I*_*Ks*_, respectively. Moreover, with most of the datasets comprising values for block of few channels (Mirams et al., 2011; Kramer et al., 2013) and the much higher incidence of drug-induced block of particular ion channels (*I*_*NaL*_, *I*_*CaV*_, and *I*_*Kr*_) even when drug-induced modulation of several channels are examined (Crumb et al., 2016), the result is a congregation of majority of the data in a small region of the plausible high-dimensional risk space (e.g., see Figure 2). For example, the data in Mirams et al. (2011) and Kramer et al. (2013) would fall on a single edge of the 3D EAD space in Figure 2 in the absence of values for drug-induced block on *I*_*NaL*_ and *I*_*Ks*_ in these datasets. This allows risk classification to be performed by a hyperplane with a single parameter, such as *block*_*I*_*CaV*__. Here, we utilized an additional metric, i.e., the hERG ratio, to further improve the classification performance of the direct-feature based classifiers (Figures 3, 4, 6). For the limited data currently available, risk classification using simple statistical models built on the direct features as the one presented here may suffice.

### 4.3. Diversity in the proposed derived features

The classifiers built on derived features obtained from the *in-silico* models are based on certain underlying physiological phenomenon (APD, increase in calcium levels, etc.). Hence, derived features are thought to allow better extrapolation to examine drug targets other than those in the training set. However, diverse derived features from the *in-silico* models have been suggested as possible candidate metrics. Several features from the biophysical models, such as *APD*_50_, *APD*_90_, calcium level peak, and *CaD*_90_ provided the best classification depending on the selected *in-silico* model (Mirams et al., 2011). Other derived features (EADs, TDR, change in *I*_*CaV*_ & *I*_*NaL*_) extracted from the AP and calcium transient (Christophe, 2013, 2015; Li et al., 2017) have also been suggested as possible candidate metrics for TdP risk prediction. Rather than examining the individual features separately, a recent study performed a comprehensive feature selection among 331 metrics and determined that two metrics, *APD*_50_ and diastolic *Ca*^2+^ in the OHR model at 1 Hz pacing, provided the best discrimination between torsadogenic and non-torsadogenic drugs (Lancaster and Sobie, 2016). The overall diversity in reported plausible candidate metrics for TdP risk classification can be attributed to different simulation protocols, drug concentrations and biophysical models. We showed that several derived features obtained from the *in-silico* models may track together and provide equal predictive power for risk classification when examined independent of drug EFTPC (Figure 5C). Equal predictive ability of several features makes it difficult to determine the underlying causal mechanism. In addition, the identical performance of several derived features limits the extensibility of the classifier to untrained targets, as classification results depend on the specific set of features chosen to perform the classification. For example, examination of untrained ion-channel targets using a classifier with diastolic *Ca*^2+^ level as the primary risk discriminating feature predicts a decrease in torsadogenic risk for increased *Na*^+^ − *Ca*^2+^ currents (Lancaster and Sobie, 2016). On the contrary, TdP risk prediction under *Na*^+^ − *Ca*^2+^ modulation using a classifier with *APD*_50_ or *APD*_90_ as the primary discriminating feature would predict opposite effects, with decreased *Na*^+^ − *Ca*^2+^ exchanger current being associated with decreased TdP risk. Moreover, the derived features obtained from the highly complex biophysical models did not result in improved prediction over the classfiers built on the direct features using the proposed method.

### 4.4. Limitations

One of the primary limitations is the quality of the datasets itself. The variability in the *IC*_50_ values among the several datasets can be one of the reasons for the observation of different thresholds for the hERG ratio and *I*_*CaV*_ block that resulted in the best discrimination between TdP+ and TdP− drugs (Figure 3). Quantification of the uncertainties in the *in-vitro* channel screening data and their effects on risk prediction are presented in Johnstone et al. (2016). Inconsistencies in risk definition presents another important challenge for torsadogenic risk assessment. Wiśniowska and Polak (2017) reports a comprehensive list of compounds that have been inconsistently defined as TdP+ or TdP− in different studies to develop torsadogenic risk classifiers. The different categorizations can lead to different interpretations and accuracy scores for TdP risk determination (Table 5). Standardization of torsadogenicity definition, which would allow comparison of the performance of different classifiers/features, is required. Certain steps in this direction have been started. Based on a general consensus, a working group formed under CiPA initiative picked 28 compounds and categorized each into three groups (Colatsky et al., 2016; Fermini et al., 2016) for testing/training of the classifiers. *In-silico* simulations of dynamic drug-channel interactions might be essential to further improve the TdP risk assessment (Li et al., 2017). Inclusion of the drug-binding parameter, in addition to the amount of block of ion-channels, resulted in 100% prediction using our approach. Sufficient *I*_*Kr*_ block was assumed to be necessary for TdP generation in our method. The effects of non-hERG channels are thought to enhance or mitigate the torsadogenic effects of *I*_*Kr*_ block. The method resulted in excellent predictive performance across several datasets that report drug-induced block of various ion channels only. However, drug-induced enhancement of ion-channel currents such as *I*_*NaL*_ can result in increased TdP risk in the absence of hERG block (Lacerda et al., 2008; Yang et al., 2014). The method could be further extended to examine such effects when more data are available. The present work not only provides a new method for *in-vitro* ion-channel screening based TdP risk classification but also highlights several important issues in regards to the use of drug-induced multi-channel blockage for torsadogenic risk prediction.

## Author contributions

JP, VG, and JR wrote the manuscript. JP, VG, and JR designed the research. JP and VG performed the simulations. JP, VG, and JR analyzed the data.

### Conflict of interest statement

All authors are employees of IBM Research. The authors declare that the research was conducted in the absence of any commercial or financial relationships that could be construed as a potential conflict of interest. The reviewer EP and handling Editor declared their shared affiliation.
