# Supplementary material for: Novel Two-Step Classifier for Torsades de Pointes Risk Stratification from Direct Features
Source: Front Pharmacol. 2017 Nov 14;8:816. doi: 10.3389/fphar.2017.00816 (PMC5694470; doi:10.3389/fphar.2017.00816)
Supplement: Supplementary file 1 [file DataSheet1.pdf]

# *Supplementary Material*: Novel two-step classifier for Torsdes de Pointes risk stratification from direct features

**Jaimit Parikh [1], Viatcheslav Gurev [1] and John Jeremy Rice [1*]**

Correspondence*:
John Jeremy Rice
johnrice@us.ibm.com

# 1 SUPPLEMENTARY DATA

## 1.1 Datasets

**Dataset 1: Mirams et al. 2011** (Mirams et al., 2011). The study analyzed 31 drugs for torsadogenic risk. $IC_{50}$ values for drug-induced blocks of $I_{Kr}$, $I_{Na,fast}$ and $I_{CaV}$ were considered. The TdP risk of the compound was defined based on the risk classification proposed in Redfern et al. (Redfern et al., 2003) with modifications in the risk category based on the updated literature. For binarized TdP definition, drugs in R1, R2 and R3 categories were considered TdP+ and the ones in R4 and R5 categories as TdP-. Integrated effect of multi-channel block were simulated using *in-silico* models of cardiac cells. The classifier was built on $APD_{90}$ as the feature of interest.

| | $IC_{50,I_{Kr}}$ | $IC_{50,I_{CaV}}$ | $IC_{50,I_{Na}}$ | $EFTPC$ | $Target$ |
|---|---|---|---|---|---|
| Ajmaline | 1040.0 | 71000 | 8200.0 | 1500.00 | 1.0 |
| Amiodarone | 30.0 | 270 | 4800.0 | 0.50 | 1.0 |
| Amitriptyline | 3280.0 | 11600 | 20000.0 | 41.00 | 0.0 |
| Bepridil | 33.0 | 211 | 3700.0 | 33.00 | 1.0 |
| Chlorpromazine | 1470.0 | - | 4300.0 | 38.00 | 1.0 |
| Cibenzoline | 22600.0 | 30000 | 7800.0 | 976.00 | 0.0 |
| Cisapride | 6.5 | - | 14700.0 | 4.90 | 1.0 |
| Desipramine | 1390.0 | 1709 | 1520.0 | 108.00 | 0.0 |
| Diltiazem | 17300.0 | 450 | 9000.0 | 122.00 | 0.0 |
| Diphenhydramine | 5200.0 | 228000 | 41000.0 | 34.00 | 0.0 |
| Dofetilide | 5.0 | 60000 | 300000.0 | 2.00 | 1.0 |
| Fluvoxamine | 3100.0 | 4900 | 39400.0 | 377.00 | 0.0 |
| Haloperidol | 27.0 | 1700 | 7000.0 | 3.60 | 1.0 |
| Imipramine | 3400.0 | 8300 | 3600.0 | 106.00 | 0.0 |
| Mexiletine | 50000.0 | 100000 | 43000.0 | 4129.00 | 0.0 |
| Mibefradil | 1800.0 | 156 | 980.0 | 12.00 | 0.0 |
| Nifedipine | 275000.0 | 60 | 37000.0 | 7.70 | 0.0 |
| Nitrendipine | 10000.0 | 0.35 | 36000.0 | 3.02 | 0.0 |
| Phenytoin | 100000.0 | 103000 | 49000.0 | 4500.00 | 0.0 |
| Pimozide | 20.0 | 162 | 54.0 | 1.00 | 1.0 |
| Prenylamine | 65.0 | 1240 | 2520.0 | 17.00 | 1.0 |
| Propafenone | 440.0 | 1800 | 1190.0 | 241.00 | 0.0 |
| Propranolol | 2828.0 | 18000 | 2100.0 | 26.00 | 0.0 |
| Quetiapine | 5800.0 | 10400 | 16900.0 | 33.00 | 0.0 |
| Quinidine | 300.0 | 15600 | 16600.0 | 3237.00 | 1.0 |
| Risperidone | 150.0 | 73000 | 102000.0 | 1.81 | 0.0 |
| Sertindole | 14.0 | 8900 | 2300.0 | 1.59 | 1.0 |
| Tedisamil | 2500.0 | - | 20000.0 | 85.00 | 1.0 |
| Terfenadine | 8.9 | 375 | 971.0 | 9.00 | 1.0 |
| Thioridazine | 33.0 | 1300 | 1830.0 | 979.00 | 1.0 |
| Verapamil | 143.0 | 100 | 41500.0 | 81.00 | 0.0 |

**Table S1.** $IC_{50}$, Hill coefficients, EFTPC values and risk categorization for the (Mirams et al., 2011) dataset. 0: TdP- 1: TdP+

**Dataset 2: Kramer et al. 2013** (Kramer et al., 2013). *In-vitro* ion channel screening of drug-induced effects on $I_{Kr}$, $I_{Na,fast}$ and $I_{CaV}$ was carried out for 55 compounds using automated patch clamp systems. Logistic regression classifiers were built on negative log transformed EFTPC indexes (i.e., ratio of $IC_{50}$ for channel block to the EFTPC). Drugs in R1, R2, R3, CM1, CM2 and CM3 groups or drugs with package inserts containing a warning for TdP were defined as TdP+.

| | $IC_{50,I_{Kr}}$ | $IC_{50,I_{CaV}}$ | $IC_{50,I_{Na}}$ | $h_{I_{Kr}}$ | $h_{I_{CaV}}$ | $h_{I_{Na}}$ | $EFTPC$ | $Target$ |
|---|---|---|---|---|---|---|---|---|
| Amiodarone | 860.0 | 1900.0 | 15900.0 | 1.09 | 0.69 | 0.97 | 0.8 | 1.0 |
| Astemizole | 4.0 | 1100.0 | 3000.0 | 0.78 | 1.66 | 1.95 | 0.3 | 1.0 |
| Bepridil | 160.0 | 1000.0 | 2300.0 | 0.88 | 1.28 | 1.26 | 35.0 | 1.0 |
| Ceftriaxone | 445700.0 | 153800.0 | 555900.0 | 1.00 | 1.00 | 1.00 | 23170.0 | 0.0 |
| Chlorpromazine | 1500.0 | 3400.0 | 3000.0 | 1.40 | 1.73 | 1.14 | 38.0 | 1.0 |
| Cilostazol | 13800.0 | 91200.0 | 93700.0 | 0.91 | 1.00 | 1.00 | 128.0 | 1.0 |
| Cisapride | 20.0 | 11800.0 | 337000.0 | 1.04 | 1.00 | 1.00 | 3.0 | 1.0 |
| Clozapine | 2300.0 | 3600.0 | 15100.0 | 0.97 | 1.00 | 1.14 | 71.0 | 1.0 |
| Dosatinib | 24500.0 | 81100.0 | 76300.0 | 1.16 | 1.00 | 1.43 | 41.0 | 0.0 |
| Diazepam | 53200.0 | 30500.0 | 306400.0 | 1.07 | 0.89 | 1.00 | 29.0 | 0.0 |
| Diltiazem | 13200.0 | 760.0 | 22400.0 | 1.16 | 1.14 | 1.29 | 122.0 | 0.0 |
| Disopyramide | 14400.0 | 1036700.0 | 168400.0 | 0.91 | 1.00 | 1.09 | 742.0 | 1.0 |
| Dofetilide | 30.0 | 26700.0 | 162100.0 | 1.20 | 1.00 | 1.00 | 2.0 | 1.0 |
| Donepezil | 700.0 | 34300.0 | 38500.0 | 0.98 | 0.83 | 1.00 | 3.0 | 0.0 |
| Droperidol | 60.0 | 7600.0 | 22700.0 | 1.10 | 1.16 | 1.24 | 16.0 | 1.0 |
| Duloxetine | 3800.0 | 2800.0 | 5100.0 | 1.39 | 1.41 | 1.66 | 16.0 | 0.0 |
| Flecainide | 1500.0 | 27100.0 | 6200.0 | 0.88 | 0.97 | 1.14 | 753.0 | 1.0 |
| Halofantrine | 380.0 | 1900.0 | 331200.0 | 1.31 | 0.99 | 1.00 | 172.0 | 1.0 |
| Haloperidol | 40.0 | 1300.0 | 4300.0 | 1.18 | 1.38 | 1.58 | 4.0 | 1.0 |
| Ibutilide | 18.0 | 62500.0 | 42500.0 | 1.53 | 1.16 | 1.03 | 140.0 | 1.0 |
| Lamivudine | 2054000.0 | 54200.0 | 1571400.0 | 1.00 | 0.89 | 1.00 | 19540.0 | 0.0 |
| Linezolid | 1147200.0 | 105400.0 | 2644500.0 | 1.00 | 0.94 | 1.00 | 59110.0 | 0.0 |
| Loratadine | 6100.0 | 11400.0 | 28900.0 | 1.44 | 1.38 | 1.64 | 0.4 | 0.0 |
| Methadone | 3500.0 | 37400.0 | 31800.0 | 1.00 | 1.67 | 1.37 | 507.0 | 1.0 |
| Metronidazole | 1340200.0 | 177900.0 | 2073200.0 | 1.00 | 0.66 | 1.00 | 187000.0 | 0.0 |
| Mibefradil | 1700.0 | 510.0 | 5600.0 | 1.38 | 1.44 | 1.53 | 12.0 | 0.0 |
| Mitoxantrone | 539400.0 | 22500.0 | 93500.0 | 1.00 | 0.64 | 1.05 | 225.0 | 0.0 |
| Moxifloxacin | 86200.0 | 173000.0 | 1112000.0 | 0.94 | 1.00 | 1.00 | 10960.0 | 1.0 |
| Nifedipine | 44000.0 | 12.0 | 88500.0 | 0.80 | 1.02 | 0.71 | 8.0 | 0.0 |
| Nilotinib | 1000.0 | 17500.0 | 13300.0 | 0.96 | 1.00 | 2.11 | 172.0 | 1.0 |
| Nitrendipine | 24600.0 | 25.0 | 21600.0 | 0.82 | 0.78 | 1.25 | 3.0 | 0.0 |
| Paliperidone | 780.0 | 193900.0 | 109000.0 | 1.01 | 1.00 | 1.33 | 69.0 | 1.0 |
| Paroxetine | 1900.0 | 3900.0 | 9800.0 | 1.26 | 1.39 | 1.34 | 14.0 | 1.0 |
| Pentobarbital | 1433900.0 | 299000.0 | 2686000.0 | 1.00 | 1.38 | 1.00 | 5171.0 | 0.0 |
| Phenytoin | 147000.0 | 21900.0 | 72400.0 | 1.00 | 0.99 | 1.06 | 4360.0 | 0.0 |
| Pimozide | 40.0 | 240.0 | 1100.0 | 1.16 | 1.49 | 1.05 | 0.5 | 1.0 |
| Piperacillin | 3405100.0 | 1226000.0 | 2433800.0 | 1.00 | 1.00 | 1.00 | 1378000.0 | 0.0 |
| Procainamide | 272400.0 | 389500.0 | 746600.0 | 1.00 | 0.83 | 1.00 | 54180.0 | 1.0 |
| Quinidine | 720.0 | 6400.0 | 14600.0 | 1.06 | 0.68 | 1.22 | 3237.0 | 1.0 |
| Raltegravir | 782800.0 | 246700.0 | 842200.0 | 1.00 | 1.00 | 1.00 | 7000.0 | 0.0 |
| Ribavirin | 967000.0 | 622500.0 | 2997500.0 | 1.00 | 1.00 | 1.00 | 27880.0 | 0.0 |
| Risperidone | 260.0 | 34200.0 | 43400.0 | 0.99 | 0.79 | 0.98 | 2.0 | 1.0 |
| Saquinavir | 16900.0 | 1900.0 | 12100.0 | 1.72 | 1.15 | 2.34 | 130.0 | 0.0 |
| Sertindole | 33.0 | 6300.0 | 6900.0 | 1.25 | 1.29 | 1.19 | 2.0 | 1.0 |
| Sitagliptin | 174700.0 | 147100.0 | 1220800.0 | 1.00 | 1.00 | 1.00 | 442.0 | 0.0 |
| Solifenacin | 280.0 | 4300.0 | 1500.0 | 0.90 | 1.47 | 1.32 | 3.0 | 1.0 |
| Sotalol | 111400.0 | 193300.0 | 7013900.0 | 0.73 | 1.00 | 1.00 | 14690.0 | 1.0 |
| Sparfloxacin | 22100.0 | 88800.0 | 2555000.0 | 0.93 | 1.00 | 1.00 | 1766.0 | 1.0 |
| Sunitinib | 1200.0 | 33400.0 | 16500.0 | 1.00 | 1.09 | 1.22 | 13.0 | 1.0 |
| Telbivudine | 422700.0 | 713900.0 | 1095200.0 | 1.00 | 1.00 | 1.00 | 19720.0 | 0.0 |
| Terfenadine | 50.0 | 930.0 | 2000.0 | 1.25 | 1.80 | 1.81 | 9.0 | 1.0 |
| Terodiline | 650.0 | 4800.0 | 7400.0 | 1.02 | 1.01 | 1.23 | 145.0 | 1.0 |
| Thioridazine | 500.0 | 3500.0 | 1400.0 | 0.98 | 1.35 | 1.11 | 980.0 | 1.0 |
| Verapamil | 250.0 | 200.0 | 32500.0 | 0.89 | 0.80 | 1.33 | 88.0 | 0.0 |
| Voriconazole | 490900.0 | 414200.0 | 1550500.0 | 1.00 | 1.00 | 1.00 | 7563.0 | 1.0 |

**Table S2.** $IC_{50}$, Hill coefficients, EFTPC values and risk categorization for the (Kramer et al., 2013) dataset. Binary risk categorization - 0:TdP- 1: TdP+

**Dataset 3: Okada et al. 2015** (Okada et al., 2015). *In-vitro* ion channel assay was carried out on the Chinese hamster ovary cells to examine the modulation of six ion channel currents ($I_{Kr}$, $I_{Na,fast}$, $I_{CaV}$, $I_{to}$, $I_{Ks}$, and $I_{K1}$) under the influence of 12 drugs. Late sodium current block was examined for Ranolazine only. The TdP risk of the drugs was defined based on the categories in RedFern et al. (Redfern et al., 2003) with compounds in R1, R2 and R3 categories as TdP+. For the drugs not listed in Redfern et al., other literature was utilized to determine the drugs TdP risk. A 3-D heart model coupled with a torso model was utilized to simulate the drug-induced effects at drug concentrations ranging from EFTPC to 200x EFTPC. Drugs were classified as torsadogenic if arrhythmias were observed in the computational model below 200x EFTPC drug concentrations.

| | $IC_{50,I_{Kr}}$ | $IC_{50,I_{CaV}}$ | $IC_{50,I_{Na,peak}}$ | $IC_{50,I_{Ks}}$ | $IC_{50,I_{K1}}$ | $IC_{50,I_{to}}$ | $IC_{50,I_{Na,late}}$ | $h_{I_{Kr}}$ | $h_{I_{CaV}}$ | $h_{I_{Na,peak}}$ | $h_{I_{Ks}}$ | $h_{I_{K1}}$ | $h_{I_{to}}$ | $h_{I_{Na,late}}$ | $EFTPC$ | $Target$ |
|---|---|---|---|---|---|---|---|---|---|---|---|---|---|---|---|---|
| Amiodarone | 755.70 | 4832 | 975.4 | - | - | - | - | 0.817 | 0.850 | 0.746 | 1.000 | 1.0 | 1.000 | 1.0 | 0.775 | 0.0 |
| Astemizole | 28.12 | 987.8 | 1862 | - | - | - | - | 1.745 | 2.531 | 1.532 | 1.000 | 1.0 | 1.000 | 1.0 | 0.288 | 1.0 |
| Bepridil | 130.20 | 1455 | 646.5 | 6031.2 | - | 4521 | - | 1.461 | 2.317 | 1.155 | 1.779 | 1.0 | 1.853 | 1.0 | 34.630 | 1.0 |
| Cisapride | 14.72 | 4278 | 2072 | - | - | - | - | 1.326 | 1.378 | 0.894 | 1.000 | 1.0 | 1.000 | 1.0 | 2.579 | 1.0 |
| Dofetilide | 38.28 | 184100 | 124500 | - | - | - | - | 1.983 | 0.897 | 0.329 | 1.000 | 1.0 | 1.000 | 1.0 | 1.600 | 1.0 |
| E4031 | 26.27 | 713700 | 737300 | - | - | - | - | 1.996 | 0.454 | 0.334 | 1.000 | 1.0 | 1.000 | 1.0 | 5.668 | 1.0 |
| Moxifloxacin | 135800.00 | 469700 | 2.721e+06 | 2.011e+06 | - | 2.624e+06 | - | 0.910 | 1.549 | 2.173 | 1.173 | 1.0 | 0.687 | 1.0 | 10950.000 | 0.0 |
| Quinidine | 637.70 | 7731 | 24790 | 73330 | - | 1906 | - | 1.012 | 0.823 | 1.300 | 1.336 | 1.0 | 1.198 | 1.0 | 3235.000 | 1.0 |
| Ranolazine | 3930.00 | 118300 | 41115 | - | - | 433510 | 26260 | 0.710 | 0.894 | 0.904 | 1.000 | 1.0 | 1.309 | 1.5 | 2310.000 | 0.0 |
| Sotalol | 356400.00 | - | - | - | - | - | - | 1.023 | 1.000 | 1.000 | 1.000 | 1.0 | 1.000 | 1.0 | 14680.000 | 1.0 |
| Terfenadine | 98.52 | 861.5 | 479.8 | - | - | - | - | 1.176 | 2.091 | 0.516 | 1.000 | 1.0 | 1.000 | 1.0 | 9.000 | 1.0 |
| Verapamil | 201.30 | 333.1 | 4272 | 29880 | - | 3523 | - | 1.040 | 1.148 | 0.850 | 0.933 | 1.0 | 0.992 | 1.0 | 87.970 | 0.0 |

**Table S3.** $IC_{50}$, Hill coefficients, EFTPC values and risk categorization for the (Okada et al., 2015) dataset. Target column represents the binary risk categorization assigend in Okada et al., 0: TdP- 1: TdP+

**Dataset 4: Lancaster and Sobie. 2016** (Lancaster and Sobie, 2016). The dataset consist of 86 compounds (data from Dataset 1 and 2 combined) with $IC_{50}$ values for $I_{Kr}$, $I_{Na,fast}$ and $I_{CaV}$. Drugs in the group CM1 in AZCERT and the category CH1 in (Champeroux et al., 2005) were defined as torsadogenic (TdP+). Drugs in the AZCERT CM2 and CM3 groups were defined as torsadogenic (TdP+) only if a warning of TdP appeared in the package insert. The effects of drug-induced multi-channel block were simulated using computational models of the cardiac cells. Classifiers built on $APD_{50}$ and $Diastolic - Ca^{2+}$ levels provided the highest classification accuracy scores.

| | $IC_{50,I_{Kr}}$ | $IC_{50,I_{CaV}}$ | $IC_{50,I_{Na}}$ | $h_{I_{Kr}}$ | $h_{I_{CaV}}$ | $h_{I_{Na}}$ | $EFTPC$ | $Target$ |
|---|---|---|---|---|---|---|---|---|
| Ajmaline | 1040.0 | 71000 | 8200.0 | 1.00 | 1.00 | 1.00 | 1500.00 | 1.0 |
| Amiodarone1 | 860.0 | 1900 | 15900.0 | 1.09 | 0.69 | 0.97 | 0.80 | 1.0 |
| Amiodarone2 | 30.0 | 270 | 4800.0 | 1.00 | 1.00 | 1.00 | 0.50 | 1.0 |
| Amitriptyline | 3280.0 | 11600 | 20000.0 | 1.00 | 1.00 | 1.00 | 41.00 | 0.0 |
| Astemizole | 4.0 | 1100 | 3000.0 | 0.78 | 1.66 | 1.95 | 0.30 | 1.0 |
| Bepridil1 | 160.0 | 1000 | 2300.0 | 0.88 | 1.28 | 1.26 | 35.00 | 1.0 |
| Bepridil2 | 33.0 | 211 | 3700.0 | 1.00 | 1.00 | 1.00 | 33.00 | 1.0 |
| Ceftriaxone | 445700.0 | 153800 | 555900.0 | 1.00 | 1.00 | 1.00 | 23170.00 | 0.0 |
| Chlorpromazine1 | 1500.0 | 3400 | 3000.0 | 1.40 | 1.73 | 1.14 | 38.00 | 1.0 |
| Chlorpromazine2 | 1470.0 | - | 4300.0 | 1.00 | 1.00 | 1.00 | 38.00 | 1.0 |
| Cibenzoline | 22600.0 | 30000 | 7800.0 | 1.00 | 1.00 | 1.00 | 976.00 | 0.0 |
| Cilostazol | 13800.0 | 91200 | 93700.0 | 0.91 | 1.00 | 1.00 | 128.00 | 1.0 |
| Cisapride1 | 20.0 | 11800 | 337000.0 | 1.04 | 1.00 | 1.00 | 3.00 | 1.0 |
| Cisapride2 | 6500.0 | - | 14700.0 | 1.00 | 1.00 | 1.00 | 4.90 | 1.0 |
| Clozapine | 2300.0 | 3600 | 15100.0 | 0.97 | 1.00 | 1.14 | 71.00 | 1.0 |
| Dosatinib | 24500.0 | 81100 | 76300.0 | 1.16 | 1.00 | 1.43 | 41.00 | 0.0 |
| Desipramine | 1390.0 | 1709 | 1520.0 | 1.00 | 1.00 | 1.00 | 108.00 | 0.0 |
| Diazepam | 53200.0 | 30500 | 306400.0 | 1.07 | 0.89 | 1.00 | 29.00 | 0.0 |
| Diltiazem1 | 13200.0 | 760 | 22400.0 | 1.16 | 1.14 | 1.29 | 122.00 | 0.0 |
| Diltiazem2 | 17300.0 | 450 | 9000.0 | 1.00 | 1.00 | 1.00 | 122.00 | 0.0 |
| Diphenhydramine | 5200.0 | 228000 | 41000.0 | 1.00 | 1.00 | 1.00 | 34.00 | 0.0 |
| Disopyramide | 14400.0 | 1.0367e+06 | 168400.0 | 0.91 | 1.00 | 1.09 | 742.00 | 1.0 |
| Dofetilide1 | 30.0 | 26700 | 162100.0 | 1.20 | 1.00 | 1.00 | 2.00 | 1.0 |
| Dofetilide2 | 5.0 | 60000 | 300000.0 | 1.00 | 1.00 | 1.00 | 2.00 | 1.0 |
| Donepezil | 700.0 | 34300 | 38500.0 | 0.98 | 0.83 | 1.00 | 3.00 | 0.0 |
| Droperidol | 60.0 | 7600 | 22700.0 | 1.10 | 1.16 | 1.24 | 16.00 | 1.0 |
| Duloxetine | 3800.0 | 2800 | 5100.0 | 1.39 | 1.41 | 1.66 | 16.00 | 0.0 |
| Flecainide | 1500.0 | 27100 | 6200.0 | 0.88 | 0.97 | 1.14 | 753.00 | 1.0 |
| Fluvoxamine | 3100.0 | 4900 | 39400.0 | 1.00 | 1.00 | 1.00 | 377.00 | 0.0 |
| Halofantrine | 380.0 | 1900 | 331200.0 | 1.31 | 0.99 | 1.00 | 172.00 | 1.0 |
| Haloperidol1 | 40.0 | 1300 | 4300.0 | 1.18 | 1.38 | 1.58 | 4.00 | 1.0 |
| Haloperidol2 | 27.0 | 1700 | 7000.0 | 1.00 | 1.00 | 1.00 | 3.60 | 1.0 |
| Ibutilide | 18.0 | 62500 | 42500.0 | 1.53 | 1.16 | 1.03 | 140.00 | 1.0 |
| Imipramine | 3400.0 | 8300 | 3600.0 | 1.00 | 1.00 | 1.00 | 106.00 | 1.0 |
| Lamivudine | 2054000.0 | 54200 | 1571400.0 | 1.00 | 0.89 | 1.00 | 19540.00 | 0.0 |
| Linezolid | 1147200.0 | 105400 | 2644500.0 | 1.00 | 0.94 | 1.00 | 59110.00 | 0.0 |
| Loratadine | 6100.0 | 11400 | 28900.0 | 1.44 | 1.38 | 1.64 | 0.40 | 0.0 |
| Methadone | 3500.0 | 37400 | 31800.0 | 1.00 | 1.67 | 1.37 | 507.00 | 1.0 |
| Metronidazole | 1340200.0 | 177900 | 2073200.0 | 1.00 | 0.66 | 1.00 | 187000.00 | 0.0 |
| Mexiletine | 50000.0 | 100000 | 43000.0 | 1.00 | 1.00 | 1.00 | 4129.00 | 0.0 |
| Mibefradil1 | 1700.0 | 510 | 5600.0 | 1.38 | 1.44 | 1.53 | 12.00 | 0.0 |
| Mibefradil2 | 1800.0 | 156 | 980.0 | 1.00 | 1.00 | 1.00 | 12.00 | 0.0 |
| Mitoxantrone | 539400.0 | 22500 | 93500.0 | 1.00 | 0.64 | 1.05 | 225.00 | 0.0 |
| Moxifloxacin | 86200.0 | 173000 | 1112000.0 | 0.94 | 1.00 | 1.00 | 10960.00 | 1.0 |
| Nifedipine1 | 44000.0 | 12 | 88500.0 | 0.80 | 1.02 | 0.71 | 8.00 | 0.0 |
| Nifedipine2 | 275000.0 | 60 | 37000.0 | 1.00 | 1.00 | 1.00 | 7.70 | 0.0 |
| Nilotinib | 1000.0 | 17500 | 13300.0 | 0.96 | 1.00 | 2.11 | 172.00 | 1.0 |
| Nitrendipine1 | 24600.0 | 25 | 21600.0 | 0.82 | 0.78 | 1.25 | 3.00 | 0.0 |
| Nitrendipine2 | 10000.0 | 0.35 | 36000.0 | 1.00 | 1.00 | 1.00 | 3.02 | 0.0 |
| Paliperidone | 780.0 | 193900 | 109000.0 | 1.01 | 1.00 | 1.33 | 69.00 | 1.0 |
| Paroxetine | 1900.0 | 3900 | 9800.0 | 1.26 | 1.39 | 1.34 | 14.00 | 1.0 |
| Pentobarbital | 1433900.0 | 299000 | 2686000.0 | 1.00 | 1.38 | 1.00 | 5171.00 | 0.0 |
| Phenytoin1 | 147000.0 | 21900 | 72400.0 | 1.00 | 0.99 | 1.06 | 4360.00 | 0.0 |
| Phenytoin2 | 100000.0 | 103000 | 49000.0 | 1.00 | 1.00 | 1.00 | 4500.00 | 0.0 |
| Pimozide1 | 40.0 | 240 | 1100.0 | 1.16 | 1.49 | 1.05 | 0.50 | 1.0 |
| Pimozide2 | 20.0 | 162 | 54.0 | 1.00 | 1.00 | 1.00 | 1.00 | 1.0 |
| Piperacillin | 3405100.0 | 1.226e+06 | 2433800.0 | 1.00 | 1.00 | 1.00 | 1378000.00 | 0.0 |
| Prenylamine | 65.0 | 1240 | 2520.0 | 1.00 | 1.00 | 1.00 | 17.00 | 1.0 |
| Procainamide | 272400.0 | 389500 | 746600.0 | 1.00 | 0.83 | 1.00 | 54180.00 | 1.0 |
| Propafenone | 440.0 | 1800 | 1190.0 | 1.00 | 1.00 | 1.00 | 241.00 | 1.0 |
| Propranolol | 2828.0 | 18000 | 2100.0 | 1.00 | 1.00 | 1.00 | 26.00 | 0.0 |
| Quetiapine | 5800.0 | 10400 | 16900.0 | 1.00 | 1.00 | 1.00 | 33.00 | 0.0 |
| Quinidine1 | 720.0 | 6400 | 14600.0 | 1.06 | 0.68 | 1.22 | 3237.00 | 1.0 |
| Quinidine2 | 300.0 | 15600 | 16600.0 | 1.00 | 1.00 | 1.00 | 924.00 | 1.0 |
| Raltegravir | 782800.0 | 246700 | 842200.0 | 1.00 | 1.00 | 1.00 | 7000.00 | 0.0 |
| Ribavirin | 967000.0 | 622500 | 2997500.0 | 1.00 | 1.00 | 1.00 | 27880.00 | 0.0 |
| Risperidone1 | 260.0 | 34200 | 43400.0 | 0.99 | 0.79 | 0.98 | 2.00 | 1.0 |
| Risperidone2 | 150.0 | 73000 | 102000.0 | 1.00 | 1.00 | 1.00 | 1.81 | 1.0 |
| Saquinavir | 16900.0 | 1900 | 12100.0 | 1.72 | 1.15 | 2.34 | 130.00 | 0.0 |
| Sertindole1 | 33.0 | 6300 | 6900.0 | 1.25 | 1.29 | 1.19 | 2.00 | 1.0 |
| Sertindole2 | 14.0 | 8900 | 2300.0 | 1.00 | 1.00 | 1.00 | 1.59 | 1.0 |
| Sitagliptin | 174700.0 | 147100 | 1220800.0 | 1.00 | 1.00 | 1.00 | 442.00 | 0.0 |
| Solifenacin | 280.0 | 4300 | 1500.0 | 0.90 | 1.47 | 1.32 | 3.00 | 1.0 |
| Sotalol | 111400.0 | 193300 | 7013900.0 | 0.73 | 1.00 | 1.00 | 14690.00 | 1.0 |
| Sparfloxacin | 22100.0 | 88800 | 2555000.0 | 0.93 | 1.00 | 1.00 | 1766.00 | 1.0 |
| Sunitinib | 1200.0 | 33400 | 16500.0 | 1.00 | 1.09 | 1.22 | 13.00 | 1.0 |
| Tedisamil | 2500.0 | - | 20000.0 | 1.00 | 1.00 | 1.00 | 85.00 | 1.0 |
| Telbivudine | 422700.0 | 713900 | 1095200.0 | 1.00 | 1.00 | 1.00 | 19720.00 | 0.0 |
| Terfenadine1 | 50.0 | 930 | 2000.0 | 1.25 | 1.80 | 1.81 | 9.00 | 1.0 |
| Terfenadine2 | 8.9 | 375 | 971.0 | 1.00 | 1.00 | 1.00 | 9.00 | 1.0 |
| Terodiline | 33.0 | 6300 | 6900.0 | 1.02 | 1.01 | 1.23 | 2.00 | 1.0 |
| Thioridazine1 | 500.0 | 3500 | 1400.0 | 0.98 | 1.35 | 1.11 | 980.00 | 1.0 |
| Thioridazine2 | 33.0 | 1300 | 1830.0 | 1.00 | 1.00 | 1.00 | 208.00 | 1.0 |
| Verapamil1 | 250.0 | 200 | 32500.0 | 0.89 | 0.80 | 1.33 | 88.00 | 0.0 |
| Verapamil2 | 143.0 | 100 | 41500.0 | 1.00 | 1.00 | 1.00 | 81.00 | 0.0 |
| Voriconazole | 490900.0 | 414200 | 1550500.0 | 1.00 | 1.00 | 1.00 | 7563.00 | 1.0 |

**Table S4.** $IC_{50}$, Hill coefficients, EFTPC values and risk categorization for the (Lancaster and Sobie, 2016) dataset. Target column represents the binary risk categorization assigned in Lancaster and Sobie., 0: TdP- 1: TdP+

**Dataset 5: Crumb et al. 2016** (Crumb et al., 2016). 30 compounds were analyzed using *in-vitro* ion channel screening for their potencies to block seven ion-channel currents ($I_{Kr}$, $I_{Na,fast}$, $I_{NaL}$ $I_{CaV}$, $I_{to}$, $I_{Ks}$, and $I_{K1}$) using manual patch clamps. Effects of the block of different ion channel currents for these 12 (out of 30) drugs were compared, and important roles of $I_{NaL}$ and $I_{CaV}$, in addition to $I_{Kr}$, in TdP risk prediction were suggested. However, no statistical analysis was performed.

| | $IC_{50,I_{Kr}}$ | $IC_{50,I_{CaV}}$ | $IC_{50,I_{Na,peak}}$ | $IC_{50,I_{Ks}}$ | $IC_{50,I_{K1}}$ | $IC_{50,I_{to}}$ | $IC_{50,I_{Na,late}}$ | $h_{I_{Kr}}$ | $h_{I_{CaV}}$ | $h_{I_{Na,peak}}$ | $h_{I_{Ks}}$ | $h_{I_{K1}}$ | $h_{I_{to}}$ | $h_{I_{NaL}}$ | $EFTPC$ | $Target$ |
|---|---|---|---|---|---|---|---|---|---|---|---|---|---|---|---|---|
| Amiodarone | 941 | 1281 | 4577 | 5666 | - | 3758 | 9423 | 0.6 | 0.6 | 0.7 | 1.0 | 1.0 | 0.4 | 0.4 | 0.7 | 1.0 |
| Amitriptyline | 3660 | 1291 | 5760 | 2737 | - | 2543 | 4433 | 1.0 | 1.0 | 1.3 | 0.5 | 1.0 | 0.4 | 0.5 | 36.4 | 0.0 |
| Azithromycin | 70796 | - | - | 470131 | - | 88764 | 189128 | 0.5 | 1.0 | 1.0 | 1.4 | 1.0 | 0.5 | 1.9 | 1937.0 | 1.0 |
| Bepridil | 149 | 2808 | 2929 | 28628.3 | - | 8594 | 1814 | 0.9 | 0.6 | 1.2 | 0.7 | 1.0 | 3.5 | 1.4 | 31.5 | 1.0 |
| Chloroquine | 6889 | - | 80000 | - | 10595 | - | 70000 | 0.6 | 1.0 | 1.0 | 1.0 | 0.8 | 1.0 | 1.0 | 249.5 | 1.0 |
| Chlorpromazine | 1118 | 8192 | 4536 | - | 9270 | 1.76167e+07 | 4560 | 0.9 | 0.8 | 2.0 | 1.0 | 0.7 | 0.4 | 0.9 | 34.5 | 1.0 |
| Cibenzoline | 2097 | - | 21752 | - | - | - | 46581 | 0.9 | 1.0 | 1.2 | 1.0 | 1.0 | 1.0 | 0.6 | 673.0 | 0.0 |
| Cisapride | 12 | 9.25808e+06 | 1e+10 | 8.11929e+07 | 29498 | 219112 | - | 1.3 | 0.4 | 1.0 | 0.3 | 0.5 | 0.2 | 1.0 | 2.6 | 1.0 |
| Diltiazem | 6569 | 112 | 110859 | - | - | 2.8e+09 | 21862.5 | 0.8 | 0.7 | 0.7 | 1.0 | 1.0 | 0.2 | 0.7 | 127.5 | 0.0 |
| Dofetilide | 1 | 260.3 | - | - | 394.3 | 18.8 | 753160 | 0.6 | 1.0 | 0.9 | 1.0 | 0.8 | 0.8 | 1.0 | 2.1 | 1.0 |
| Flecainide | 692 | 25599 | 6677 | - | - | 9266 | 18870 | 0.8 | 1.4 | 1.9 | 1.0 | 1.0 | 0.7 | 0.6 | 752.9 | 1.0 |
| Lidocaine | - | - | - | - | - | - | 10790 | 1.0 | 1.0 | 1.0 | 1.0 | 1.0 | 1.0 | 1.3 | 2560.0 | 0.0 |
| Lopinavir | 5170 | 15601 | - | - | - | - | - | 1.2 | 1.0 | 1.0 | 1.0 | 1.0 | 1.0 | 1.0 | 703.7 | 1.0 |
| Mexiletine | 80000 | 40000 | - | - | - | - | 8957 | 1.0 | 1.0 | 1.0 | 1.0 | 1.0 | 1.0 | 1.4 | 2503.2 | 0.0 |
| Mibefradil | 307 | 652 | 5866 | - | - | - | 3628 | 0.9 | 1.1 | 1.0 | 1.0 | 1.0 | 1.0 | 1.2 | 10.6 | 0.0 |
| Moxifloxacin | 93041 | - | - | 50321 | - | - | 382337 | 0.6 | 1.0 | 1.0 | 1.0 | 1.0 | 1.0 | 1.1 | 3562.5 | 1.0 |
| Nilotinib | 91 | - | - | - | - | - | 4706 | 0.8 | 1.0 | 1.0 | 1.0 | 1.0 | 1.0 | 1.0 | 60.4 | 1.0 |
| Ondansetron | 1492 | 22551 | 57666.4 | 569807 | - | 1.02338e+06 | 19181 | 1.0 | 0.8 | 1.0 | 0.7 | 1.0 | 1.0 | 1.0 | 358.5 | 1.0 |
| Propafenone | 481 | 1550 | 3886 | - | - | - | 4036 | 0.8 | 0.9 | 1.0 | 1.0 | 1.0 | 1.0 | 0.9 | 131.0 | 1.0 |
| Quinidine | 343 | 51592.3 | 12329 | 4899 | 3.95899e+07 | 3487 | 9417 | 1.0 | 1.0 | 1.5 | 1.4 | 0.4 | 1.3 | 1.0 | 842.9 | 1.0 |
| Quinine | 5170 | 27178 | 24151 | 37453 | - | 79254 | 11053 | 1.0 | 1.0 | 1.1 | 1.1 | 1.0 | 1.0 | 0.4 | 3956.7 | 1.0 |
| Ranolazine | 6490 | - | - | 3.6155e+07 | - | - | 7884 | 0.8 | 1.0 | 1.0 | 0.5 | 1.0 | 1.0 | 0.9 | 1948.2 | 0.0 |
| Ritonavir | 5157 | 8228 | 17000 | - | - | - | 7175 | 1.0 | 1.3 | 1.0 | 1.0 | 1.0 | 1.0 | 0.7 | 436.9 | 1.0 |
| Rufinamide | - | 264448 | - | - | - | - | - | 1.0 | 0.9 | 1.0 | 1.0 | 1.0 | 1.0 | 1.0 | 83126.9 | 0.0 |
| Saquinavir | 3477 | 3161 | 15568 | - | - | - | 7088 | 1.0 | 1.4 | 1.2 | 1.0 | 1.0 | 1.0 | 1.2 | 417.2 | 1.0 |
| Sertindole | 11 | - | - | - | - | - | - | 0.8 | 1.0 | 1.0 | 1.0 | 1.0 | 1.0 | 1.0 | 1.6 | 1.0 |
| Sotalol | 86369 | 7.06153e+06 | 1.14e+09 | 4.22186e+06 | 3.05026e+06 | 4.31435e+07 | - | 0.9 | 0.9 | 0.5 | 1.2 | 1.2 | 0.7 | 1.0 | 14686.4 | 1.0 |
| Terfenadine | 19 | 700 | 4803 | 399754 | - | 239961 | 20056 | 0.6 | 0.7 | 1.0 | 0.5 | 1.0 | 0.3 | 0.6 | 9.0 | 1.0 |
| Toremifene | 3500 | - | - | - | - | - | 4000 | 1.0 | 1.0 | 1.0 | 1.0 | 1.0 | 1.0 | 1.0 | 26.3 | 1.0 |
| Verapamil | 499 | 202 | - | - | 3.49e+08 | 13429.2 | 7028 | 1.1 | 1.1 | 1.0 | 1.0 | 0.3 | 0.8 | 1.0 | 45.0 | 0.0 |

**Table S5.** $IC_{50}$, Hill coefficients, EFTPC values, and risk categorization for the (Crumb et al., 2016) dataset. Binary risk categorization based on table (Table S11), 0: TdP- 1: TdP+

**Dataset 6: Ando et al. 2017** (Ando et al., 2017). Drug-induced effects of 57 compounds were evaluated through recording of extracellular field potentials in human induced pluripotent stem cell-derived cardiomyocytes (hiPSC-CMs). A two-dimensional risk map with risk potential according to the degree of FP duration prolongation and early afterdepolarization occurrence in one dimension and risk margin based on the ratio of the free concentration of the drug in medium and EFPTC in another dimension provided the high classification accuracies. $IC_{50}$ values for drug-induced blocks of $I_{Kr}$, $I_{Na,fast}$ and $I_{CaL}$ were also provided. Drugs were classified into three categories (high, intermediate, and low risk); and 36 drugs that were assigned to low and high risk were validated against the CredibleMeds categorization. Drugs in CM1 category were considered TdP+ for validation, and the remainder were considered TdP-.

| | $IC_{50,I_{Kr}}$ | $IC_{50,I_{CaV}}$ | $IC_{50,I_{Na}}$ | $EFTPC$ | $Target$ |
|---|---|---|---|---|---|
| Ajmaline | 1040.0 | 71000 | 8200 | 25.00 | 0.0 |
| Amiodarone | 860.0 | 1900 | 15900 | 150.00 | 1.0 |
| Bepridil | 160.0 | 1000 | 2300 | 35.00 | 1.0 |
| Sotalol | 111400.0 | 193300 | 7.0139e+06 | 13000.00 | 1.0 |
| Diltiazem | 13200.0 | 760 | 22400 | 180.00 | 0.0 |
| Disopyramide | 14400.0 | 1.0367e+06 | 168400 | 750.00 | 1.0 |
| Dofetilide | 30.0 | 26700 | 162100 | 2.00 | 1.0 |
| Dronedarone | 59.0 | 400 | 700 | 1.80 | 1.0 |
| E4031 | 14.0 | - | - | 8.40 | 1.0 |
| Flecainide | 1500.0 | 27100 | 6200 | 1200.00 | 1.0 |
| Ibutilide | 18.0 | 62500 | 42500 | 150.00 | 1.0 |
| Metoprolol | 145000.0 | - | 40000 | 450.00 | 0.0 |
| Mexiletine | 62200.0 | 125000 | 38000 | 5400.00 | 0.0 |
| Mibefradil | 1700.0 | 510 | 5600 | 63.00 | 0.0 |
| Nifedipine | 44000.0 | 12 | 88500 | 10.00 | 0.0 |
| Nitrendipine | 24600.0 | 25 | 21600 | 3.00 | 0.0 |
| Prenylamine | 65.0 | 1200 | 2500 | 3.50 | 1.0 |
| Procainamide | 272400.0 | 389500 | 746600 | 52000.00 | 1.0 |
| Quinidine | 720.0 | 6400 | 14600 | 3200.00 | 1.0 |
| Ranolazine | 11500.0 | 296000 | 294000 | 2300.00 | 3.0 |
| Sematilide | 25000.0 | - | - | 4400.00 | 1.0 |
| Verapamil | 250.0 | 200 | 32500 | 92.00 | 0.0 |
| Astemizole | 4.0 | 1100 | 3000 | 0.51 | 1.0 |
| Chlorpheniramine | 13000.0 | - | - | 12.00 | 0.0 |
| Diphenhydramine | 5200.0 | 228000 | 41000 | 57.00 | 3.0 |
| Levocetirizine | 100000.0 | - | - | 63.00 | 0.0 |
| Loratadine | 6100.0 | 11400 | 28900 | 0.60 | 0.0 |
| Terfenadine | 50.0 | 930 | 2000 | 9.50 | 1.0 |
| Famotidine | 10000.0 | - | - | 330.00 | 2.0 |
| Cisapride | 20.0 | 11800 | 337000 | 2.60 | 1.0 |
| Dolasetron | 5950.0 | - | 38000 | 360.00 | 2.0 |
| Domperidone | 160.0 | - | 5600 | 27.00 | 1.0 |
| Ondansetron | 1500.0 | 22600 | 20000 | 77.00 | 1.0 |
| Terodiline | 650.0 | 4800 | 7400 | 240.00 | 1.0 |
| Tolterodine | 17.0 | 143 | 1000 | 0.14 | 2.0 |
| Aspirin | 100000000.0 | - | - | 7700.00 | 0.0 |
| Cilostazol | 13800.0 | 91200 | 93700 | 140.00 | 1.0 |
| Amitriptyline | 5800.0 | 11600 | 1600 | 41.00 | 3.0 |
| Chlorpromazine | 1500.0 | 3400 | 3000 | 38.00 | 1.0 |
| Clozapine | 2300.0 | 3600 | 15100 | 320.00 | 2.0 |
| Droperidol | 60.0 | 7600 | 22700 | 21.00 | 1.0 |
| Haloperidol | 40.0 | 1300 | 4300 | 3.60 | 1.0 |
| Paliperidone | 780.0 | 193900 | 109000 | 30.00 | 2.0 |
| Pimozide | 40.0 | 240 | 1100 | 0.43 | 1.0 |
| Risperidone | 260.0 | 34200 | 43400 | 15.00 | 2.0 |
| Sertindole | 33.0 | 6300 | 6900 | 1.60 | 2.0 |
| Thioridazine | 500.0 | 3500 | 1400 | 45.00 | 1.0 |
| Ziprasidone | 240.0 | - | 170000 | 7.30 | 3.0 |
| Clarithromycin | 32900.0 | - | - | 3300.00 | 1.0 |
| Erythromycin | 38900.0 | - | - | 12000.00 | 1.0 |
| Gatifloxacin | 130000.0 | - | - | 9000.00 | 1.0 |
| Moxifloxacin | 86200.0 | 173000 | 1.112e+06 | 5600.00 | 1.0 |
| Sparfloxacin | 22100.0 | 88800 | 2.555e+06 | 1900.00 | 1.0 |
| Quinine | 42200.0 | 14000 | 17800 | 4300.00 | 3.0 |
| Nilotinib | 1000.0 | 17500 | 13300 | 170.00 | 2.0 |
| Tamoxifen | 777.0 | - | - | 18.00 | 2.0 |
| Vandetanib | 400.0 | - | - | 110.00 | 1.0 |

**Table S6.** $IC_{50}$, Hill coefficients, EFTPC values, drug trapping parameter and risk categorization for the (Ando et al., 2017) dataset. Target column represents the risk categorization according to crediblemeds, 0: Drugs not present in the CredibleMeds databse. 1: Drugs with known risk (CM1) 2: Drugs with possible risk(CM2) 3: Drugs with conditional risk (CM3). Binarized definition assigned in Ando. et al., TdP+: 1 TdP-: 0,2,3

**Dataset 7: Li et al. 2017**. A subset consisting of 12 of the 30 drugs in Dataset 5, categorized into low (CP3), intermediate (CP2) and high risk (CP1) categories (Fermini et al., 2016; Colatsky et al., 2016), were analyzed using the OHR model that was modified to include dynamics drug-hERG channel interactions.$IC_{50}$ for the hERG channels were measured while the $IC_{50}$ for other channels were taken from the (Crumb et al., 2016). The summation of the ratio of the area under the curve of calcium and late sodium current traces obtained at steady state action potential simulation in the presence to the absence of the drugs (i.e., $\frac{AUCI_{NaL,drug}}{AUCI_{NaL,control}} + \frac{AUCI_{CaV,drug}}{AUCI_{CaV,control}}$). In this study, we developed our classifier using the identical ternary categories as in (Li et al., 2017) as well as binary TdP definitions. For binary TdP definition, we merged high and intermediate risk drugs (CP1 and CP2 categories) to represent drugs that are torsadogenic (TdP+). Drugs with low TdP risk (CP3 category) were considered to be non-torsadogenic (TdP-).

| | $IC_{50,I_{Kr}}$ | $IC_{50,I_{CaV}}$ | $IC_{50,I_{Na,peak}}$ | $IC_{50,I_{Ks}}$ | $IC_{50,I_{K1}}$ | $IC_{50,I_{to}}$ | $IC_{50,I_{Na,late}}$ | $h_{I_{Kr}}$ | $h_{I_{CaV}}$ | $h_{I_{Na,peak}}$ | $h_{I_{Ks}}$ | $h_{I_{K1}}$ | $h_{I_{to}}$ | $h_{I_{NaL}}$ | $EFTPC$ | $Target$ | Vtrap |
|---|---|---|---|---|---|---|---|---|---|---|---|---|---|---|---|---|---|
| Bepridil | 50.0 | 2808 | 2929.3 | 28628.3 | - | 8594 | 1814 | 0.9 | 0.6 | 1.2 | 0.7 | 1.0 | 3.5 | 1.4 | 33.0 | 2.0 | -54.93 |
| Chlorpromazine | 929.2 | 8192 | 4535 | - | 9269.9 | 1.76167e+07 | 4560 | 0.8 | 0.8 | 2.0 | 1.0 | 0.7 | 0.4 | 0.9 | 38.0 | 1.0 | -14.00 |
| Cisapride | 10.0 | 9.25808e+06 | 1e+10 | 8.11929e+07 | 29498 | 219112 | - | 0.7 | 0.4 | 1.0 | 0.3 | 0.5 | 0.2 | 1.0 | 2.6 | 1.0 | -199.00 |
| Diltiazem | 13150.0 | 112 | 110859 | - | - | 2.8e+09 | 21868.5 | 0.9 | 0.7 | 0.7 | 1.0 | 1.0 | 0.2 | 0.7 | 122.0 | 0.0 | -90.00 |
| Dofetilide | 4.9 | 260.3 | 380.5 | - | 394.3 | 18.8 | 753160 | 0.9 | 1.2 | 0.9 | 1.0 | 0.8 | 0.8 | 0.3 | 2.0 | 2.0 | -1.00 |
| Mexiletine | 28880.0 | 38243.6 | - | - | - | - | 8956.8 | 0.9 | 1.0 | 1.0 | 1.0 | 1.0 | 1.0 | 1.4 | 4129.0 | 0.0 | -86.00 |
| Ondansetron | 1320.0 | 22551 | 57666.4 | 569807 | - | 1.02338e+06 | 19181 | 0.9 | 0.8 | 1.0 | 0.7 | 1.0 | 1.0 | 1.0 | 139.0 | 1.0 | -82.00 |
| Quinidine | 992.0 | 51592.3 | 12329 | 4899 | 3.95899e+07 | 3487.4 | 9417 | 0.8 | 0.6 | 1.5 | 1.4 | 0.4 | 1.3 | 1.3 | 3237.0 | 2.0 | -64.70 |
| Ranolazine | 8270.0 | - | 68774 | 3.6155e+07 | - | - | 7884 | 0.9 | 1.0 | 1.4 | 0.5 | 1.0 | 1.0 | 0.9 | 1948.2 | 0.0 | -94.00 |
| Sotalol | 110600.0 | 7.06153e+06 | 1.14e+09 | 4.22186e+06 | 3.05026e+06 | 4.31435e+07 | - | 0.8 | 0.9 | 0.5 | 1.2 | 1.2 | 0.7 | 1.0 | 14690.0 | 2.0 | -55.00 |
| Terfenadine | 23.0 | 700 | 4803.2 | 399754 | - | 239961 | 20056 | 0.6 | 0.7 | 1.0 | 0.5 | 1.0 | 0.3 | 0.6 | 4.0 | 1.0 | -77.00 |
| Verapamil | 288.0 | 201 | - | - | 3.49e+08 | 13429.2 | 7028 | 1.0 | 1.1 | 1.0 | 1.0 | 0.3 | 0.8 | 1.0 | 81.0 | 0.0 | -100.00 |

**Table S7.** $IC_{50}$, Hill coefficients, EFTPC values, drug trapping parameters and risk categorization for the (Li et al., 2017) dataset. Target column represents the tertiary risk categorization assigned in Li et al., 0: Low Risk(CP3) 1: Intedmediate Risk(CP2) 2: High Risk (CP1). Binary risk categorization used for this study, TdP+: 1 and 2, TdP-: 0.

**Dataset 8: Merged Dataset**. In this study we also combine Datasets 1, 2, 3, 5, 6, and 7 to form a single merged dataset. Drug-induced block of different channels at drug's EFTPC and at drug concentrations equal to hERG $IC_{60}$ for the merged dataset is reported in the Table S8 and the Table S9. The merged dataset comprises of 197 drugs of which 95 were unique. Several drugs were common across more than one datasets. The *in-vitro* block values of the channels and maximum EFTPCs were different for different datasets; we analyzed the common drugs across the datasets as distinct compounds to build/test the classifiers. For the merged dataset, we carried out simulation for two different conditions. First, considering the block of hERG and $CaV$ channels. Second, considering the block of all the available channels for the particular drug. Taking into account the block of other ion-channels in addition to hERG and $CaV$ for TdP risk classification did not result in significant improvement in the classification accuracies.

| | $EFTPC$ | $Source$ | $I_{CaV}$ | $I_{K1}$ | $I_{Kr}$ | $I_{Ks}$ | $I_{NaL}$ | $I_{Na}$ | $Ratio$ | $target$ | $I_{to}$ | $vtrap$ | $CM$ | $t1$ | $t2$ | $t3$ |
|---|---|---|---|---|---|---|---|---|---|---|---|---|---|---|---|---|
| Ajmaline | 1500.00 | Mirams et al. | 2.07 | - | 59.06 | - | - | 15.46 | 0.69 | 1 | - | - | 0 | 0 | 0 | 0 |
| Ajmaline | 25.00 | Ando et al. | 0.04 | - | 2.35 | - | - | 0.30 | 41.60 | 1 | - | - | 0 | 0 | 0 | 0 |
| Amiodarone | 150.00 | Ando et al. | 7.32 | - | 14.85 | - | - | 0.93 | 5.73 | 1 | - | - | 1 | 1 | 1 | 1 |
| Amiodarone | 0.77 | Okada et al. | 0.06 | 0 | 0.36 | 0 | 0 | 0.48 | 975.47 | 1 | 0 | - | 1 | 1 | 1 | 1 |
| Amiodarone | 0.80 | Kramer et al. | 0.47 | - | 0.05 | - | - | 0.01 | 1075.00 | 1 | - | - | 1 | 1 | 1 | 1 |
| Amiodarone | 0.70 | Crumb et al. | 1.09 | 0 | 1.31 | 0.01 | 2.18 | 0.21 | 1344.29 | 1 | 3.12 | - | 1 | 1 | 1 | 1 |
| Amiodarone | 0.50 | Mirams et al. | 0.18 | - | 1.64 | - | - | 0.01 | 60.00 | 1 | - | - | 1 | 1 | 1 | 1 |
| Amitriptyline | 36.40 | Crumb et al. | 2.74 | 0 | 0.98 | 10.34 | 8.31 | 0.14 | 100.55 | 0 | 15.46 | - | 3 | 0 | 0 | 1 |
| Amitriptyline | 41.00 | Mirams et al. | 0.35 | - | 1.23 | - | - | 0.20 | 80.00 | 0 | - | - | 3 | 0 | 0 | 1 |
| Amitriptyline | 41.00 | Ando et al. | 0.35 | - | 0.70 | - | - | 2.50 | 141.46 | 0 | - | - | 3 | 0 | 0 | 1 |
| Aspirin | 7700.00 | Ando et al. | 0.00 | - | 0.01 | - | - | 0.00 | 12987.01 | 0 | - | - | 0 | 0 | 0 | 0 |
| Astemizole | 0.30 | Kramer et al. | 0.00 | - | 11.71 | - | - | 0.00 | 13.33 | 1 | - | - | 1 | 1 | 1 | 1 |
| Astemizole | 0.29 | Okada et al. | 0.00 | 0 | 0.03 | 0 | 0 | 0.00 | 97.71 | 1 | 0 | - | 1 | 1 | 1 | 1 |
| Astemizole | 0.51 | Ando et al. | 0.05 | - | 11.31 | - | - | 0.02 | 7.84 | 1 | - | - | 1 | 1 | 1 | 1 |
| Azithromycin | 1937.00 | Crumb et al. | 0.00 | 0 | 14.19 | 0.05 | 0.02 | 0.00 | 36.55 | 1 | 12.87 | - | 1 | 1 | 1 | 1 |
| Bepridil | 31.50 | Crumb et al. | 6.33 | 0 | 19.80 | 0.84 | 0.34 | 0.43 | 4.73 | 1 | 0 | - | 1 | 1 | 1 | 1 |
| Bepridil | 33.00 | Mirams et al. | 13.52 | - | 50.00 | - | - | 0.88 | 1.00 | 1 | - | - | 1 | 1 | 1 | 1 |
| Bepridil | 35.00 | Ando et al. | 3.38 | - | 17.95 | - | - | 1.50 | 4.57 | 1 | - | - | 1 | 1 | 1 | 1 |
| Bepridil | 35.00 | Kramer et al. | 1.35 | - | 20.79 | - | - | 0.51 | 4.57 | 1 | - | - | 1 | 1 | 1 | 1 |
| Bepridil | 33.00 | Li et al. | 6.50 | 0 | 40.76 | 0.87 | 0.36 | 0.46 | 1.52 | 1 | 0 | -54.93 | 1 | 1 | 1 | 1 |
| Bepridil | 34.63 | Okada et al. | 0.02 | 0 | 12.62 | 0.01 | 0 | 3.29 | 3.76 | 1 | 0.01 | - | 1 | 1 | 1 | 1 |
| Ceftriaxone | 23170.00 | Kramer et al. | 13.09 | - | 4.94 | - | - | 4.00 | 19.24 | 0 | - | - | 0 | 0 | 0 | 0 |
| Chloroquine | 249.50 | Crumb et al. | 0.00 | 4.75 | 12.02 | 0 | 0.36 | 0.31 | 27.61 | 1 | 0 | - | 1 | 1 | 1 | 1 |
| Chlorpheniramine | 12.00 | Ando et al. | 0.00 | - | 0.09 | - | - | 0.00 | 1083.33 | 1 | - | - | 0 | 0 | 0 | 0 |
| Chlorpromazine | 38.00 | Ando et al. | 1.11 | - | 2.47 | - | - | 1.25 | 39.47 | 1 | - | - | 1 | 1 | 1 | 1 |
| Chlorpromazine | 38.00 | Mirams et al. | 0.00 | - | 2.52 | - | - | 0.88 | 38.68 | 1 | - | - | 1 | 1 | 1 | 1 |
| Chlorpromazine | 38.00 | Kramer et al. | 0.04 | - | 0.58 | - | - | 0.68 | 39.47 | 1 | - | - | 1 | 1 | 1 | 1 |
| Chlorpromazine | 34.50 | Crumb et al. | 1.24 | 1.95 | 4.19 | 0 | 1.22 | 0.01 | 32.41 | 1 | 0.52 | - | 1 | 1 | 1 | 1 |
| Chlorpromazine | 38.00 | Li et al. | 1.34 | 2.09 | 7.19 | 0 | 1.33 | 0.01 | 24.45 | 1 | 0.54 | -14 | 1 | 1 | 1 | 1 |
| Cibenzoline | 673.00 | Crumb et al. | 0.00 | 0 | 26.45 | 0 | 7.29 | 1.52 | 3.12 | 0 | 0 | - | 0 | 0 | 0 | 0 |
| Cibenzoline | 976.00 | Mirams et al. | 3.15 | - | 4.14 | - | - | 11.12 | 23.16 | 0 | - | - | 0 | 0 | 0 | 0 |
| Cilostazol | 140.00 | Ando et al. | 0.15 | - | 1.00 | - | - | 0.15 | 98.57 | 1 | - | - | 1 | 1 | 1 | 1 |
| Cilostazol | 128.00 | Kramer et al. | 0.14 | - | 1.39 | - | - | 0.14 | 107.81 | 1 | - | - | 1 | 1 | 1 | 1 |
| Cisapride | 2.60 | Crumb et al. | 0.24 | 0.93 | 12.04 | 0.56 | 0 | 0.00 | 4.62 | 1 | 9.38 | - | 1 | 1 | 1 | 1 |
| Cisapride | 2.60 | Ando et al. | 0.02 | - | 11.50 | - | - | 0.00 | 7.69 | 1 | - | - | 1 | 1 | 1 | 1 |
| Cisapride | 2.60 | Li et al. | 0.24 | 0.93 | 28.03 | 0.56 | 0 | 0.00 | 3.85 | 1 | 9.38 | -199 | 1 | 1 | 1 | 1 |
| Cisapride | 2.58 | Okada et al. | 0.00 | 0 | 9.03 | 0 | 0 | 0.25 | 5.71 | 1 | 0 | - | 1 | 1 | 1 | 1 |
| Cisapride | 4.90 | Mirams et al. | 0.00 | - | 42.98 | - | - | 0.03 | 1.33 | 1 | - | - | 1 | 1 | 1 | 1 |
| Cisapride | 3.00 | Kramer et al. | 0.03 | - | 12.21 | - | - | 0.00 | 6.67 | 1 | - | - | 1 | 1 | 1 | 1 |
| Clarithromycin | 3300.00 | Ando et al. | 0.00 | - | 9.12 | - | - | 0.00 | 9.97 | 1 | - | - | 1 | 1 | 1 | 1 |
| Clozapine | 71.00 | Kramer et al. | 1.93 | - | 3.31 | - | - | 0.22 | 32.39 | 1 | - | - | 2 | 1 | 0 | 0 |
| Clozapine | 320.00 | Ando et al. | 8.16 | - | 12.21 | - | - | 2.08 | 7.19 | 1 | - | - | 2 | 1 | 0 | 0 |
| Desipramine | 108.00 | Mirams et al. | 5.94 | - | 7.21 | - | - | 6.63 | 12.87 | 0 | - | - | 2 | 1 | 0 | 0 |
| Diazepam | 29.00 | Kramer et al. | 0.20 | - | 0.03 | - | - | 0.01 | 1834.48 | 0 | - | - | 0 | 0 | 0 | 0 |
| Diltiazem | 122.00 | Mirams et al. | 21.33 | - | 0.70 | - | - | 1.34 | 141.80 | 0 | - | - | 0 | 0 | 0 | 0 |
| Diltiazem | 122.00 | Li et al. | 51.50 | 0 | 1.46 | 0 | 2.58 | 0.84 | 107.79 | 0 | 3.26 | -90 | 0 | 0 | 0 | 0 |
| Diltiazem | 127.50 | Crumb et al. | 52.27 | 0 | 4.09 | 0 | 2.66 | 0.87 | 51.52 | 0 | 3.29 | - | 0 | 0 | 0 | 0 |
| Diltiazem | 180.00 | Ando et al. | 19.15 | - | 1.35 | - | - | 0.80 | 73.33 | 0 | - | - | 0 | 0 | 0 | 0 |
| Diltiazem | 122.00 | Kramer et al. | 11.05 | - | 0.43 | - | - | 0.12 | 108.20 | 0 | - | - | 0 | 0 | 0 | 0 |
| Diphenhydramine | 57.00 | Ando et al. | 0.02 | - | 1.08 | - | - | 0.14 | 91.23 | 0 | - | - | 3 | 0 | 0 | 1 |
| Diphenhydramine | 34.00 | Mirams et al. | 0.01 | - | 0.65 | - | - | 0.08 | 152.94 | 0 | - | - | 3 | 0 | 0 | 1 |
| Disopyramide | 750.00 | Ando et al. | 0.07 | - | 4.95 | - | - | 0.44 | 19.20 | 1 | - | - | 1 | 1 | 1 | 1 |
| Disopyramide | 742.00 | Kramer et al. | 0.07 | - | 6.30 | - | - | 0.27 | 19.41 | 1 | - | - | 1 | 1 | 1 | 1 |

| | | | | | | | | | | | | | | | | | |
|---|---|---|---|---|---|---|---|---|---|---|---|---|---|---|---|---|---|
| Dofetilide | 1.60 | Okada et al. | 0.00 | 0 | 0.18 | 0 | 0 | 2.40 | 23.92 | 1 | 0 | - | 1 | 1 | 1 | 1 |
| Dofetilide | 2.00 | Ando et al. | 0.01 | - | 6.25 | - | - | 0.00 | 15.00 | 1 | - | - | 1 | 1 | 1 | 1 |
| Dofetilide | 2.10 | Crumb et al. | 0.80 | 1.49 | 60.95 | 0 | 0 | 0.00 | 0.48 | 1 | 14.76 | - | 1 | 1 | 1 | 1 |
| Dofetilide | 2.00 | Li et al. | 0.29 | 1.44 | 30.86 | 0 | 2.08 | 0.88 | 2.45 | 1 | 14.28 | -1 | 1 | 1 | 1 | 1 |
| Dofetilide | 2.00 | Mirams et al. | 0.00 | - | 28.57 | - | - | 0.00 | 2.50 | 1 | - | - | 1 | 1 | 1 | 1 |
| Dofetilide | 2.00 | Kramer et al. | 0.01 | - | 3.73 | - | - | 0.00 | 15.00 | 1 | - | - | 1 | 1 | 1 | 1 |
| Dolasetron | 360.00 | Ando et al. | 0.00 | - | 5.71 | - | - | 0.94 | 16.53 | 1 | - | - | 2 | 1 | 0 | 0 |
| Domperidone | 27.00 | Ando et al. | 0.00 | - | 14.44 | - | - | 0.48 | 5.93 | 1 | - | - | 1 | 1 | 1 | 1 |
| Donepezil | 3.00 | Kramer et al. | 0.04 | - | 0.48 | - | - | 0.01 | 233.33 | 1 | - | - | 1 | 1 | 1 | 1 |
| Dosatinib | 41.00 | Kramer et al. | 0.05 | - | 0.06 | - | - | 0.00 | 597.56 | 0 | - | - | 2 | 1 | 0 | 0 |
| Dronedarone | 1.80 | Ando et al. | 0.45 | - | 2.96 | - | - | 0.26 | 32.78 | 1 | - | - | 1 | 1 | 1 | 1 |
| Droperidol | 16.00 | Kramer et al. | 0.08 | - | 18.94 | - | - | 0.01 | 3.75 | 1 | - | - | 1 | 1 | 1 | 1 |
| Droperidol | 21.00 | Ando et al. | 0.28 | - | 25.93 | - | - | 0.09 | 2.86 | 1 | - | - | 1 | 1 | 1 | 1 |
| Duloxetine | 16.00 | Kramer et al. | 0.07 | - | 0.05 | - | - | 0.01 | 237.50 | 0 | - | - | 0 | 0 | 0 | 0 |
| E4031 | 5.67 | Okada et al. | 0.48 | 0 | 4.47 | 0 | 0 | 1.93 | 4.63 | 1 | 0 | - | 0 | 0 | 0 | 0 |
| E4031 | 8.40 | Ando et al. | 0.00 | - | 37.50 | - | - | 0.00 | 1.67 | 1 | - | - | 0 | 0 | 0 | 0 |
| Erythromycin | 12000.00 | Ando et al. | 0.00 | - | 23.58 | - | - | 0.00 | 3.24 | 1 | - | - | 1 | 1 | 1 | 1 |
| Famotidine | 330.00 | Ando et al. | 0.00 | - | 3.19 | - | - | 0.00 | 30.30 | 0 | - | - | 2 | 1 | 0 | 0 |
| Flecainide | 753.00 | Kramer et al. | 3.00 | - | 35.29 | - | - | 8.29 | 1.99 | 1 | - | - | 1 | 1 | 1 | 1 |
| Flecainide | 1200.00 | Ando et al. | 4.24 | - | 44.44 | - | - | 16.22 | 1.25 | 1 | - | - | 1 | 1 | 1 | 1 |
| Flecainide | 752.90 | Crumb et al. | 0.71 | 0 | 51.69 | 0 | 12.64 | 1.56 | 0.92 | 1 | 14.72 | - | 1 | 1 | 1 | 1 |
| Fluvoxamine | 377.00 | Mirams et al. | 7.14 | - | 10.84 | - | - | 0.95 | 8.22 | 0 | - | - | 3 | 0 | 0 | 1 |
| Gatifloxacin | 9000.00 | Ando et al. | 0.00 | - | 6.47 | - | - | 0.00 | 14.44 | 1 | - | - | 1 | 1 | 1 | 1 |
| Halofantrine | 172.00 | Kramer et al. | 8.49 | - | 26.15 | - | - | 0.05 | 2.21 | 1 | - | - | 1 | 1 | 1 | 1 |
| Haloperidol | 4.00 | Kramer et al. | 0.03 | - | 6.20 | - | - | 0.00 | 10.00 | 1 | - | - | 1 | 1 | 1 | 1 |
| Haloperidol | 3.60 | Mirams et al. | 0.21 | - | 11.76 | - | - | 0.05 | 7.50 | 1 | - | - | 1 | 1 | 1 | 1 |
| Haloperidol | 3.60 | Ando et al. | 0.28 | - | 8.26 | - | - | 0.08 | 11.11 | 1 | - | - | 1 | 1 | 1 | 1 |
| Ibutilide | 150.00 | Ando et al. | 0.24 | - | 89.29 | - | - | 0.35 | 0.12 | 1 | - | - | 1 | 1 | 1 | 1 |
| Ibutilide | 140.00 | Kramer et al. | 0.08 | - | 95.85 | - | - | 0.28 | 0.13 | 1 | - | - | 1 | 1 | 1 | 1 |
| Imipramine | 106.00 | Mirams et al. | 1.26 | - | 3.02 | - | - | 2.86 | 32.08 | 0 | - | - | 2 | 1 | 0 | 0 |
| Lamivudine | 19540.00 | Kramer et al. | 28.74 | - | 0.94 | - | - | 1.23 | 105.12 | 0 | - | - | 0 | 0 | 0 | 0 |
| Levocetirizine | 63.00 | Ando et al. | 0.00 | - | 0.06 | - | - | 0.00 | 1587.30 | 0 | - | - | 0 | 0 | 0 | 0 |
| Lidocaine | 2560.00 | Crumb et al. | 0.00 | 0 | 0.00 | 0 | 13.35 | 0.00 | 39062500.00 | 0 | 0 | - | 0 | 0 | 0 | 0 |
| Linezolid | 59110.00 | Kramer et al. | 36.73 | - | 4.90 | - | - | 2.19 | 19.41 | 0 | - | - | 0 | 0 | 0 | 0 |
| Lopinavir | 703.70 | Crumb et al. | 4.32 | 0 | 8.37 | 0 | 0 | 0.00 | 7.35 | 1 | 0 | - | 0 | 0 | 0 | 0 |
| Loratadine | 0.60 | Ando et al. | 0.01 | - | 0.01 | - | - | 0.00 | 10166.67 | 0 | - | - | 0 | 0 | 0 | 0 |
| Loratadine | 0.40 | Kramer et al. | 0.00 | - | 0.00 | - | - | 0.00 | 15250.00 | 0 | - | - | 0 | 0 | 0 | 0 |
| Methadone | 507.00 | Kramer et al. | 0.08 | - | 12.65 | - | - | 0.34 | 6.90 | 1 | - | - | 1 | 1 | 1 | 1 |
| Metoprolol | 450.00 | Ando et al. | 0.00 | - | 0.31 | - | - | 1.11 | 322.22 | 0 | - | - | 0 | 0 | 0 | 0 |
| Metronidazole | 187000.00 | Kramer et al. | 50.82 | - | 12.24 | - | - | 8.27 | 7.17 | 0 | - | - | 0 | 0 | 0 | 0 |
| Mexiletine | 2503.20 | Crumb et al. | 5.89 | 0 | 3.03 | 0 | 14.37 | 0.00 | 31.96 | 0 | 0 | - | 0 | 0 | 0 | 0 |
| Mexiletine | 5400.00 | Ando et al. | 4.14 | - | 7.99 | - | - | 12.44 | 11.52 | 0 | - | - | 0 | 0 | 0 | 0 |
| Mexiletine | 4129.00 | Mirams et al. | 3.97 | - | 7.63 | - | - | 8.76 | 12.11 | 0 | - | - | 0 | 0 | 0 | 0 |
| Mexiletine | 4129.00 | Li et al. | 9.74 | 0 | 14.80 | 0 | 25.27 | 0.00 | 6.99 | 0 | 0 | -86 | 0 | 0 | 0 | 0 |
| Mibefradil | 63.00 | Ando et al. | 10.99 | - | 3.57 | - | - | 1.11 | 26.98 | 0 | - | - | 0 | 0 | 0 | 0 |
| Mibefradil | 12.00 | Mirams et al. | 7.14 | - | 0.66 | - | - | 1.21 | 150.00 | 0 | - | - | 0 | 0 | 0 | 0 |
| Mibefradil | 10.60 | Crumb et al. | 1.07 | 0 | 4.61 | 0 | 0.09 | 0.18 | 28.96 | 0 | 0 | - | 0 | 0 | 0 | 0 |
| Mibefradil | 12.00 | Kramer et al. | 0.45 | - | 0.11 | - | - | 0.01 | 141.67 | 0 | - | - | 0 | 0 | 0 | 0 |
| Mitoxantrone | 225.00 | Kramer et al. | 4.99 | - | 0.04 | - | - | 0.18 | 2397.33 | 0 | - | - | 0 | 0 | 0 | 0 |
| Moxifloxacin | 10950.00 | Okada et al. | 0.30 | 0 | 9.19 | 0.22 | 0 | 0.00 | 12.40 | 1 | 2.26 | - | 1 | 1 | 1 | 1 |
| Moxifloxacin | 3562.50 | Crumb et al. | 0.00 | 0 | 12.37 | 6.61 | 0.58 | 0.00 | 26.12 | 1 | 0 | - | 1 | 1 | 1 | 1 |
| Moxifloxacin | 10960.00 | Kramer et al. | 5.96 | - | 12.58 | - | - | 0.98 | 7.86 | 1 | - | - | 1 | 1 | 1 | 1 |
| Moxifloxacin | 5600.00 | Ando et al. | 3.14 | - | 6.10 | - | - | 0.50 | 15.39 | 1 | - | - | 1 | 1 | 1 | 1 |
| Nifedipine | 8.00 | Kramer et al. | 39.81 | - | 0.10 | - | - | 0.13 | 5500.00 | 0 | - | - | 0 | 0 | 0 | 0 |

| | | | | | | | | | | | | | | | | |
|---|---|---|---|---|---|---|---|---|---|---|---|---|---|---|---|---|
| Nifedipine | 7.70 | Mirams et al. | 11.37 | - | 0.00 | - | - | 0.02 | 35714.29 | 0 | - | - | 0 | 0 | 0 | 0 |
| Nifedipine | 10.00 | Ando et al. | 45.45 | - | 0.02 | - | - | 0.01 | 4400.00 | 0 | - | - | 0 | 0 | 0 | 0 |
| Nilotinib | 60.40 | Crumb et al. | 0.00 | 0 | 41.88 | 0 | 1.27 | 0.00 | 1.51 | 1 | 0 | - | 2 | 1 | 0 | 0 |
| Nilotinib | 172.00 | Kramer et al. | 0.97 | - | 15.58 | - | - | 0.01 | 5.81 | 1 | - | - | 2 | 1 | 0 | 0 |
| Nilotinib | 170.00 | Ando et al. | 0.96 | - | 14.53 | - | - | 1.26 | 5.88 | 1 | - | - | 2 | 1 | 0 | 0 |
| Nitrendipine | 3.02 | Mirams et al. | 89.61 | - | 0.03 | - | - | 0.01 | 3311.26 | 0 | - | - | 0 | 0 | 0 | 0 |
| Nitrendipine | 3.00 | Ando et al. | 10.71 | - | 0.01 | - | - | 0.01 | 8200.00 | 0 | - | - | 0 | 0 | 0 | 0 |
| Nitrendipine | 3.00 | Kramer et al. | 16.06 | - | 0.06 | - | - | 0.00 | 8200.00 | 0 | - | - | 0 | 0 | 0 | 0 |
| Ondansetron | 358.50 | Crumb et al. | 3.51 | 0 | 19.37 | 0.57 | 1.83 | 0.62 | 4.16 | 1 | 0.04 | - | 1 | 1 | 1 | 1 |
| Ondansetron | 77.00 | Ando et al. | 0.34 | - | 4.88 | - | - | 0.38 | 19.48 | 1 | - | - | 1 | 1 | 1 | 1 |
| Ondansetron | 139.00 | Li et al. | 1.68 | 0 | 11.65 | 0.3 | 0.72 | 0.24 | 9.50 | 1 | 0.01 | -82 | 1 | 1 | 1 | 1 |
| Paliperidone | 69.00 | Kramer et al. | 0.04 | - | 7.95 | - | - | 0.01 | 11.30 | 1 | - | - | 2 | 1 | 0 | 0 |
| Paliperidone | 30.00 | Ando et al. | 0.02 | - | 3.70 | - | - | 0.03 | 26.00 | 1 | - | - | 2 | 1 | 0 | 0 |
| Paroxetine | 14.00 | Kramer et al. | 0.04 | - | 0.21 | - | - | 0.02 | 135.71 | 0 | - | - | 3 | 0 | 0 | 1 |
| Pentobarbital | 5171.00 | Kramer et al. | 0.37 | - | 0.36 | - | - | 0.19 | 277.30 | 0 | - | - | 0 | 0 | 0 | 0 |
| Phenytoin | 4360.00 | Kramer et al. | 16.83 | - | 2.88 | - | - | 4.84 | 33.72 | 0 | - | - | 0 | 0 | 0 | 0 |
| Phenytoin | 4500.00 | Mirams et al. | 4.19 | - | 4.31 | - | - | 8.41 | 22.22 | 0 | - | - | 0 | 0 | 0 | 0 |
| Pimozide | 0.50 | Kramer et al. | 0.01 | - | 0.62 | - | - | 0.03 | 80.00 | 1 | - | - | 1 | 1 | 1 | 1 |
| Pimozide | 0.43 | Ando et al. | 0.18 | - | 1.06 | - | - | 0.04 | 93.02 | 1 | - | - | 1 | 1 | 1 | 1 |
| Pimozide | 1.00 | Mirams et al. | 0.61 | - | 4.76 | - | - | 1.82 | 20.00 | 1 | - | - | 1 | 1 | 1 | 1 |
| Piperacillin | 1378000.00 | Kramer et al. | 52.92 | - | 28.81 | - | - | 36.15 | 2.47 | 0 | - | - | 3 | 0 | 0 | 1 |
| Prenylamine | 3.50 | Ando et al. | 0.29 | - | 5.11 | - | - | 0.14 | 18.57 | 1 | - | - | 0 | 0 | 0 | 0 |
| Prenylamine | 17.00 | Mirams et al. | 1.35 | - | 20.73 | - | - | 0.67 | 3.82 | 1 | - | - | 0 | 0 | 0 | 0 |
| Procainamide | 52000.00 | Ando et al. | 11.78 | - | 16.03 | - | - | 6.51 | 5.24 | 1 | - | - | 1 | 1 | 1 | 1 |
| Procainamide | 54180.00 | Kramer et al. | 16.28 | - | 16.59 | - | - | 6.77 | 5.03 | 1 | - | - | 1 | 1 | 1 | 1 |
| Propafenone | 241.00 | Mirams et al. | 11.81 | - | 35.39 | - | - | 16.84 | 1.83 | 1 | - | - | 0 | 0 | 0 | 0 |
| Propafenone | 131.00 | Crumb et al. | 9.76 | 0 | 26.10 | 0 | 4.37 | 3.26 | 3.67 | 1 | 0 | - | 0 | 0 | 0 | 0 |
| Propranolol | 26.00 | Mirams et al. | 0.14 | - | 0.91 | - | - | 1.22 | 108.77 | 0 | - | - | 0 | 0 | 0 | 0 |
| Quetiapine | 33.00 | Mirams et al. | 0.32 | - | 0.57 | - | - | 0.19 | 175.76 | 0 | - | - | 3 | 0 | 0 | 1 |
| Quinidine | 3200.00 | Ando et al. | 33.33 | - | 81.63 | - | - | 17.98 | 0.22 | 1 | - | - | 1 | 1 | 1 | 1 |
| Quinidine | 3237.00 | Li et al. | 15.96 | 2.27 | 72.03 | 35.89 | 19.97 | 11.86 | 0.31 | 1 | 47.58 | -64.7 | 1 | 1 | 1 | 1 |
| Quinidine | 842.90 | Crumb et al. | 1.61 | 1.33 | 71.08 | 7.84 | 8.22 | 1.76 | 0.41 | 1 | 13.64 | - | 1 | 1 | 1 | 1 |
| Quinidine | 3237.00 | Kramer et al. | 38.62 | - | 83.11 | - | - | 13.73 | 0.22 | 1 | - | - | 1 | 1 | 1 | 1 |
| Quinidine | 3235.00 | Okada et al. | 32.80 | 0 | 83.80 | 1.52 | 0 | 6.62 | 0.20 | 1 | 65.33 | - | 1 | 1 | 1 | 1 |
| Quinidine | 3237.00 | Mirams et al. | 17.18 | - | 91.52 | - | - | 16.32 | 0.09 | 1 | - | - | 1 | 1 | 1 | 1 |
| Quinine | 4300.00 | Ando et al. | 23.50 | - | 9.25 | - | - | 19.46 | 9.81 | 1 | - | - | 3 | 0 | 0 | 1 |
| Quinine | 3956.70 | Crumb et al. | 12.71 | 0 | 43.35 | 7.78 | 39.87 | 12.03 | 1.31 | 1 | 4.76 | - | 3 | 0 | 0 | 1 |
| Raltegravir | 7000.00 | Kramer et al. | 2.76 | - | 0.89 | - | - | 0.82 | 111.83 | 0 | - | - | 0 | 0 | 0 | 0 |
| Ranolazine | 2310.00 | Okada et al. | 2.87 | 0 | 40.68 | 0 | 2.54 | 6.90 | 1.70 | 0 | 0.11 | - | 3 | 0 | 0 | 1 |
| Ranolazine | 1948.20 | Li et al. | 0.00 | 0 | 21.40 | 0.73 | 22.13 | 0.68 | 4.24 | 0 | 0 | -94 | 3 | 0 | 0 | 1 |
| Ranolazine | 2300.00 | Ando et al. | 0.77 | - | 16.67 | - | - | 0.78 | 5.00 | 0 | - | - | 3 | 0 | 0 | 1 |
| Ranolazine | 1948.20 | Crumb et al. | 0.00 | 0 | 27.63 | 0.73 | 22.13 | 0.00 | 3.33 | 0 | 0 | - | 3 | 0 | 0 | 1 |
| Ribavirin | 27880.00 | Kramer et al. | 4.29 | - | 2.80 | - | - | 0.92 | 34.68 | 0 | - | - | 0 | 0 | 0 | 0 |
| Risperidone | 2.00 | Kramer et al. | 0.05 | - | 0.80 | - | - | 0.01 | 130.00 | 1 | - | - | 2 | 1 | 0 | 0 |
| Risperidone | 1.81 | Mirams et al. | 0.00 | - | 1.19 | - | - | 0.00 | 82.87 | 1 | - | - | 2 | 1 | 0 | 0 |
| Risperidone | 15.00 | Ando et al. | 0.04 | - | 5.45 | - | - | 0.03 | 17.33 | 1 | - | - | 2 | 1 | 0 | 0 |
| Ritonavir | 436.90 | Crumb et al. | 2.15 | 0 | 7.81 | 0 | 12.36 | 2.51 | 11.80 | 1 | 0 | - | 3 | 0 | 0 | 1 |
| Rufinamide | 83126.90 | Crumb et al. | 26.09 | 0 | 0.00 | 0 | 0 | 0.00 | 1202980.02 | 0 | 0 | - | 0 | 0 | 0 | 0 |
| Saquinavir | 130.00 | Kramer et al. | 4.38 | - | 0.02 | - | - | 0.00 | 130.00 | 1 | - | - | 2 | 1 | 0 | 0 |
| Saquinavir | 417.20 | Crumb et al. | 5.55 | 0 | 10.71 | 0 | 3.23 | 1.28 | 8.33 | 1 | 0 | - | 2 | 1 | 0 | 0 |
| Sematilide | 4400.00 | Ando et al. | 0.00 | - | 14.97 | - | - | 0.00 | 5.68 | 1 | - | - | 0 | 0 | 0 | 0 |
| Sertindole | 1.59 | Mirams et al. | 0.02 | - | 10.20 | - | - | 0.07 | 8.81 | 1 | - | - | 2 | 1 | 0 | 0 |
| Sertindole | 1.60 | Ando et al. | 0.03 | - | 4.62 | - | - | 0.02 | 20.62 | 1 | - | - | 2 | 1 | 0 | 0 |
| Sertindole | 2.00 | Kramer et al. | 0.00 | - | 2.92 | - | - | 0.01 | 16.50 | 1 | - | - | 2 | 1 | 0 | 0 |

| | | | | | | | | | | | | | | | | | | |
|---|---|---|---|---|---|---|---|---|---|---|---|---|---|---|---|---|---|---|
| Sertindole | 1.60 | Crumb et al. | 0.00 | 0 | 17.62 | 0 | 0 | 0.00 | 6.88 | 1 | 0 | - | 2 | 1 | 0 | 0 |
| Sitagliptin | 442.00 | Kramer et al. | 0.30 | - | 0.25 | - | - | 0.04 | 395.25 | 0 | - | - | 0 | 0 | 0 | 0 |
| Solifenacin | 3.00 | Kramer et al. | 0.00 | - | 1.66 | - | - | 0.03 | 93.33 | 1 | - | - | 3 | 0 | 0 | 1 |
| Sotalol | 14680.00 | Okada et al. | 0.00 | 0 | 3.69 | 0 | 0 | 0.00 | 24.28 | 1 | 0 | - | 1 | 1 | 1 | 1 |
| Sotalol | 13000.00 | Ando et al. | 6.30 | - | 10.45 | - | - | 0.18 | 8.57 | 1 | - | - | 1 | 1 | 1 | 1 |
| Sotalol | 14686.40 | Crumb et al. | 0.38 | 0.17 | 16.87 | 0.11 | 0 | 0.36 | 5.88 | 1 | 0.37 | - | 1 | 1 | 1 | 1 |
| Sotalol | 14690.00 | Kramer et al. | 7.06 | - | 18.56 | - | - | 0.21 | 7.58 | 1 | - | - | 1 | 1 | 1 | 1 |
| Sotalol | 14690.00 | Li et al. | 0.38 | 0.17 | 16.59 | 0.11 | 0 | 0.36 | 7.53 | 1 | 0.37 | -55 | 1 | 1 | 1 | 1 |
| Sparfloxacin | 1766.00 | Kramer et al. | 1.95 | - | 8.71 | - | - | 0.07 | 12.51 | 1 | - | - | 1 | 1 | 1 | 1 |
| Sparfloxacin | 1900.00 | Ando et al. | 2.09 | - | 7.92 | - | - | 0.07 | 11.63 | 1 | - | - | 1 | 1 | 1 | 1 |
| Sunitinib | 13.00 | Kramer et al. | 0.02 | - | 1.07 | - | - | 0.02 | 92.31 | 1 | - | - | 2 | 1 | 0 | 0 |
| Tamoxifen | 18.00 | Ando et al. | 0.00 | - | 2.26 | - | - | 0.00 | 43.17 | 0 | - | - | 2 | 1 | 0 | 0 |
| Tedisamil | 85.00 | Mirams et al. | 0.00 | - | 3.29 | - | - | 0.42 | 29.41 | 1 | - | - | 0 | 0 | 0 | 0 |
| Telbivudine | 19720.00 | Kramer et al. | 2.69 | - | 4.46 | - | - | 1.77 | 21.44 | 0 | - | - | 0 | 0 | 0 | 0 |
| Terfenadine | 9.00 | Kramer et al. | 0.02 | - | 10.49 | - | - | 0.01 | 5.56 | 1 | - | - | 1 | 1 | 1 | 1 |
| Terfenadine | 9.00 | Mirams et al. | 2.34 | - | 50.28 | - | - | 0.92 | 0.99 | 1 | - | - | 1 | 1 | 1 | 1 |
| Terfenadine | 9.00 | Okada et al. | 0.01 | 0 | 5.66 | 0 | 0 | 11.39 | 10.95 | 1 | 0 | - | 1 | 1 | 1 | 1 |
| Terfenadine | 4.00 | Li et al. | 2.62 | 0 | 25.93 | 0.32 | 0.6 | 0.08 | 5.75 | 1 | 3.56 | -77 | 1 | 1 | 1 | 1 |
| Terfenadine | 9.50 | Ando et al. | 1.01 | - | 15.97 | - | - | 0.47 | 5.26 | 1 | - | - | 1 | 1 | 1 | 1 |
| Terfenadine | 9.00 | Crumb et al. | 4.53 | 0 | 38.98 | 0.47 | 0.97 | 0.19 | 2.11 | 1 | 4.49 | - | 1 | 1 | 1 | 1 |
| Terodiline | 240.00 | Ando et al. | 4.76 | - | 26.97 | - | - | 3.14 | 2.71 | 1 | - | - | 0 | 0 | 0 | 0 |
| Terodiline | 145.00 | Kramer et al. | 2.83 | - | 17.80 | - | - | 0.79 | 4.48 | 1 | - | - | 0 | 0 | 0 | 0 |
| Thioridazine | 45.00 | Ando et al. | 1.27 | - | 8.26 | - | - | 3.11 | 11.11 | 1 | - | - | 1 | 1 | 1 | 1 |
| Thioridazine | 980.00 | Kramer et al. | 15.21 | - | 65.91 | - | - | 40.23 | 0.51 | 1 | - | - | 1 | 1 | 1 | 1 |
| Thioridazine | 979.00 | Mirams et al. | 42.96 | - | 96.74 | - | - | 34.85 | 0.03 | 1 | - | - | 1 | 1 | 1 | 1 |
| Tolterodine | 0.14 | Ando et al. | 0.10 | - | 0.82 | - | - | 0.01 | 121.43 | 0 | - | - | 2 | 1 | 0 | 0 |
| Toremifene | 26.30 | Crumb et al. | 0.00 | 0 | 0.75 | 0 | 0.65 | 0.00 | 133.08 | 1 | 0 | - | 2 | 1 | 0 | 0 |
| Vandetanib | 110.00 | Ando et al. | 0.00 | - | 21.57 | - | - | 0.00 | 3.64 | 1 | - | - | 1 | 1 | 1 | 1 |
| Verapamil | 45.00 | Crumb et al. | 16.09 | 0.85 | 6.62 | 0 | 0.64 | 0.00 | 11.09 | 0 | 1.04 | - | 0 | 0 | 0 | 0 |
| Verapamil | 81.00 | Mirams et al. | 44.75 | - | 36.16 | - | - | 0.19 | 1.77 | 0 | - | - | 0 | 0 | 0 | 0 |
| Verapamil | 88.00 | Kramer et al. | 34.15 | - | 28.31 | - | - | 0.04 | 2.84 | 0 | - | - | 0 | 0 | 0 | 0 |
| Verapamil | 87.97 | Okada et al. | 17.82 | 0 | 29.71 | 0.43 | 0 | 3.55 | 2.29 | 0 | 2.51 | - | 0 | 0 | 0 | 0 |
| Verapamil | 92.00 | Ando et al. | 31.51 | - | 26.90 | - | - | 0.28 | 2.72 | 0 | - | - | 0 | 0 | 0 | 0 |
| Verapamil | 81.00 | Li et al. | 26.90 | 1.01 | 21.95 | 0 | 1.14 | 0.00 | 3.56 | 0 | 1.65 | -100 | 0 | 0 | 0 | 0 |
| Voriconazole | 7563.00 | Kramer et al. | 1.79 | - | 1.52 | - | - | 0.49 | 64.91 | 0 | - | - | 3 | 0 | 0 | 1 |
| Ziprasidone | 7.30 | Ando et al. | 0.00 | - | 2.95 | - | - | 0.00 | 32.88 | 1 | - | - | 3 | 0 | 0 | 1 |

**Table S8.** Merged Dataset. The table reports the block of seven ion channel currents ($I_{CaV}$, $I_{Kr}$, $I_{K1}$, $I_{Ks}$, $I_{to}$, $I_{Na}$ and $I_{NaL}$) at $C_{Drug}$ = $EFTPC$, the maximum EFTPC value of each of the drugs ("EFTPC" column) , the refrence/source ("Source" column) used to extract the $IC_{50}$, Hill coefficients and EFTPC values for the particular drug, the hERG ratio ("Ratio" column), the drug-trapping parameter values for the available drugs ("vtrap"column) and the different risk definition assigned to them ('target', 'CM', 't1', 't2') columns. target- TdP+:- R1, R2 , R3, CH1, CM1 category or label warning , t1- TdP+: Drugs in CM1 and CM2 category, t2- TdP+: - Drugs in CM1 category, t3- TdP+: - Drugs in CM1 and CM3 category.

65

| | $EFTPC$ | $Source$ | $I_{CaV}$ | $I_{K1}$ | $I_{Kr}$ | $I_{Ks}$ | $I_{NaL}$ | $I_{Na}$ | $Ratio$ | $target$ | $I_{to}$ | $vtrap$ | $CM$ | $t1$ | $t2$ | $t3$ | $EAD$ |
|---|---|---|---|---|---|---|---|---|---|---|---|---|---|---|---|---|---|
| Ajmaline | 1500.00 | Mirams et al. | 2.15 | - | 60.0 | - | - | 15.98 | 1.04 | 1 | - | - | 0 | 0 | 0 | 0 | 1 |
| Ajmaline | 25.00 | Ando et al. | 2.15 | - | 60.0 | - | - | 15.98 | 62.40 | 1 | - | - | 0 | 0 | 0 | 0 | 1 |
| Amiodarone | 150.00 | Ando et al. | 40.44 | - | 60.0 | - | - | 7.50 | 8.60 | 1 | - | - | 1 | 1 | 1 | 1 | 0 |
| Amiodarone | 0.77 | Okada et al. | 23.95 | 0 | 60.0 | 0 | 0 | 54.49 | 600.00 | 1 | 0 | - | 1 | 1 | 1 | 1 | 1 |
| Amiodarone | 0.80 | Kramer et al. | 42.79 | - | 60.0 | - | - | 7.81 | 600.00 | 1 | - | - | 1 | 1 | 1 | 1 | 0 |
| Amiodarone | 0.70 | Crumb et al. | 55.49 | 0 | 60.0 | 24.61 | 34.27 | 34.65 | 600.00 | 1 | 42.96 | - | 1 | 1 | 1 | 1 | 0 |
| Amiodarone | 0.50 | Mirams et al. | 14.29 | - | 60.0 | - | - | 0.93 | 90.00 | 1 | - | - | 1 | 1 | 1 | 1 | 1 |
| Amitriptyline | 36.40 | Crumb et al. | 80.96 | 0 | 60.0 | 58.61 | 52.67 | 48.44 | 150.82 | 0 | 57.64 | - | 3 | 0 | 0 | 1 | 0 |
| Amitriptyline | 41.00 | Mirams et al. | 29.78 | - | 60.0 | - | - | 19.74 | 120.00 | 0 | - | - | 3 | 0 | 0 | 1 | 1 |
| Amitriptyline | 41.00 | Ando et al. | 42.86 | - | 60.0 | - | - | 84.47 | 212.20 | 0 | - | - | 3 | 0 | 0 | 1 | 0 |
| Aspirin | 7700.00 | Ando et al. | 0.00 | - | 60.0 | - | - | 0.00 | 600.00 | 0 | - | - | 0 | 0 | 0 | 0 | 1 |
| Astemizole | 0.30 | Kramer et al. | 0.02 | - | 60.0 | - | - | 0.00 | 22.42 | 1 | - | - | 1 | 1 | 1 | 1 | 1 |
| Astemizole | 0.29 | Okada et al. | 0.02 | 0 | 60.0 | 0 | 0 | 0.23 | 123.26 | 1 | 0 | - | 1 | 1 | 1 | 1 | 1 |
| Astemizole | 0.51 | Ando et al. | 0.54 | - | 60.0 | - | - | 0.20 | 11.76 | 1 | - | - | 1 | 1 | 1 | 1 | 1 |
| Azithromycin | 1937.00 | Crumb et al. | 0.00 | 0 | 60.0 | 18.02 | 41.92 | 0.00 | 82.24 | 1 | 57.26 | - | 1 | 1 | 1 | 1 | 1 |
| Bepridil | 31.50 | Crumb et al. | 18.37 | 0 | 60.0 | 3.34 | 5.37 | 4.59 | 7.42 | 1 | 0 | - | 1 | 1 | 1 | 1 | 1 |
| Bepridil | 33.00 | Mirams et al. | 19.00 | - | 60.0 | - | - | 1.32 | 1.50 | 1 | - | - | 1 | 1 | 1 | 1 | 1 |
| Bepridil | 35.00 | Ando et al. | 19.35 | - | 60.0 | - | - | 9.45 | 6.86 | 1 | - | - | 1 | 1 | 1 | 1 | 1 |
| Bepridil | 35.00 | Kramer et al. | 14.73 | - | 60.0 | - | - | 5.85 | 7.25 | 1 | - | - | 1 | 1 | 1 | 1 | 1 |
| Bepridil | 33.00 | Li et al. | 10.46 | 0 | 60.0 | 1.58 | 1.22 | 1.28 | 2.38 | 1 | 0 | -54.93 | 1 | 1 | 1 | 1 | 1 |
| Bepridil | 34.63 | Okada et al. | 0.70 | 0 | 60.0 | 0.18 | 0 | 17.79 | 4.96 | 1 | 0.23 | - | 1 | 1 | 1 | 1 | 1 |
| Ceftriaxone | 23170.00 | Kramer et al. | 81.30 | - | 60.0 | - | - | 54.60 | 28.85 | 0 | - | - | 0 | 0 | 0 | 0 | 0 |
| Chloroquine | 249.50 | Crumb et al. | 0.00 | 54.89 | 60.0 | 0 | 16.21 | 14.48 | 54.27 | 1 | 0 | - | 1 | 1 | 1 | 1 | 1 |
| Chlorpheniramine | 12.00 | Ando et al. | 0.00 | - | 60.0 | - | - | 0.00 | 600.00 | 1 | - | - | 0 | 0 | 0 | 0 | 1 |
| Chlorpromazine | 38.00 | Ando et al. | 39.82 | - | 60.0 | - | - | 42.86 | 59.21 | 1 | - | - | 1 | 1 | 1 | 1 | 0 |
| Chlorpromazine | 38.00 | Mirams et al. | 0.00 | - | 60.0 | - | - | 33.90 | 58.03 | 1 | - | - | 1 | 1 | 1 | 1 | 1 |
| Chlorpromazine | 38.00 | Kramer et al. | 28.61 | - | 60.0 | - | - | 38.70 | 52.73 | 1 | - | - | 1 | 1 | 1 | 1 | 1 |
| Chlorpromazine | 34.50 | Crumb et al. | 22.57 | 23.77 | 60.0 | 0 | 29.74 | 13.01 | 50.85 | 1 | 2.45 | - | 1 | 1 | 1 | 1 | 1 |
| Chlorpromazine | 38.00 | Li et al. | 20.82 | 22.18 | 60.0 | 0 | 27.38 | 10.37 | 40.59 | 1 | 2.33 | -14 | 1 | 1 | 1 | 1 | 1 |
| Cibenzoline | 673.00 | Crumb et al. | 0.00 | 0 | 60.0 | 0 | 16.94 | 9.39 | 4.89 | 0 | 0 | - | 0 | 0 | 0 | 0 | 1 |
| Cibenzoline | 976.00 | Mirams et al. | 53.05 | - | 60.0 | - | - | 81.29 | 34.73 | 0 | - | - | 0 | 0 | 0 | 0 | 0 |
| Cilostazol | 140.00 | Ando et al. | 18.50 | - | 60.0 | - | - | 18.09 | 147.86 | 1 | - | - | 1 | 1 | 1 | 1 | 1 |
| Cilostazol | 128.00 | Kramer et al. | 19.11 | - | 60.0 | - | - | 18.70 | 168.34 | 1 | - | - | 1 | 1 | 1 | 1 | 1 |
| Cisapride | 2.60 | Crumb et al. | 0.50 | 2.3 | 60.0 | 0.97 | 0 | 0.00 | 6.30 | 1 | 13.01 | - | 1 | 1 | 1 | 1 | 1 |
| Cisapride | 2.60 | Ando et al. | 0.25 | - | 60.0 | - | - | 0.01 | 11.54 | 1 | - | - | 1 | 1 | 1 | 1 | 1 |
| Cisapride | 2.60 | Li et al. | 0.51 | 2.4 | 60.0 | 1 | 0 | 0.00 | 6.86 | 1 | 13.2 | -199 | 1 | 1 | 1 | 1 | 1 |
| Cisapride | 2.58 | Okada et al. | 0.06 | 0 | 60.0 | 0 | 0 | 1.55 | 7.75 | 1 | 0 | - | 1 | 1 | 1 | 1 | 1 |
| Cisapride | 4.90 | Mirams et al. | 0.00 | - | 60.0 | - | - | 0.07 | 1.99 | 1 | - | - | 1 | 1 | 1 | 1 | 1 |
| Cisapride | 3.00 | Kramer et al. | 0.25 | - | 60.0 | - | - | 0.01 | 9.85 | 1 | - | - | 1 | 1 | 1 | 1 | 1 |
| Clarithromycin | 3300.00 | Ando et al. | 0.00 | - | 60.0 | - | - | 0.00 | 14.95 | 1 | - | - | 1 | 1 | 1 | 1 | 1 |
| Clozapine | 71.00 | Kramer et al. | 49.25 | - | 60.0 | - | - | 15.86 | 49.20 | 1 | - | - | 2 | 1 | 0 | 0 | 0 |
| Clozapine | 320.00 | Ando et al. | 48.94 | - | 60.0 | - | - | 18.60 | 10.78 | 1 | - | - | 2 | 1 | 0 | 0 | 0 |
| Desipramine | 108.00 | Mirams et al. | 54.96 | - | 60.0 | - | - | 57.84 | 19.31 | 0 | - | - | 2 | 1 | 0 | 0 | 0 |
| Diazepam | 29.00 | Kramer et al. | 69.69 | - | 60.0 | - | - | 20.23 | 600.00 | 0 | - | - | 0 | 0 | 0 | 0 | 0 |
| Diltiazem | 122.00 | Mirams et al. | 98.30 | - | 60.0 | - | - | 74.25 | 212.70 | 0 | - | - | 0 | 0 | 0 | 0 | 0 |
| Diltiazem | 122.00 | Li et al. | 97.47 | 0 | 60.0 | 0 | 48.98 | 23.56 | 169.13 | 0 | 8.6 | -90 | 0 | 0 | 0 | 0 | 0 |
| Diltiazem | 127.50 | Crumb et al. | 96.10 | 0 | 60.0 | 0 | 38.06 | 16.47 | 85.53 | 0 | 7.65 | - | 0 | 0 | 0 | 0 | 0 |
| Diltiazem | 180.00 | Ando et al. | 96.30 | - | 60.0 | - | - | 46.92 | 110.00 | 0 | - | - | 0 | 0 | 0 | 0 | 0 |
| Diltiazem | 122.00 | Kramer et al. | 97.47 | - | 60.0 | - | - | 44.24 | 153.47 | 0 | - | - | 0 | 0 | 0 | 0 | 0 |
| Diphenhydramine | 57.00 | Ando et al. | 3.31 | - | 60.0 | - | - | 15.98 | 136.84 | 0 | - | - | 3 | 0 | 0 | 1 | 1 |
| Diphenhydramine | 34.00 | Mirams et al. | 3.31 | - | 60.0 | - | - | 15.98 | 229.41 | 0 | - | - | 3 | 0 | 0 | 1 | 1 |
| Disopyramide | 750.00 | Ando et al. | 2.04 | - | 60.0 | - | - | 11.37 | 28.80 | 1 | - | - | 1 | 1 | 1 | 1 | 1 |
| Disopyramide | 742.00 | Kramer et al. | 2.12 | - | 60.0 | - | - | 10.02 | 30.30 | 1 | - | - | 1 | 1 | 1 | 1 | 1 |

| | | | | | | | | | | | | | | | | | |
|---|---|---|---|---|---|---|---|---|---|---|---|---|---|---|---|---|---|
| Dofetilide | 1.60 | Okada et al. | 0.06 | 0 | 60.0 | 0 | 0 | 6.96 | 29.35 | 1 | 0 | - | 1 | 1 | 1 | 1 | 1 |
| Dofetilide | 2.00 | Ando et al. | 0.17 | - | 60.0 | - | - | 0.03 | 22.50 | 1 | - | - | 1 | 1 | 1 | 1 | 1 |
| Dofetilide | 2.10 | Crumb et al. | 0.75 | 1.42 | 60.0 | 0 | 0 | 0.00 | 0.94 | 1 | 14.11 | - | 1 | 1 | 1 | 1 | 1 |
| Dofetilide | 2.00 | Li et al. | 1.44 | 4.11 | 60.0 | 0 | 3.08 | 2.90 | 3.84 | 1 | 32.84 | -1 | 1 | 1 | 1 | 1 | 1 |
| Dofetilide | 2.00 | Mirams et al. | 0.01 | - | 60.0 | - | - | 0.00 | 3.75 | 1 | - | - | 1 | 1 | 1 | 1 | 1 |
| Dofetilide | 2.00 | Kramer et al. | 0.16 | - | 60.0 | - | - | 0.03 | 21.03 | 1 | - | - | 1 | 1 | 1 | 1 | 1 |
| Dolasetron | 360.00 | Ando et al. | 0.00 | - | 60.0 | - | - | 19.02 | 24.79 | 1 | - | - | 2 | 1 | 0 | 0 | 1 |
| Domperidone | 27.00 | Ando et al. | 0.00 | - | 60.0 | - | - | 4.11 | 8.89 | 1 | - | - | 1 | 1 | 1 | 1 | 1 |
| Donepezil | 3.00 | Kramer et al. | 5.28 | - | 60.0 | - | - | 2.68 | 352.91 | 1 | - | - | 1 | 1 | 1 | 1 | 1 |
| Dosatinib | 41.00 | Kramer et al. | 30.00 | - | 60.0 | - | - | 24.52 | 600.00 | 0 | - | - | 2 | 1 | 0 | 0 | 0 |
| Dronedarone | 1.80 | Ando et al. | 18.12 | - | 60.0 | - | - | 11.22 | 49.17 | 1 | - | - | 1 | 1 | 1 | 1 | 1 |
| Droperidol | 16.00 | Kramer et al. | 0.55 | - | 60.0 | - | - | 0.10 | 5.42 | 1 | - | - | 1 | 1 | 1 | 1 | 1 |
| Droperidol | 21.00 | Ando et al. | 1.17 | - | 60.0 | - | - | 0.39 | 4.29 | 1 | - | - | 1 | 1 | 1 | 1 | 1 |
| Duloxetine | 16.00 | Kramer et al. | 69.89 | - | 60.0 | - | - | 49.89 | 317.94 | 0 | - | - | 0 | 0 | 0 | 0 | 0 |
| E4031 | 5.67 | Okada et al. | 1.05 | 0 | 60.0 | 0 | 0 | 3.39 | 5.68 | 1 | 0 | - | 0 | 0 | 0 | 0 | 1 |
| E4031 | 8.40 | Ando et al. | 0.00 | - | 60.0 | - | - | 0.00 | 2.50 | 1 | - | - | 0 | 0 | 0 | 0 | 1 |
| Erythromycin | 12000.00 | Ando et al. | 0.00 | - | 60.0 | - | - | 0.00 | 4.86 | 1 | - | - | 1 | 1 | 1 | 1 | 1 |
| Famotidine | 330.00 | Ando et al. | 0.00 | - | 60.0 | - | - | 0.00 | 45.45 | 0 | - | - | 2 | 1 | 0 | 0 | 1 |
| Flecainide | 753.00 | Kramer et al. | 8.62 | - | 60.0 | - | - | 25.11 | 3.16 | 1 | - | - | 1 | 1 | 1 | 1 | 1 |
| Flecainide | 1200.00 | Ando et al. | 7.67 | - | 60.0 | - | - | 26.63 | 1.88 | 1 | - | - | 1 | 1 | 1 | 1 | 1 |
| Flecainide | 752.90 | Crumb et al. | 1.28 | 0 | 60.0 | 0 | 15.72 | 3.41 | 1.53 | 1 | 18.83 | - | 1 | 1 | 1 | 1 | 1 |
| Fluvoxamine | 377.00 | Mirams et al. | 48.69 | - | 60.0 | - | - | 10.56 | 12.33 | 0 | - | - | 3 | 0 | 0 | 1 | 0 |
| Gatifloxacin | 9000.00 | Ando et al. | 0.00 | - | 60.0 | - | - | 0.00 | 21.67 | 1 | - | - | 1 | 1 | 1 | 1 | 1 |
| Halofantrine | 172.00 | Kramer et al. | 21.64 | - | 60.0 | - | - | 0.16 | 3.01 | 1 | - | - | 1 | 1 | 1 | 1 | 1 |
| Haloperidol | 4.00 | Kramer et al. | 1.30 | - | 60.0 | - | - | 0.11 | 14.10 | 1 | - | - | 1 | 1 | 1 | 1 | 1 |
| Haloperidol | 3.60 | Mirams et al. | 2.33 | - | 60.0 | - | - | 0.58 | 11.25 | 1 | - | - | 1 | 1 | 1 | 1 | 1 |
| Haloperidol | 3.60 | Ando et al. | 4.41 | - | 60.0 | - | - | 1.38 | 16.67 | 1 | - | - | 1 | 1 | 1 | 1 | 1 |
| Ibutilide | 150.00 | Ando et al. | 0.04 | - | 60.0 | - | - | 0.06 | 0.18 | 1 | - | - | 1 | 1 | 1 | 1 | 1 |
| Ibutilide | 140.00 | Kramer et al. | 0.01 | - | 60.0 | - | - | 0.04 | 0.17 | 1 | - | - | 1 | 1 | 1 | 1 | 1 |
| Imipramine | 106.00 | Mirams et al. | 38.06 | - | 60.0 | - | - | 58.62 | 48.11 | 0 | - | - | 2 | 1 | 0 | 0 | 0 |
| Lamivudine | 19540.00 | Kramer et al. | 97.33 | - | 60.0 | - | - | 66.22 | 157.68 | 0 | - | - | 0 | 0 | 0 | 0 | 0 |
| Levocetirizine | 63.00 | Ando et al. | 0.00 | - | 60.0 | - | - | 0.00 | 600.00 | 0 | - | - | 0 | 0 | 0 | 0 | 1 |
| Lidocaine | 2560.00 | Crumb et al. | 60.00 | 60 | 60.0 | 60 | 100 | 60.00 | 600.00 | 0 | 60 | - | 0 | 0 | 0 | 0 | 0 |
| Linezolid | 59110.00 | Kramer et al. | 93.25 | - | 60.0 | - | - | 39.42 | 29.11 | 0 | - | - | 0 | 0 | 0 | 0 | 0 |
| Lopinavir | 703.70 | Crumb et al. | 31.72 | 0 | 60.0 | 0 | 0 | 0.00 | 10.30 | 1 | 0 | - | 0 | 0 | 0 | 0 | 0 |
| Loratadine | 0.60 | Ando et al. | 44.53 | - | 60.0 | - | - | 24.05 | 600.00 | 0 | - | - | 0 | 0 | 0 | 0 | 0 |
| Loratadine | 0.40 | Kramer et al. | 38.36 | - | 60.0 | - | - | 11.01 | 600.00 | 0 | - | - | 0 | 0 | 0 | 0 | 0 |
| Methadone | 507.00 | Kramer et al. | 3.63 | - | 60.0 | - | - | 7.82 | 10.36 | 1 | - | - | 1 | 1 | 1 | 1 | 1 |
| Metoprolol | 450.00 | Ando et al. | 0.00 | - | 60.0 | - | - | 84.47 | 483.33 | 0 | - | - | 0 | 0 | 0 | 0 | 1 |
| Metronidazole | 187000.00 | Kramer et al. | 83.21 | - | 60.0 | - | - | 49.23 | 10.75 | 0 | - | - | 0 | 0 | 0 | 0 | 0 |
| Mexiletine | 2503.20 | Crumb et al. | 75.00 | 0 | 60.0 | 0 | 97.42 | 0.00 | 47.94 | 0 | 0 | - | 0 | 0 | 0 | 0 | 0 |
| Mexiletine | 5400.00 | Ando et al. | 42.74 | - | 60.0 | - | - | 71.06 | 17.28 | 0 | - | - | 0 | 0 | 0 | 0 | 0 |
| Mexiletine | 4129.00 | Mirams et al. | 42.86 | - | 60.0 | - | - | 63.56 | 18.16 | 0 | - | - | 0 | 0 | 0 | 0 | 0 |
| Mexiletine | 4129.00 | Li et al. | 54.23 | 0 | 60.0 | 0 | 90.63 | 0.00 | 10.98 | 0 | 0 | -86 | 0 | 0 | 0 | 0 | 0 |
| Mibefradil | 63.00 | Ando et al. | 83.33 | - | 60.0 | - | - | 31.29 | 40.48 | 0 | - | - | 0 | 0 | 0 | 0 | 0 |
| Mibefradil | 12.00 | Mirams et al. | 94.54 | - | 60.0 | - | - | 73.37 | 225.00 | 0 | - | - | 0 | 0 | 0 | 0 | 0 |
| Mibefradil | 10.60 | Crumb et al. | 41.75 | 0 | 60.0 | 0 | 8.14 | 7.59 | 45.45 | 0 | 0 | - | 0 | 0 | 0 | 0 | 0 |
| Mibefradil | 12.00 | Kramer et al. | 89.63 | - | 60.0 | - | - | 20.19 | 190.05 | 0 | - | - | 0 | 0 | 0 | 0 | 0 |
| Mitoxantrone | 225.00 | Kramer et al. | 90.83 | - | 60.0 | - | - | 90.60 | 600.00 | 0 | - | - | 0 | 0 | 0 | 0 | 0 |
| Moxifloxacin | 10950.00 | Okada et al. | 22.59 | 0 | 60.0 | 6.67 | 0 | 0.39 | 19.37 | 1 | 15.07 | - | 1 | 1 | 1 | 1 | 1 |
| Moxifloxacin | 3562.50 | Crumb et al. | 0.00 | 0 | 60.0 | 78.42 | 30.76 | 0.00 | 51.33 | 1 | 0 | - | 1 | 1 | 1 | 1 | 1 |
| Moxifloxacin | 10960.00 | Kramer et al. | 43.41 | - | 60.0 | - | - | 10.66 | 12.11 | 1 | - | - | 1 | 1 | 1 | 1 | 0 |
| Moxifloxacin | 5600.00 | Ando et al. | 42.77 | - | 60.0 | - | - | 10.42 | 23.09 | 1 | - | - | 1 | 1 | 1 | 1 | 0 |
| Nifedipine | 8.00 | Kramer et al. | 99.99 | - | 60.0 | - | - | 46.60 | 600.00 | 0 | - | - | 0 | 0 | 0 | 0 | 0 |

| | | | | | | | | | | | | | | | | | |
|---|---|---|---|---|---|---|---|---|---|---|---|---|---|---|---|---|---|
| Nifedipine | 7.70 | Mirams et al. | 99.99 | - | 60.0 | - | - | 91.77 | 600.00 | 0 | - | - | 0 | 0 | 0 | 0 | 0 |
| Nifedipine | 10.00 | Ando et al. | 99.98 | - | 60.0 | - | - | 42.72 | 600.00 | 0 | - | - | 0 | 0 | 0 | 0 | 0 |
| Nilotinib | 60.40 | Crumb et al. | 0.00 | 0 | 60.0 | 0 | 3.11 | 0.00 | 2.50 | 1 | 0 | - | 2 | 1 | 0 | 0 | 1 |
| Nilotinib | 172.00 | Kramer et al. | 8.02 | - | 60.0 | - | - | 1.03 | 8.87 | 1 | - | - | 2 | 1 | 0 | 0 | 1 |
| Nilotinib | 170.00 | Ando et al. | 7.89 | - | 60.0 | - | - | 10.14 | 8.82 | 1 | - | - | 2 | 1 | 0 | 0 | 1 |
| Nitrendipine | 3.02 | Mirams et al. | 100.00 | - | 60.0 | - | - | 29.41 | 600.00 | 0 | - | - | 0 | 0 | 0 | 0 | 0 |
| Nitrendipine | 3.00 | Ando et al. | 99.93 | - | 60.0 | - | - | 63.08 | 600.00 | 0 | - | - | 0 | 0 | 0 | 0 | 0 |
| Nitrendipine | 3.00 | Kramer et al. | 99.69 | - | 60.0 | - | - | 68.58 | 600.00 | 0 | - | - | 0 | 0 | 0 | 0 | 0 |
| Ondansetron | 358.50 | Crumb et al. | 13.61 | 0 | 60.0 | 2.03 | 10.45 | 3.74 | 6.24 | 1 | 0.22 | - | 1 | 1 | 1 | 1 | 1 |
| Ondansetron | 77.00 | Ando et al. | 9.05 | - | 60.0 | - | - | 10.11 | 29.22 | 1 | - | - | 1 | 1 | 1 | 1 | 1 |
| Ondansetron | 139.00 | Li et al. | 12.90 | 0 | 60.0 | 1.92 | 9.75 | 3.47 | 14.90 | 1 | 0.2 | -82 | 1 | 1 | 1 | 1 | 1 |
| Paliperidone | 69.00 | Kramer et al. | 0.60 | - | 60.0 | - | - | 0.24 | 16.89 | 1 | - | - | 2 | 1 | 0 | 0 | 1 |
| Paliperidone | 30.00 | Ando et al. | 0.60 | - | 60.0 | - | - | 1.06 | 39.00 | 1 | - | - | 2 | 1 | 0 | 0 | 1 |
| Paroxetine | 14.00 | Kramer et al. | 36.53 | - | 60.0 | - | - | 14.59 | 187.23 | 0 | - | - | 3 | 0 | 0 | 1 | 0 |
| Pentobarbital | 5171.00 | Kramer et al. | 93.84 | - | 60.0 | - | - | 44.47 | 415.94 | 0 | - | - | 0 | 0 | 0 | 0 | 0 |
| Phenytoin | 4360.00 | Kramer et al. | 90.77 | - | 60.0 | - | - | 76.50 | 50.57 | 0 | - | - | 0 | 0 | 0 | 0 | 0 |
| Phenytoin | 4500.00 | Mirams et al. | 59.29 | - | 60.0 | - | - | 75.38 | 33.33 | 0 | - | - | 0 | 0 | 0 | 0 | 0 |
| Pimozide | 0.50 | Kramer et al. | 10.44 | - | 60.0 | - | - | 4.26 | 113.47 | 1 | - | - | 1 | 1 | 1 | 1 | 1 |
| Pimozide | 0.43 | Ando et al. | 20.00 | - | 60.0 | - | - | 5.17 | 139.53 | 1 | - | - | 1 | 1 | 1 | 1 | 1 |
| Pimozide | 1.00 | Mirams et al. | 15.62 | - | 60.0 | - | - | 35.71 | 30.00 | 1 | - | - | 1 | 1 | 1 | 1 | 1 |
| Piperacillin | 1378000.00 | Kramer et al. | 80.64 | - | 60.0 | - | - | 67.73 | 3.71 | 0 | - | - | 3 | 0 | 0 | 1 | 0 |
| Prenylamine | 3.50 | Ando et al. | 7.51 | - | 60.0 | - | - | 3.75 | 27.86 | 1 | - | - | 0 | 0 | 0 | 0 | 1 |
| Prenylamine | 17.00 | Mirams et al. | 7.29 | - | 60.0 | - | - | 3.72 | 5.74 | 1 | - | - | 0 | 0 | 0 | 0 | 1 |
| Procainamide | 52000.00 | Ando et al. | 51.20 | - | 60.0 | - | - | 35.37 | 7.86 | 1 | - | - | 1 | 1 | 1 | 1 | 0 |
| Procainamide | 54180.00 | Kramer et al. | 50.99 | - | 60.0 | - | - | 35.37 | 7.54 | 1 | - | - | 1 | 1 | 1 | 1 | 0 |
| Propafenone | 241.00 | Mirams et al. | 26.83 | - | 60.0 | - | - | 35.68 | 2.74 | 1 | - | - | 0 | 0 | 0 | 0 | 1 |
| Propafenone | 131.00 | Crumb et al. | 35.50 | 0 | 60.0 | 0 | 18.87 | 17.05 | 6.10 | 1 | 0 | - | 0 | 0 | 0 | 0 | 0 |
| Propranolol | 26.00 | Mirams et al. | 19.07 | - | 60.0 | - | - | 66.89 | 163.15 | 0 | - | - | 0 | 0 | 0 | 0 | 1 |
| Quetiapine | 33.00 | Mirams et al. | 45.55 | - | 60.0 | - | - | 33.98 | 263.64 | 0 | - | - | 3 | 0 | 0 | 1 | 0 |
| Quinidine | 3200.00 | Ando et al. | 14.44 | - | 60.0 | - | - | 6.89 | 0.34 | 1 | - | - | 1 | 1 | 1 | 1 | 1 |
| Quinidine | 3237.00 | Li et al. | 11.24 | 1.74 | 60.0 | 17.85 | 9.39 | 4.65 | 0.51 | 1 | 27.38 | -64.7 | 1 | 1 | 1 | 1 | 1 |
| Quinidine | 842.90 | Crumb et al. | 0.99 | 1.1 | 60.0 | 4.09 | 5.18 | 0.85 | 0.61 | 1 | 7.67 | - | 1 | 1 | 1 | 1 | 1 |
| Quinidine | 3237.00 | Kramer et al. | 22.70 | - | 60.0 | - | - | 3.90 | 0.33 | 1 | - | - | 1 | 1 | 1 | 1 | 1 |
| Quinidine | 3235.00 | Okada et al. | 15.13 | 0 | 60.0 | 0.3 | 0 | 1.42 | 0.29 | 1 | 30.33 | - | 1 | 1 | 1 | 1 | 1 |
| Quinidine | 3237.00 | Mirams et al. | 2.80 | - | 60.0 | - | - | 2.64 | 0.14 | 1 | - | - | 1 | 1 | 1 | 1 | 1 |
| Quinine | 4300.00 | Ando et al. | 81.89 | - | 60.0 | - | - | 78.05 | 14.72 | 1 | - | - | 3 | 0 | 0 | 1 | 0 |
| Quinine | 3956.70 | Crumb et al. | 22.20 | 0 | 60.0 | 15.03 | 46.46 | 22.28 | 1.96 | 1 | 8.91 | - | 3 | 0 | 0 | 1 | 1 |
| Raltegravir | 7000.00 | Kramer et al. | 82.64 | - | 60.0 | - | - | 58.23 | 167.74 | 0 | - | - | 0 | 0 | 0 | 0 | 0 |
| Ranolazine | 2310.00 | Okada et al. | 7.35 | 0 | 60.0 | 0 | 12.01 | 16.73 | 3.01 | 0 | 0.45 | - | 3 | 0 | 0 | 1 | 1 |
| Ranolazine | 1948.20 | Li et al. | 0.00 | 0 | 60.0 | 1.86 | 61.03 | 8.83 | 6.66 | 0 | 0 | -94 | 3 | 0 | 0 | 1 | 1 |
| Ranolazine | 2300.00 | Ando et al. | 5.51 | - | 60.0 | - | - | 5.54 | 7.50 | 0 | - | - | 3 | 0 | 0 | 1 | 1 |
| Ranolazine | 1948.20 | Crumb et al. | 0.00 | 0 | 60.0 | 1.7 | 56.98 | 0.00 | 5.53 | 0 | 0 | - | 3 | 0 | 0 | 1 | 1 |
| Ribavirin | 27880.00 | Kramer et al. | 69.97 | - | 60.0 | - | - | 32.61 | 52.03 | 0 | - | - | 0 | 0 | 0 | 0 | 0 |
| Risperidone | 2.00 | Kramer et al. | 2.84 | - | 60.0 | - | - | 0.98 | 195.80 | 1 | - | - | 2 | 1 | 0 | 0 | 1 |
| Risperidone | 1.81 | Mirams et al. | 0.31 | - | 60.0 | - | - | 0.22 | 124.31 | 1 | - | - | 2 | 1 | 0 | 0 | 1 |
| Risperidone | 15.00 | Ando et al. | 1.13 | - | 60.0 | - | - | 0.89 | 26.00 | 1 | - | - | 2 | 1 | 0 | 0 | 1 |
| Ritonavir | 436.90 | Crumb et al. | 48.00 | 0 | 60.0 | 0 | 51.32 | 31.27 | 17.71 | 1 | 0 | - | 3 | 0 | 0 | 1 | 0 |
| Rufinamide | 83126.90 | Crumb et al. | 100.00 | 60 | 60.0 | 60 | 60 | 60.00 | 600.00 | 0 | 60 | - | 0 | 0 | 0 | 0 | 0 |
| Saquinavir | 130.00 | Kramer et al. | 94.18 | - | 60.0 | - | - | 79.14 | 164.56 | 1 | - | - | 2 | 1 | 0 | 0 | 0 |
| Saquinavir | 417.20 | Crumb et al. | 66.84 | 0 | 60.0 | 0 | 40.9 | 21.21 | 12.50 | 1 | 0 | - | 2 | 1 | 0 | 0 | 0 |
| Sematilide | 4400.00 | Ando et al. | 0.00 | - | 60.0 | - | - | 0.00 | 8.52 | 1 | - | - | 0 | 0 | 0 | 0 | 1 |
| Sertindole | 1.59 | Mirams et al. | 0.24 | - | 60.0 | - | - | 0.90 | 13.21 | 1 | - | - | 2 | 1 | 0 | 0 | 1 |
| Sertindole | 1.60 | Ando et al. | 0.78 | - | 60.0 | - | - | 0.71 | 30.94 | 1 | - | - | 2 | 1 | 0 | 0 | 1 |
| Sertindole | 2.00 | Kramer et al. | 0.17 | - | 60.0 | - | - | 0.25 | 22.82 | 1 | - | - | 2 | 1 | 0 | 0 | 1 |

| | | | | | | | | | | | | | | | | | |
|---|---|---|---|---|---|---|---|---|---|---|---|---|---|---|---|---|---|
| Sertindole | 1.60 | Crumb et al. | 0.00 | 0 | 60.0 | 0 | 0 | 0.00 | 11.41 | 1 | 0 | - | 2 | 1 | 0 | 0 | 1 |
| Sitagliptin | 442.00 | Kramer et al. | 64.05 | - | 60.0 | - | - | 17.67 | 592.87 | 0 | - | - | 0 | 0 | 0 | 0 | 0 |
| Solifenacin | 3.00 | Kramer et al. | 3.38 | - | 60.0 | - | - | 16.51 | 146.45 | 1 | - | - | 3 | 0 | 0 | 1 | 1 |
| Sotalol | 14680.00 | Okada et al. | 0.00 | 0 | 60.0 | 0 | 0 | 0.00 | 36.09 | 1 | 0 | - | 1 | 1 | 1 | 1 | 1 |
| Sotalol | 13000.00 | Ando et al. | 46.37 | - | 60.0 | - | - | 2.33 | 12.85 | 1 | - | - | 1 | 1 | 1 | 1 | 0 |
| Sotalol | 14686.40 | Crumb et al. | 2.77 | 2.33 | 60.0 | 1.59 | 0 | 1.08 | 9.23 | 1 | 1.74 | - | 1 | 1 | 1 | 1 | 1 |
| Sotalol | 14690.00 | Kramer et al. | 50.11 | - | 60.0 | - | - | 2.69 | 13.22 | 1 | - | - | 1 | 1 | 1 | 1 | 0 |
| Sotalol | 14690.00 | Li et al. | 3.61 | 3.32 | 60.0 | 2.27 | 0 | 1.25 | 12.50 | 1 | 2.14 | -55 | 1 | 1 | 1 | 1 | 1 |
| Sparfloxacin | 1766.00 | Kramer et al. | 27.79 | - | 60.0 | - | - | 1.32 | 19.35 | 1 | - | - | 1 | 1 | 1 | 1 | 1 |
| Sparfloxacin | 1900.00 | Ando et al. | 27.18 | - | 60.0 | - | - | 1.28 | 17.45 | 1 | - | - | 1 | 1 | 1 | 1 | 1 |
| Sunitinib | 13.00 | Kramer et al. | 3.98 | - | 60.0 | - | - | 6.28 | 138.46 | 1 | - | - | 2 | 1 | 0 | 0 | 1 |
| Tamoxifen | 18.00 | Ando et al. | 0.00 | - | 60.0 | - | - | 0.00 | 64.75 | 0 | - | - | 2 | 1 | 0 | 0 | 1 |
| Tedisamil | 85.00 | Mirams et al. | 0.00 | - | 60.0 | - | - | 15.79 | 44.12 | 1 | - | - | 0 | 0 | 0 | 0 | 1 |
| Telbivudine | 19720.00 | Kramer et al. | 47.04 | - | 60.0 | - | - | 36.67 | 32.15 | 0 | - | - | 0 | 0 | 0 | 0 | 0 |
| Terfenadine | 9.00 | Kramer et al. | 0.92 | - | 60.0 | - | - | 0.23 | 7.68 | 1 | - | - | 1 | 1 | 1 | 1 | 1 |
| Terfenadine | 9.00 | Mirams et al. | 3.44 | - | 60.0 | - | - | 1.36 | 1.48 | 1 | - | - | 1 | 1 | 1 | 1 | 1 |
| Terfenadine | 9.00 | Okada et al. | 2.16 | 0 | 60.0 | 0 | 0 | 34.55 | 15.45 | 1 | 0 | - | 1 | 1 | 1 | 1 | 1 |
| Terfenadine | 4.00 | Li et al. | 12.81 | 0 | 60.0 | 1.05 | 2.52 | 0.93 | 11.30 | 1 | 7.09 | -77 | 1 | 1 | 1 | 1 | 1 |
| Terfenadine | 9.50 | Ando et al. | 7.46 | - | 60.0 | - | - | 3.61 | 7.89 | 1 | - | - | 1 | 1 | 1 | 1 | 1 |
| Terfenadine | 9.00 | Crumb et al. | 11.39 | 0 | 60.0 | 0.96 | 2.25 | 0.77 | 4.15 | 1 | 6.72 | - | 1 | 1 | 1 | 1 | 1 |
| Terodiline | 240.00 | Ando et al. | 16.88 | - | 60.0 | - | - | 11.64 | 4.06 | 1 | - | - | 0 | 0 | 0 | 0 | 1 |
| Terodiline | 145.00 | Kramer et al. | 16.55 | - | 60.0 | - | - | 7.57 | 6.67 | 1 | - | - | 0 | 0 | 0 | 0 | 1 |
| Thioridazine | 45.00 | Ando et al. | 17.65 | - | 60.0 | - | - | 34.88 | 16.67 | 1 | - | - | 1 | 1 | 1 | 1 | 1 |
| Thioridazine | 980.00 | Kramer et al. | 11.22 | - | 60.0 | - | - | 33.55 | 0.77 | 1 | - | - | 1 | 1 | 1 | 1 | 1 |
| Thioridazine | 979.00 | Mirams et al. | 3.67 | - | 60.0 | - | - | 2.63 | 0.05 | 1 | - | - | 1 | 1 | 1 | 1 | 1 |
| Tolterodine | 0.14 | Ando et al. | 15.13 | - | 60.0 | - | - | 2.49 | 182.14 | 0 | - | - | 2 | 1 | 0 | 0 | 1 |
| Toremifene | 26.30 | Crumb et al. | 0.00 | 0 | 60.0 | 0 | 56.76 | 0.00 | 199.62 | 1 | 0 | - | 2 | 1 | 0 | 0 | 1 |
| Vandetanib | 110.00 | Ando et al. | 0.00 | - | 60.0 | - | - | 0.00 | 5.45 | 1 | - | - | 1 | 1 | 1 | 1 | 1 |
| Verapamil | 45.00 | Crumb et al. | 80.22 | 1.93 | 60.0 | 0 | 9.31 | 0.00 | 16.03 | 0 | 8.79 | - | 0 | 0 | 0 | 0 | 0 |
| Verapamil | 81.00 | Mirams et al. | 68.20 | - | 60.0 | - | - | 0.51 | 2.65 | 0 | - | - | 0 | 0 | 0 | 0 | 0 |
| Verapamil | 88.00 | Kramer et al. | 63.25 | - | 60.0 | - | - | 0.28 | 4.48 | 0 | - | - | 0 | 0 | 0 | 0 | 0 |
| Verapamil | 87.97 | Okada et al. | 46.74 | 0 | 60.0 | 1.34 | 0 | 9.40 | 3.38 | 0 | 7.92 | - | 0 | 0 | 0 | 0 | 0 |
| Verapamil | 92.00 | Ando et al. | 65.22 | - | 60.0 | - | - | 1.14 | 4.08 | 0 | - | - | 0 | 0 | 0 | 0 | 0 |
| Verapamil | 81.00 | Li et al. | 69.88 | 1.66 | 60.0 | 0 | 5.79 | 0.00 | 5.33 | 0 | 6.01 | -100 | 0 | 0 | 0 | 0 | 0 |
| Voriconazole | 7563.00 | Kramer et al. | 64.00 | - | 60.0 | - | - | 32.20 | 97.36 | 0 | - | - | 3 | 0 | 0 | 1 | 0 |
| Ziprasidone | 7.30 | Ando et al. | 0.00 | - | 60.0 | - | - | 0.21 | 49.32 | 1 | - | - | 3 | 0 | 0 | 1 | 1 |

**Table S9.** Merged Dataset. The table reports the block of seven ion channel currents ($I_{CaV}$, $I_{Kr}$, $I_{K1}$, $I_{Ks}$, $I_{to}$, $I_{Na}$ and $I_{NaL}$) at $C_{Drug}$ = $IC_{60,hERG}$, the maximum EFTPC value of each of the drugs (“EFTPC” column) , the refrence/source (“Source” column) used to extract the $IC_{50}$, Hill coefficient and EFTPC values for the particular drug, the hERG ration (“Ratio” column), the drug-trapping parameter values for the available drugs (“vtrap”column) and the different risk definition assigned to them (’target’, ’CM’, ’t1’, ’t2’) columns. target- TdP+:- R1, R2 , R3, CH1, CM1 category or label warning , t1- TdP+: Drugs in CM1 and CM2 category, t2- TdP+: Drugs in CM1 category, t3- TdP+: Drugs in CM1 and CM3 category.

66

**Dataset 9: Drugs in Fermini et al. 2016** A working group under the CiPA initiative led by FDA have recently categorized 28 drugs into tertiary risk categories (Fermini et al., 2016). Twelve of the twenty eight drugs are common to the Dataset 5 and 7. We extract *in-vitro* assay data for these drugs from Datasets 1, 2, 3, 5, 6 and 7. We used the mean value of the block for a particular channel if the information for the drug was present in more than one dataset. No information was available for two (Azimilide and Loratidine) of the 28 drugs in Datasets 1, 2, 3, 5, 6 and 7 and hence a final dataset of 26 drugs was obtained. If the value is not available it was available for particular ion channels in any of the datasets described here, it was set to zero. Drug-trapping parameter for 12 of these drugs reported in Dataset 7 was also also added to one of the columns of the dataset.

| | $EFTPC$ | $IC_{50,I_{CaV}}$ | $h_{I_{CaV}}$ | $h_{I_{K1}}$ | $h_{I_{Kr}}$ | $h_{I_{Ks}}$ | $h_{I_{NaL}}$ | $h_{I_{Na,peak}}$ | $h_{I_{to}}$ | $IC_{50,I_{K1}}$ | $IC_{50,I_{Kr}}$ | $IC_{50,I_{Ks}}$ | $IC_{50,I_{Na,late}}$ | $IC_{50,I_{Na,peak}}$ | $Target$ | $IC_{50,I_{to}}$ | $vtrap$ |
|---|---|---|---|---|---|---|---|---|---|---|---|---|---|---|---|---|---|
| Astemizole | 0.37 | 1062.6 | 1.73 | 1.00 | 1.18 | 1.00 | 1.00 | 1.49 | 1.00 | - | 12.04 | - | - | 2620.67 | 1 | - | - |
| Bepridil | 33.69 | 1547 | 1.13 | 1.00 | 1.02 | 1.03 | 1.13 | 1.14 | 1.98 | - | 113.70 | 21095.9 | 1814 | 2467.47 | 2 | 7236.33 | -54.93 |
| Chlorpromazine | 37.30 | 5796 | 1.07 | 0.88 | 1.02 | 1.00 | 0.96 | 1.43 | 0.76 | 9269.95 | 1303.44 | - | 4560 | 3874.2 | 1 | 1.76167e+07 | -14 |
| Cisapride | 3.05 | 3.70881e+06 | 0.86 | 0.83 | 1.06 | 0.77 | 1.00 | 0.98 | 0.73 | 29498 | 13.87 | 8.11929e+07 | - | 172693 | 1 | 219112 | -199 |
| Clarithromycin | 3300.00 | - | 1.00 | 1.00 | 1.00 | 1.00 | 1.00 | 1.00 | 1.00 | - | 32900.00 | - | - | - | 1 | - | - |
| Clozapine | 195.50 | 3600 | 1.00 | 1.00 | 0.98 | 1.00 | 1.00 | 1.07 | 1.00 | - | 2300.00 | - | - | 15100 | 1 | - | - |
| Diltiazem | 134.70 | 438.8 | 0.91 | 1.00 | 0.97 | 1.00 | 0.88 | 0.94 | 0.68 | - | 12683.80 | - | 21865.5 | 55103.6 | 0 | 2.8e+09 | -90 |
| Dofetilide | 1.95 | 49670.1 | 1.02 | 0.93 | 1.11 | 1.00 | 0.88 | 0.85 | 0.93 | 394.3 | 18.20 | - | 753160 | 149816 | 2 | 18.8 | -1 |
| Domperidone | 27.00 | - | 1.00 | 1.00 | 1.00 | 1.00 | 1.00 | 1.00 | 1.00 | - | 160.00 | - | - | 5600 | 1 | - | - |
| Droperidol | 18.50 | 7600 | 1.08 | 1.00 | 1.05 | 1.00 | 1.00 | 1.12 | 1.00 | - | 60.00 | - | - | 22700 | 1 | - | - |
| Ibutilide | 145.00 | 62500 | 1.08 | 1.00 | 1.27 | 1.00 | 1.00 | 1.02 | 1.00 | - | 18.00 | - | - | 42500 | 2 | - | - |
| Methadone | 507.00 | 37400 | 1.67 | 1.00 | 1.00 | 1.00 | 1.00 | 1.37 | 1.00 | - | 3500.00 | - | - | 31800 | 2 | - | - |
| Metoprolol | 450.00 | - | 1.00 | 1.00 | 1.00 | 1.00 | 1.00 | 1.00 | 1.00 | - | 145000.00 | - | - | 40000 | 0 | - | - |
| Mexiletine | 4040.30 | 75810.9 | 1.00 | 1.00 | 0.98 | 1.00 | 1.20 | 1.00 | 1.00 | - | 55270.00 | - | 8956.9 | 40500 | 0 | - | -86 |
| Nifedipine | 8.57 | 28 | 1.01 | 1.00 | 0.93 | 1.00 | 1.00 | 0.90 | 1.00 | - | 121000.00 | - | - | 71333.3 | 0 | - | - |
| Nitrendipine | 3.01 | 16.7833 | 0.93 | 1.00 | 0.94 | 1.00 | 1.00 | 1.08 | 1.00 | - | 19733.33 | - | - | 26400 | 0 | - | - |
| Ondansetron | 191.50 | 22567.3 | 0.87 | 1.00 | 0.97 | 0.80 | 1.00 | 1.00 | 1.00 | - | 1437.33 | 569807 | 19181 | 45110.9 | 1 | 1.02338e+06 | -82 |
| Pimozide | 0.64 | 214 | 1.16 | 1.00 | 1.05 | 1.00 | 1.00 | 1.02 | 1.00 | - | 33.33 | - | - | 751.333 | 1 | - | - |
| Quinidine | 2831.48 | 23219.3 | 0.85 | 0.80 | 0.98 | 1.19 | 1.05 | 1.25 | 1.13 | 3.95899e+07 | 618.78 | 27709.3 | 9417 | 15874.7 | 2 | 2960.13 | -64.7 |
| Ranolazine | 2126.60 | 207150 | 0.97 | 1.00 | 0.85 | 0.75 | 1.08 | 1.08 | 1.08 | - | 7547.50 | 3.6155e+07 | 14009.3 | 134630 | 0 | 433510 | -94 |
| Risperidone | 6.27 | 47133.3 | 0.93 | 1.00 | 1.00 | 1.00 | 1.00 | 0.99 | 1.00 | - | 223.33 | - | - | 62933.3 | 1 | - | - |
| Sotalol | 14349.28 | 3.62741e+06 | 0.96 | 1.08 | 0.89 | 1.08 | 1.00 | 0.80 | 0.88 | 3.05026e+06 | 155233.80 | 4.22186e+06 | - | 5.73507e+08 | 2 | 4.31435e+07 | -55 |
| Tamoxifen | 18.00 | - | 1.00 | 1.00 | 1.00 | 1.00 | 1.00 | 1.00 | 1.00 | - | 777.00 | - | - | - | 0 | - | - |
| Terfenadine | 8.25 | 749.417 | 1.22 | 1.00 | 0.94 | 0.83 | 0.87 | 1.05 | 0.77 | - | 41.57 | 399754 | 20056 | 2509.5 | 1 | 239961 | -77 |
| Vandetanib | 110.00 | - | 1.00 | 1.00 | 1.00 | 1.00 | 1.00 | 1.00 | 1.00 | - | 400.00 | - | - | - | 2 | - | - |
| Verapamil | 79.16 | 206.017 | 1.02 | 0.77 | 1.01 | 0.99 | 1.00 | 1.03 | 0.93 | 3.49e+08 | 271.88 | 29880 | 7028 | 27693 | 0 | 10127.1 | -100 |

**Table S10.** $IC_{50}$, Hill coefficients, EFTPC values, drug trapping parameters and risk categorization for the (Fermini et al., 2016) dataset. All values extracted from Dataset 1, 2, 3, 5, 6, 7. If the value was presented in more than one dataset an average of those values was taken. Target column represents the tertiary risk categorization assigned in Fermini et al., 2-High risk, 1-Intermediate risk and 0-Low risk

## 1.2 Binary Risk Definition assigned

| | CredibleMeds | Redfern et al. 2013 | Champeroux et al. 2005 | label | target |
|---|---|---|---|---|---|
| Ajmaline | 0 | 0 | 1 | - | 1 |
| Amiodarone | 1 | 1 | 0 | - | 1 |
| Amitriptyline | 3 | 4 | 0 | QT | 0 |
| Aspirin | 0 | 0 | 3 | na | 0 |
| Astemizole | 1 | 2 | 1 | - | 1 |
| Azithromycin | 1 | 0 | 2 | - | 1 |
| Bepridil | 1 | 3 | 1 | - | 1 |
| Ceftriaxone | 0 | 0 | 0 | na | 0 |
| Chloroquine | 1 | 0 | 2 | - | 1 |
| Chlorpheniramine | 0 | 5 | 1 | - | 1 |
| Chlorpromazine | 1 | 0 | 0 | - | 1 |
| Cibenzoline | 0 | 5 | 0 | na | 0 |
| Cilostazol | 1 | 0 | 0 | - | 1 |
| Cisapride | 1 | 2 | 1 | - | 1 |
| Clarithromycin | 1 | 4 | 2 | - | 1 |
| Clozapine | 2 | 0 | 2 | TdP+QT | 1 |
| Desipramine | 2 | 4 | 0 | QT | 0 |
| Diazepam | 0 | 0 | 3 | na | 0 |
| Diltiazem | 0 | 5 | 3 | na | 0 |
| Diphenhydramine | 3 | 4 | 0 | na | 0 |
| Disopyramide | 1 | 1 | 1 | - | 1 |
| Dofetilide | 1 | 1 | 1 | - | 1 |
| Dolasetron | 2 | 0 | 0 | TdP+QT | 1 |
| Domperidone | 1 | 4 | 0 | - | 1 |
| Donepezil | 1 | 0 | 0 | - | 1 |
| Dosatinib | 2 | 0 | 0 | QT | 0 |
| Dronedarone | 1 | 0 | 0 | - | 1 |
| Droperidol | 1 | 0 | 1 | - | 1 |
| Duloxetine | 0 | 0 | 0 | na | 0 |
| E4031 | 0 | 0 | 0 | - | 1 |
| Erythromycin | 1 | 3 | 1 | - | 1 |
| Famotidine | 2 | 0 | 0 | na | 0 |
| Flecainide | 1 | 3 | 0 | - | 1 |
| Fluvoxamine | 3 | 0 | 0 | TdP | 0 |
| Gatifloxacin | 1 | 0 | 0 | - | 1 |
| Halofantrine | 1 | 0 | 1 | - | 1 |
| Haloperidol | 1 | 3 | 0 | - | 1 |
| Ibutilide | 1 | 1 | 0 | - | 1 |
| Imipramine | 2 | 4 | 1 | CYP2D6inhibition | 0 |
| Lamivudine | 0 | 0 | 0 | na | 0 |
| Levocetirizine | 0 | 0 | 0 | na | 0 |
| Lidocaine | 0 | 0 | 0 | na | 0 |
| Linezolid | 0 | 0 | 0 | na | 0 |
| Lopinavir | 0 | 0 | 0 | TdP+QT | 1 |
| Loratadine | 0 | 5 | 0 | na | 0 |
| Methadone | 1 | 0 | 0 | TdP+QT | 1 |
| Metoprolol | 0 | 0 | 0 | na | 0 |
| Metronidazole | 0 | 0 | 0 | na | 0 |
| Mexiletine | 0 | 0 | 0 | na | 0 |
| Mibefradil | 0 | 4 | 0 | na | 0 |
| Mitoxantrone | 0 | 0 | 0 | na | 0 |
| Moxifloxacin | 1 | 0 | 2 | - | 1 |
| Nifedipine | 0 | 4 | 0 | na | 0 |
| Nilotinib | 2 | 0 | 0 | TdP+QT | 1 |
| Nitrendipine | 0 | 5 | 0 | na | 0 |
| Ondansetron | 1 | 0 | 2 | - | 1 |
| Paliperidone | 2 | 0 | 0 | TdP+QT | 1 |
| Paroxetine | 3 | 0 | 0 | CYP2D6inhibition | 0 |
| Pentobarbital | 0 | 0 | 0 | na | 0 |
| Phenytoin | 0 | 5 | 3 | na | 0 |
| Pimozide | 1 | 3 | 1 | - | 1 |
| Piperacillin | 3 | 0 | 0 | na | 0 |
| Prenylamine | 0 | 0 | 1 | - | 1 |
| Procainamide | 1 | 1 | 0 | - | 1 |
| Propafenone | 0 | 4 | 1 | - | 1 |
| Propranolol | 0 | 0 | 3 | na | 0 |
| Quetiapine | 3 | 0 | 0 | na | 0 |
| Quinidine | 1 | 1 | 1 | - | 1 |
| Quinine | 3 | 0 | 0 | TdP+QT | 1 |
| Raltegravir | 0 | 0 | 0 | na | 0 |
| Ranolazine | 3 | 0 | 0 | QT | 0 |
| Ribavirin | 0 | 0 | 0 | na | 0 |
| Risperidone | 2 | 5 | 2 | TdP+QT | 1 |
| Ritonavir | 3 | 0 | 0 | TdP+QT | 1 |
| Rufinamide | 0 | 0 | 0 | QTshort | 0 |
| Saquinavir | 2 | 0 | 0 | TdP+QT | 1 |
| Sematilide | 0 | 1 | 0 | - | 1 |
| Sertindole | 2 | 2 | 1 | - | 1 |
| Sitagliptin | 0 | 0 | 0 | na | 0 |
| Solifenacin | 3 | 0 | 0 | TdP+QT | 1 |
| Sotalol | 1 | 1 | 1 | - | 1 |
| Sparfloxacin | 1 | 4 | 1 | - | 1 |
| Sunitinib | 2 | 0 | 0 | TdP+QT | 1 |
| Tamoxifen | 2 | 5 | 0 | QT | 0 |
| Tedisamil | 0 | 1 | 0 | - | 1 |
| Telbivudine | 0 | 0 | 0 | na | 0 |
| Terfenadine | 1 | 2 | 1 | - | 1 |
| Terodiline | 0 | 2 | 0 | - | 1 |
| Thioridazine | 1 | 3 | 1 | - | 1 |
| Tolterodine | 2 | 0 | 0 | QT | 0 |
| Toremifene | 2 | 0 | 0 | TdP+QT | 1 |
| Vandetanib | 1 | 0 | 0 | - | 1 |
| Verapamil | 0 | 5 | 2 | na | 0 |
| Voriconazole | 3 | 0 | 0 | na | 0 |
| Ziprasidone | 3 | 0 | 0 | TdP+QT | 1 |

**Table S11.** Binary risk definition assigned to the drugs. **CredibleMeds column**: 0: Drug not analyzed in CrediblIMeds or not assigned to any category, 1: CM1, 2: CM2, 3: CM3. **Redfern et al. column**: 0: drug absent from the list of drugs reported in Redfern et al., 1: R1, 2: R2, 3: R3, 4: R4, 5: R5. **Champeroux et al. column**: 0: drug absent in the list of drugs reported in Champeroux et al., 1: CH1, 2: CH2, 3: CH3. **Target column-** Final risk assigned to the drugs in this study. 1: TdP+, 0: TdP-

77 ## 1.3 Drugs included in more than one dataset

| | Source | % block $I_{Kr}$ | % block $I_{CaV}$ | % block $I_{Na,peak}$ | $\frac{IC_{60,hERG}}{EFTPC}$ | Risk(O) | Risk | Prediction | EAD |
|---|---|---|---|---|---|---|---|---|---|
| Ajmaline | Mirams et al. | 60.0 | 2.15 | 15.98 | 1.04 | 1 | 1 | 1 | 1 |
| Ajmaline | Ando et al. | 60.0 | 2.15 | 15.98 | 62.40 | 0 | 1 | 1 | 1 |
| Amiodarone | Kramer et al. | 60.0 | 42.79 | 7.81 | 1559.41 | 1 | 1 | 0 | 0 |
| Amiodarone | Ando et al. | 60.0 | 40.44 | 7.50 | 8.60 | 1 | 1 | 1 | 0 |
| Amiodarone | Mirams et al. | 60.0 | 14.29 | 0.93 | 90.00 | 1 | 1 | 1 | 1 |
| Amiodarone | Okada et al. | 60.0 | 23.95 | 54.49 | 1602.42 | 0 | 1 | 0 | 0 |
| Amiodarone | Crumb et al. | 60.0 | 55.49 | 34.65 | 2642.27 | - | 1 | 0 | 0 |
| Amitriptyline | Mirams et al. | 60.0 | 29.78 | 19.74 | 120.00 | 0 | 0 | 1 | 1 |
| Amitriptyline | Ando et al. | 60.0 | 42.86 | 84.47 | 212.20 | 0 | 0 | 0 | 0 |
| Amitriptyline | Crumb et al. | 60.0 | 80.96 | 48.44 | 150.82 | - | 0 | 0 | 0 |
| Astemizole | Okada et al. | 60.0 | 0.02 | 0.23 | 123.26 | 1 | 1 | 1 | 1 |
| Astemizole | Ando et al. | 60.0 | 0.54 | 0.20 | 11.76 | 1 | 1 | 1 | 1 |
| Astemizole | Kramer et al. | 60.0 | 0.02 | 0.00 | 22.42 | 1 | 1 | 1 | 1 |
| Bepridil | Li et al. | 60.0 | 10.46 | 1.28 | 2.38 | 1 | 1 | 1 | 1 |
| Bepridil | Mirams et al. | 60.0 | 19.00 | 1.32 | 1.50 | 1 | 1 | 1 | 1 |
| Bepridil | Okada et al. | 60.0 | 0.70 | 17.79 | 4.96 | 1 | 1 | 1 | 1 |
| Bepridil | Crumb et al. | 60.0 | 18.37 | 4.59 | 7.42 | - | 1 | 1 | 1 |
| Bepridil | Ando et al. | 60.0 | 19.35 | 9.45 | 6.86 | 1 | 1 | 1 | 1 |
| Bepridil | Kramer et al. | 60.0 | 14.73 | 5.85 | 7.25 | 1 | 1 | 1 | 1 |
| Chlorpromazine | Mirams et al. | 60.0 | 0.00 | 33.90 | 58.03 | 1 | 1 | 1 | 1 |
| Chlorpromazine | Crumb et al. | 60.0 | 22.57 | 13.01 | 50.85 | - | 1 | 1 | 1 |
| Chlorpromazine | Ando et al. | 60.0 | 39.82 | 42.86 | 59.21 | 1 | 1 | 1 | 0 |
| Chlorpromazine | Li et al. | 60.0 | 20.82 | 10.37 | 40.59 | 0 | 1 | 1 | 1 |
| Chlorpromazine | Kramer et al. | 60.0 | 28.61 | 38.70 | 52.73 | 1 | 1 | 1 | 1 |
| Cibenzoline | Mirams et al. | 60.0 | 53.05 | 81.29 | 34.73 | 0 | 0 | 0 | 0 |
| Cibenzoline | Crumb et al. | 60.0 | 0.00 | 9.39 | 4.89 | - | 0 | 1 | 1 |
| Cilostazol | Ando et al. | 60.0 | 18.50 | 18.09 | 147.86 | 1 | 1 | 1 | 1 |
| Cilostazol | Kramer et al. | 60.0 | 19.11 | 18.70 | 168.34 | 1 | 1 | 0 | 0 |
| Cisapride | Okada et al. | 60.0 | 0.06 | 1.55 | 7.75 | 1 | 1 | 1 | 1 |
| Cisapride | Mirams et al. | 60.0 | 0.00 | 0.07 | 1.99 | 1 | 1 | 1 | 1 |
| Cisapride | Crumb et al. | 60.0 | 0.50 | 0.00 | 6.30 | - | 1 | 1 | 1 |
| Cisapride | Ando et al. | 60.0 | 0.25 | 0.01 | 11.54 | 1 | 1 | 1 | 1 |
| Cisapride | Li et al. | 60.0 | 0.51 | 0.00 | 6.86 | 0 | 1 | 1 | 1 |
| Cisapride | Kramer et al. | 60.0 | 0.25 | 0.01 | 9.85 | 1 | 1 | 1 | 1 |
| Clozapine | Ando et al. | 60.0 | 48.94 | 18.60 | 10.78 | 0 | 1 | 0 | 0 |
| Clozapine | Kramer et al. | 60.0 | 49.25 | 15.86 | 49.20 | 1 | 1 | 0 | 0 |
| Diltiazem | Mirams et al. | 60.0 | 98.30 | 74.25 | 212.70 | 0 | 0 | 0 | 0 |
| Diltiazem | Crumb et al. | 60.0 | 96.10 | 16.47 | 85.53 | - | 0 | 0 | 0 |
| Diltiazem | Ando et al. | 60.0 | 96.30 | 46.92 | 110.00 | 0 | 0 | 0 | 0 |
| Diltiazem | Li et al. | 60.0 | 97.47 | 23.56 | 169.13 | 0 | 0 | 0 | 0 |

| | | | | | | | | | |
|---|---|---|---|---|---|---|---|---|---|
| Diltiazem | Kramer et al. | 60.0 | 97.47 | 44.24 | 153.47 | 0 | 0 | 0 | 0 |
| Diphenhydramine | Ando et al. | 60.0 | 3.31 | 15.98 | 136.84 | 0 | 0 | 1 | 1 |
| Diphenhydramine | Mirams et al. | 60.0 | 3.31 | 15.98 | 229.41 | 0 | 0 | 0 | 0 |
| Disopyramide | Ando et al. | 60.0 | 2.04 | 11.37 | 28.80 | 1 | 1 | 1 | 1 |
| Disopyramide | Kramer et al. | 60.0 | 2.12 | 10.02 | 30.30 | 1 | 1 | 1 | 1 |
| Dofetilide | Crumb et al. | 60.0 | 0.75 | 0.00 | 0.94 | - | 1 | 1 | 1 |
| Dofetilide | Okada et al. | 60.0 | 0.06 | 6.96 | 29.35 | 1 | 1 | 1 | 1 |
| Dofetilide | Mirams et al. | 60.0 | 0.01 | 0.00 | 3.75 | 1 | 1 | 1 | 1 |
| Dofetilide | Li et al. | 60.0 | 1.44 | 2.90 | 3.84 | 1 | 1 | 1 | 1 |
| Dofetilide | Ando et al. | 60.0 | 0.17 | 0.03 | 22.50 | 1 | 1 | 1 | 1 |
| Dofetilide | Kramer et al. | 60.0 | 0.16 | 0.03 | 21.03 | 1 | 1 | 1 | 1 |
| Droperidol | Ando et al. | 60.0 | 1.17 | 0.39 | 4.29 | 1 | 1 | 1 | 1 |
| Droperidol | Kramer et al. | 60.0 | 0.55 | 0.10 | 5.42 | 1 | 1 | 1 | 1 |
| E4031 | Okada et al. | 60.0 | 1.05 | 3.39 | 5.68 | 1 | 1 | 1 | 1 |
| E4031 | Ando et al. | 60.0 | 0.00 | 0.00 | 2.50 | 1 | 1 | 1 | 1 |
| Flecainide | Ando et al. | 60.0 | 7.67 | 26.63 | 1.88 | 1 | 1 | 1 | 1 |
| Flecainide | Crumb et al. | 60.0 | 1.28 | 3.41 | 1.53 | - | 1 | 1 | 1 |
| Flecainide | Kramer et al. | 60.0 | 8.62 | 25.11 | 3.16 | 1 | 1 | 1 | 1 |
| Haloperidol | Kramer et al. | 60.0 | 1.30 | 0.11 | 14.10 | 1 | 1 | 1 | 1 |
| Haloperidol | Ando et al. | 60.0 | 4.41 | 1.38 | 16.67 | 1 | 1 | 1 | 1 |
| Haloperidol | Mirams et al. | 60.0 | 2.33 | 0.58 | 11.25 | 1 | 1 | 1 | 1 |
| Ibutilide | Ando et al. | 60.0 | 0.04 | 0.06 | 0.18 | 1 | 1 | 1 | 1 |
| Ibutilide | Kramer et al. | 60.0 | 0.01 | 0.04 | 0.17 | 1 | 1 | 1 | 1 |
| Imipramine | Lanacster and Sobie | 60.0 | 38.06 | 58.62 | 48.11 | 1 | 0 | 1 | 0 |
| Imipramine | Mirams et al. | 60.0 | 38.06 | 58.62 | 48.11 | 0 | 0 | 1 | 0 |
| Loratadine | Ando et al. | 60.0 | 44.53 | 24.05 | 15250.00 | 0 | 0 | 0 | 0 |
| Loratadine | Kramer et al. | 60.0 | 38.36 | 11.01 | 20209.49 | 0 | 0 | 0 | 0 |
| Mexiletine | Ando et al. | 60.0 | 42.74 | 71.06 | 17.28 | 0 | 0 | 1 | 0 |
| Mexiletine | Mirams et al. | 60.0 | 42.86 | 63.56 | 18.16 | 0 | 0 | 1 | 0 |
| Mexiletine | Li et al. | 60.0 | 54.23 | 0.00 | 10.98 | 0 | 0 | 0 | 0 |
| Mexiletine | Crumb et al. | 60.0 | 75.00 | 0.00 | 47.94 | - | 0 | 0 | 0 |
| Mibefradil | Kramer et al. | 60.0 | 89.63 | 20.19 | 190.05 | 0 | 0 | 0 | 0 |
| Mibefradil | Ando et al. | 60.0 | 83.33 | 31.29 | 40.48 | 0 | 0 | 0 | 0 |
| Mibefradil | Mirams et al. | 60.0 | 94.54 | 73.37 | 225.00 | 0 | 0 | 0 | 0 |
| Mibefradil | Crumb et al. | 60.0 | 41.75 | 7.59 | 45.45 | - | 0 | 1 | 0 |
| Moxifloxacin | Crumb et al. | 60.0 | 0.00 | 0.00 | 51.33 | - | 1 | 1 | 1 |
| Moxifloxacin | Ando et al. | 60.0 | 42.77 | 10.42 | 23.09 | 1 | 1 | 1 | 0 |
| Moxifloxacin | Kramer et al. | 60.0 | 43.41 | 10.66 | 12.11 | 1 | 1 | 1 | 0 |
| Moxifloxacin | Okada et al. | 60.0 | 22.59 | 0.39 | 19.37 | 0 | 1 | 1 | 1 |
| Nifedipine | Kramer et al. | 60.0 | 99.99 | 46.60 | 9130.13 | 0 | 0 | 0 | 0 |

| | | | | | | | | | |
|---|---|---|---|---|---|---|---|---|---|
| Nifedipine | Ando et al. | 60.0 | 99.98 | 42.72 | 6600.00 | 0 | 0 | 0 | 0 |
| Nifedipine | Mirams et al. | 60.0 | 99.99 | 91.77 | 53571.43 | 0 | 0 | 0 | 0 |
| Nilotinib | Ando et al. | 60.0 | 7.89 | 10.14 | 8.82 | 0 | 1 | 1 | 1 |
| Nilotinib | Kramer et al. | 60.0 | 8.02 | 1.03 | 8.87 | 1 | 1 | 1 | 1 |
| Nilotinib | Crumb et al. | 60.0 | 0.00 | 0.00 | 2.50 | - | 1 | 1 | 1 |
| Nitrendipine | Ando et al. | 60.0 | 99.93 | 63.08 | 12300.00 | 0 | 0 | 0 | 0 |
| Nitrendipine | Kramer et al. | 60.0 | 99.69 | 68.58 | 13444.95 | 0 | 0 | 0 | 0 |
| Nitrendipine | Mirams et al. | 60.0 | 100.00 | 29.41 | 4966.89 | 0 | 0 | 0 | 0 |
| Ondansetron | Crumb et al. | 60.0 | 13.61 | 3.74 | 6.24 | - | 1 | 1 | 1 |
| Ondansetron | Li et al. | 60.0 | 12.90 | 3.47 | 14.90 | 0 | 1 | 1 | 1 |
| Ondansetron | Ando et al. | 60.0 | 9.05 | 10.11 | 29.22 | 1 | 1 | 1 | 1 |
| Paliperidone | Ando et al. | 60.0 | 0.60 | 1.06 | 39.00 | 0 | 1 | 1 | 1 |
| Paliperidone | Kramer et al. | 60.0 | 0.60 | 0.24 | 16.89 | 1 | 1 | 1 | 1 |
| Phenytoin | Kramer et al. | 60.0 | 90.77 | 76.50 | 50.57 | 0 | 0 | 0 | 0 |
| Phenytoin | Mirams et al. | 60.0 | 59.29 | 75.38 | 33.33 | 0 | 0 | 0 | 0 |
| Pimozide | Kramer et al. | 60.0 | 10.44 | 4.26 | 113.47 | 1 | 1 | 1 | 1 |
| Pimozide | Mirams et al. | 60.0 | 15.62 | 35.71 | 30.00 | 1 | 1 | 1 | 1 |
| Pimozide | Ando et al. | 60.0 | 20.00 | 5.17 | 139.53 | 1 | 1 | 1 | 1 |
| Prenylamine | Ando et al. | 60.0 | 7.51 | 3.75 | 27.86 | 1 | 1 | 1 | 1 |
| Prenylamine | Mirams et al. | 60.0 | 7.29 | 3.72 | 5.74 | 1 | 1 | 1 | 1 |
| Procainamide | Kramer et al. | 60.0 | 50.99 | 35.37 | 7.54 | 1 | 1 | 0 | 0 |
| Procainamide | Ando et al. | 60.0 | 51.20 | 35.37 | 7.86 | 1 | 1 | 0 | 0 |
| Propafenone | Lanacster and Sobie | 60.0 | 26.83 | 35.68 | 2.74 | 1 | 1 | 1 | 1 |
| Propafenone | Mirams et al. | 60.0 | 26.83 | 35.68 | 2.74 | 0 | 1 | 1 | 1 |
| Propafenone | Crumb et al. | 60.0 | 35.50 | 17.05 | 6.10 | - | 1 | 1 | 0 |
| Quinidine | Ando et al. | 60.0 | 14.44 | 6.89 | 0.34 | 1 | 1 | 1 | 1 |
| Quinidine | Okada et al. | 60.0 | 15.13 | 1.42 | 0.29 | 1 | 1 | 1 | 1 |
| Quinidine | Li et al. | 60.0 | 11.24 | 4.65 | 0.51 | 1 | 1 | 1 | 1 |
| Quinidine | Mirams et al. | 60.0 | 2.80 | 2.64 | 0.14 | 1 | 1 | 1 | 1 |
| Quinidine | Kramer et al. | 60.0 | 22.70 | 3.90 | 0.33 | 1 | 1 | 1 | 1 |
| Quinidine | Crumb et al. | 60.0 | 0.99 | 0.85 | 0.61 | - | 1 | 1 | 1 |
| Quinine | Ando et al. | 60.0 | 81.89 | 78.05 | 14.72 | 0 | 1 | 0 | 0 |
| Quinine | Crumb et al. | 60.0 | 22.20 | 22.28 | 1.96 | - | 1 | 1 | 1 |
| Ranolazine | Okada et al. | 60.0 | 7.35 | 16.73 | 3.01 | 0 | 0 | 1 | 1 |
| Ranolazine | Crumb et al. | 60.0 | 0.00 | 0.00 | 5.53 | - | 0 | 1 | 1 |
| Ranolazine | Li et al. | 60.0 | 0.00 | 8.83 | 6.66 | 0 | 0 | 1 | 1 |
| Ranolazine | Ando et al. | 60.0 | 5.51 | 5.54 | 7.50 | 0 | 0 | 1 | 1 |
| Risperidone | Lanacster and Sobie | 60.0 | 0.31 | 0.22 | 124.31 | 1 | 1 | 1 | 1 |
| Risperidone | Kramer et al. | 60.0 | 2.84 | 0.98 | 195.80 | 1 | 1 | 0 | 0 |
| Risperidone | Mirams et al. | 60.0 | 0.31 | 0.22 | 124.31 | 0 | 1 | 1 | 1 |

| | | | | | | | | | |
|---|---|---|---|---|---|---|---|---|---|
| Risperidone | Ando et al. | 60.0 | 1.13 | 0.89 | 26.00 | 0 | 1 | 1 | 1 |
| Saquinavir | Crumb et al. | 60.0 | 66.84 | 21.21 | 12.50 | - | 1 | 0 | 0 |
| Saquinavir | Kramer et al. | 60.0 | 94.18 | 79.14 | 164.56 | 0 | 1 | 0 | 0 |
| Sertindole | Kramer et al. | 60.0 | 0.17 | 0.25 | 22.82 | 1 | 1 | 1 | 1 |
| Sertindole | Mirams et al. | 60.0 | 0.24 | 0.90 | 13.21 | 1 | 1 | 1 | 1 |
| Sertindole | Ando et al. | 60.0 | 0.78 | 0.71 | 30.94 | 0 | 1 | 1 | 1 |
| Sertindole | Crumb et al. | 60.0 | 0.00 | 0.00 | 11.41 | - | 1 | 1 | 1 |
| Sotalol | Li et al. | 60.0 | 3.61 | 1.25 | 12.50 | 1 | 1 | 1 | 1 |
| Sotalol | Crumb et al. | 60.0 | 2.77 | 1.08 | 9.23 | - | 1 | 1 | 1 |
| Sotalol | Ando et al. | 60.0 | 46.37 | 2.33 | 12.85 | 1 | 1 | 0 | 0 |
| Sotalol | Kramer et al. | 60.0 | 50.11 | 2.69 | 13.22 | 1 | 1 | 0 | 0 |
| Sotalol | Okada et al. | 60.0 | 0.00 | 0.00 | 36.09 | 1 | 1 | 1 | 1 |
| Sparfloxacin | Kramer et al. | 60.0 | 27.79 | 1.32 | 19.35 | 1 | 1 | 1 | 1 |
| Sparfloxacin | Ando et al. | 60.0 | 27.18 | 1.28 | 17.45 | 1 | 1 | 1 | 1 |
| Terfenadine | Li et al. | 60.0 | 12.81 | 0.93 | 11.30 | 0 | 1 | 1 | 1 |
| Terfenadine | Okada et al. | 60.0 | 2.16 | 34.55 | 15.45 | 1 | 1 | 1 | 1 |
| Terfenadine | Crumb et al. | 60.0 | 11.39 | 0.77 | 4.15 | - | 1 | 1 | 1 |
| Terfenadine | Kramer et al. | 60.0 | 0.92 | 0.23 | 7.68 | 1 | 1 | 1 | 1 |
| Terfenadine | Ando et al. | 60.0 | 7.46 | 3.61 | 7.89 | 1 | 1 | 1 | 1 |
| Terfenadine | Mirams et al. | 60.0 | 3.44 | 1.36 | 1.48 | 1 | 1 | 1 | 1 |
| Terodiline | Ando et al. | 60.0 | 16.88 | 11.64 | 4.06 | 1 | 1 | 1 | 1 |
| Terodiline | Kramer et al. | 60.0 | 16.55 | 7.57 | 6.67 | 1 | 1 | 1 | 1 |
| Thioridazine | Kramer et al. | 60.0 | 11.22 | 33.55 | 0.77 | 1 | 1 | 1 | 1 |
| Thioridazine | Mirams et al. | 60.0 | 3.67 | 2.63 | 0.05 | 1 | 1 | 1 | 1 |
| Thioridazine | Ando et al. | 60.0 | 17.65 | 34.88 | 16.67 | 1 | 1 | 1 | 1 |
| Verapamil | Kramer et al. | 60.0 | 63.25 | 0.28 | 4.48 | 0 | 0 | 0 | 0 |
| Verapamil | Crumb et al. | 60.0 | 80.22 | 0.00 | 16.03 | - | 0 | 0 | 0 |
| Verapamil | Ando et al. | 60.0 | 65.22 | 1.14 | 4.08 | 0 | 0 | 0 | 0 |
| Verapamil | Mirams et al. | 60.0 | 68.20 | 0.51 | 2.65 | 0 | 0 | 0 | 0 |
| Verapamil | Okada et al. | 60.0 | 46.74 | 9.40 | 3.38 | 0 | 0 | 0 | 0 |
| Verapamil | Li et al. | 60.0 | 69.88 | 0.00 | 5.33 | 0 | 0 | 0 | 0 |

**Table S12.** Common drugs: 46 drugs were common across more than one datasets. Here, we list the % block of $I_{CaV}$, $I_{Na,peak}$ for these drugs at drug concentrations equal to $IC_{60,hERG}$ to highlight the differences across the datasets. Moreover, we list the risk category each drug was assigned to in the original datasets ("Risk (O)" column) as well the risk category ("Risk") assigned here to analyze the merged dataset. "Prediction" column highlights whether a drug is classified as TdP+ (1) or TdP (0) for the merged dataset using the direct features. We also highlight whether a drug results in EAD or not at $C_{drug}$ = $IC_{60,hERG}$ in the "EAD" column. Drugs which ended up with different predictions are highlighted in gray.

78

## 2 METHODS AND RESULTS

### 2.1 Classification accuracy based on the derived Features taking into account the drug-induced block of all the available ion-channels for a particular drug

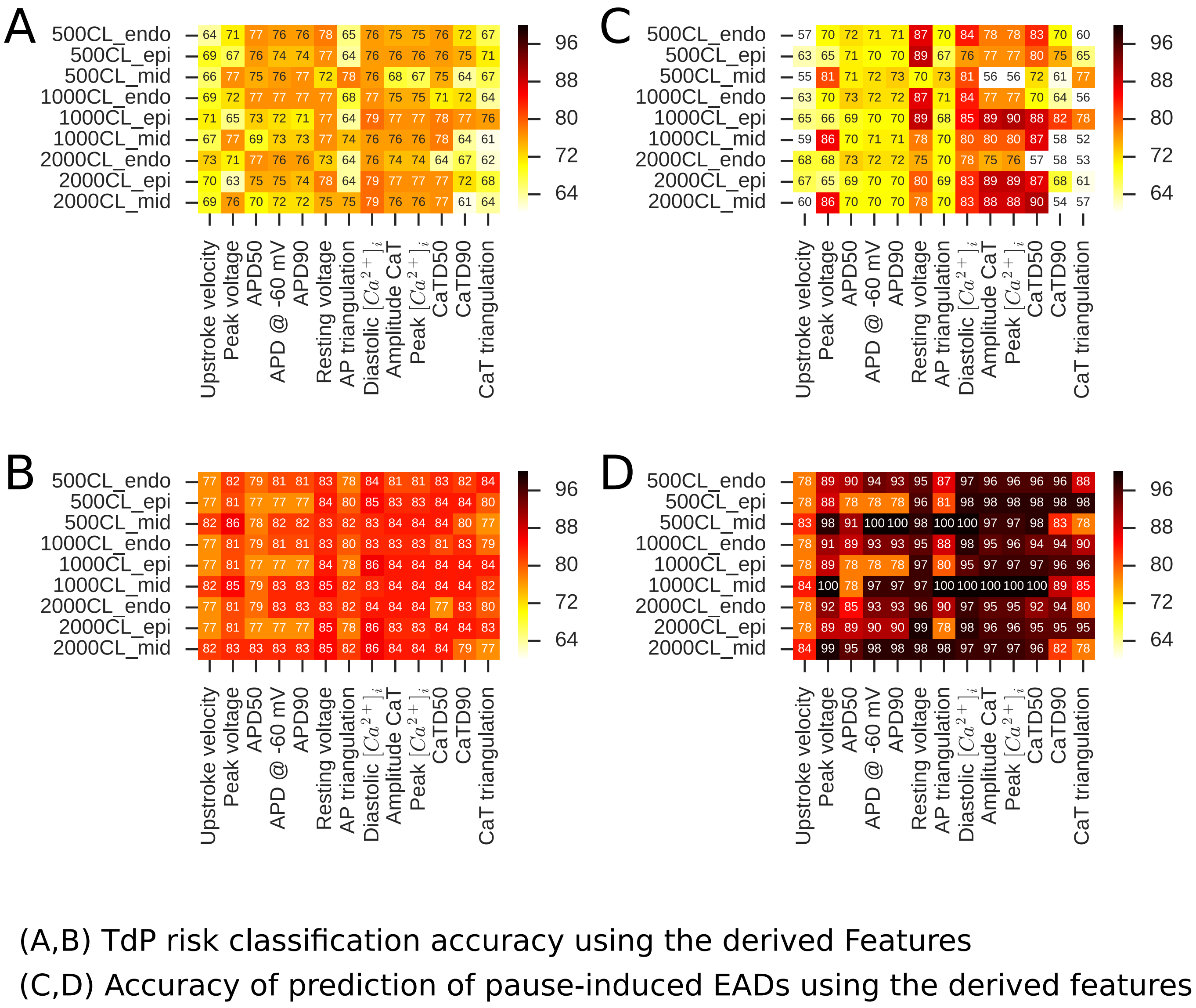


**Figure S1.** Heat maps of leave-one-out cross validation scores for logistic regression classifiers built on 13 features extracted from the APs and $Ca^{2+}$ transients in OHR model simulations. Drug-induced multi-channel block evaluated in the mid, endo and epi cell type at 500, 1000 and 2000ms pacing rates. TdP risk classification at (A) EFTPC drug concentrations and (B) drug concentrations equal to hERG $IC_{50}$. Classification by EAD induction at (C) EFTPC drug concentrations (D) drug concentrations equal to hERG $IC_{50}$.

| | $\beta_{intercept}$ | $\beta_{cav}$ | $\beta_{nal}$ | Figure/Table | $Coef_{nal}$ = $\beta_{nal}/\beta_{cav}$ | Threshold Y-axis = $\beta_{nal}/\beta_{intercept}$ |
|---|---|---|---|---|---|---|
| Dataset1 | 4.473618 | -0.195135 | - | **Figure 3** and **Table 4** | - | 22.925721 |
| Dataset2 | 5.571355 | -0.097846 | - | **Figure 3** and **Table 4** | - | 56.940049 |
| Dataset3 [cav] | 2.879890 | -0.146161 | - | **Figure 3** and **Table 4** | - | 19.703524 |
| Dataset3 [cav nal] | 22.949021 | -1.036844 | -1.64362 | **Figure 3** and **Table 4** | 1.58522 | 22.133535 |
| Dataset4 | 2.827687 | -0.063619 | - | **Figure 3** and **Table 4** | - | 44.447431 |
| Dataset5 [cav] | 2.300028 | -0.042746 | - | **Figure 4A** and **Table 4** | - | 53.806997 |
| Dataset5 [cav nal] | 2.771003 | -0.038716 | -0.0197162 | **Figure 4B** and **Table 4** | 0.509248 | 71.571871 |
| Dataset6 | 1.607803 | -0.037014 | - | **Figure 3** and **Table 4** | - | 43.437807 |
| Dataset7 [cav] | 2.548515 | -0.075452 | - | **Figure 3** and **Table 4** | - | 33.776838 |
| Dataset7 [cav nal] | 41.795781 | -0.640847 | -0.804278 | **Figure 3** and **Table 4** | 1.25502 | 65.219578 |
| Dataset8 | 1.471547 | -0.032359 | - | **Figure 3** and **Table 4** | - | 45.476341 |
| Dataset5 [cav] [target2] | 1.221071 | -0.047759 | - | **Figure 4C** | - | 25.567424 |
| Dataset3 [cav nal] [target2] | 2.188419 | -0.053652 | -0.0263147 | **Figure 4D** | 0.490471 | 40.789300 |

**Table S13.** Beta coefficients for Logistic Regression. Figure/Table column lists the corresponding figure and table number in the main article for which the regression coefficients and the thresholds are listed here.

## 2.2 Classification results using SVM and Neural Network

TdP classifiers from the direct features built using SVM and Neural networks yielded identical classification accuracy (85%) for the merged dataset as compared to logistic regression models. Classification accuracies obtained using SVM and Neural networks on derived features are shown in Figure S2 and resulted in maximum classification accuracy of 86%, as for the Logistic regression classifier. For the limited data with insignificant nonlinearities the choice of the classifier had not significant impact on the observed accuracies.

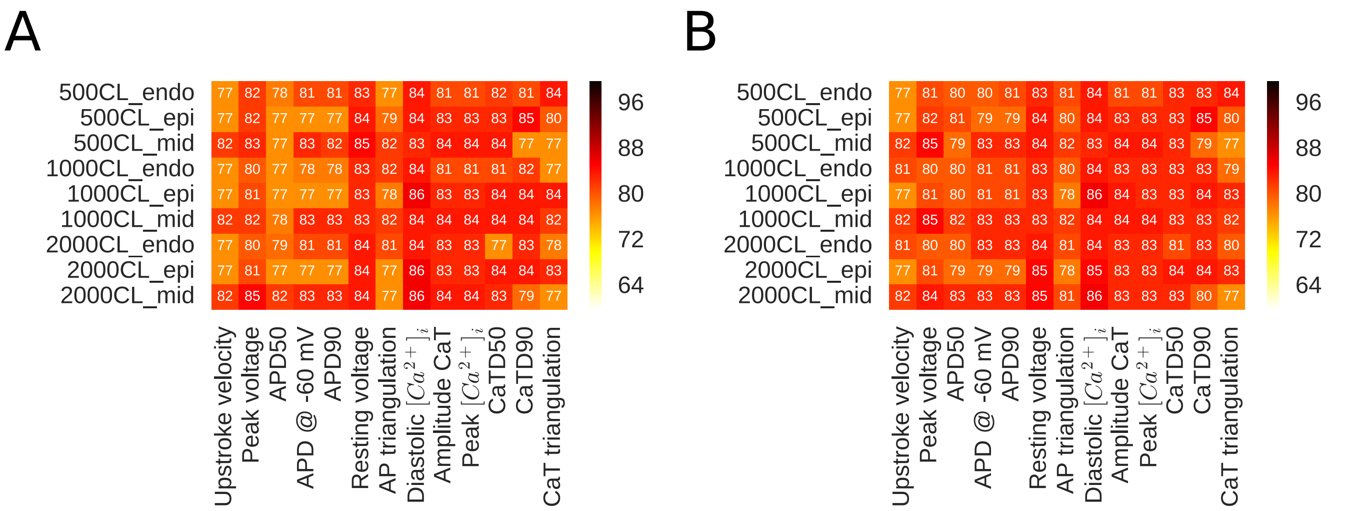


(A,B) TdP risk classification accuracy using the derived Features

**Figure S2.** Heat maps of leave-one-out cross validation scores for logistic regression classifiers from 13 features of the APs and $Ca^{2+}$ transients in OHR model simulations using 1) Support Vector machine 2) Feed forward neural network with 13 hidden units. Drug-induced multi-channel block evaluated in the mid, endo and epi cell type at 500, 1000 and 2000 ms pacing rates. TdP risk classification at drug concentrations equal to hERG $IC_{50}$.

### 2.3 GPU implementation

EAD simulations with fixed time step of 0.001 ms take approximately 2 mins per simulation (Intel Xeon E5-2660 v4, 2.00 GHz CPU). Hence, the 1000 simulations to generate the EAD space in Figure 2 (main article) result in approximately 2000 mins (33 hours) if executed serially. We parallelized the models using Python's PyCUDA package which allows access to NVIDIA's CUDA GPUs (8GB DDR5 graphics card with 2048 CUDA cores, Quadro M5000). The parallelization allowed execution of the 1000 simulations in approximately 8 hours providing a 4x improvement in the performance. However, the numerical methods and number of simulations will greatly affect these timings.

## REFERENCES

Ando, H., Yoshinaga, T., Yamamoto, W., Asakura, K., Uda, T., Taniguchi, T., et al. (2017). A new paradigm for drug-induced torsadogenic risk assessment using human iPS cell-derived cardiomyocytes. *Journal of Pharmacological and Toxicological Methods* 84, 111–127. doi:10.1016/j.vascn.2016.12.003

Champeroux, P., Viaud, K., El Amrani, A. I., Fowler, J. S. L., Martel, E., Le Guennec, J.-Y., et al. (2005). Prediction of the risk of Torsade de Pointes using the model of isolated canine Purkinje fibres. *British Journal of Pharmacology* 144, 376–385. doi:10.1038/sj.bjp.0706070

Colatsky, T., Fermini, B., Gintant, G., Pierson, J. B., Sager, P., Sekino, Y., et al. (2016). The Comprehensive in Vitro Proarrhythmia Assay (CiPA) initiative  Update on progress. *Journal of Pharmacological and Toxicological Methods* 81, 15–20. doi:10.1016/j.vascn.2016.06.002

Crumb, W. J., Vicente, J., Johannesen, L., and Strauss, D. G. (2016). An evaluation of 30 clinical drugs against the comprehensive in vitro proarrhythmia assay (CiPA) proposed ion channel panel. *Journal of Pharmacological and Toxicological Methods* 81, 251–262. doi:10.1016/j.vascn.2016.03.009

Fermini, B., Hancox, J. C., Abi-Gerges, N., Bridgland-Taylor, M., Chaudhary, K. W., Colatsky, T., et al. (2016). A New Perspective in the Field of Cardiac Safety Testing through the Comprehensive In Vitro Proarrhythmia Assay Paradigm. *Journal of Biomolecular Screening* 21, 1–11. doi:10.1177/1087057115594589

Kramer, J., Obejero-Paz, C. A., Myatt, G., Kuryshev, Y. A., Bruening-Wright, A., Verducci, J. S., et al. (2013). MICE Models: Superior to the HERG Model in Predicting Torsade de Pointes. *Scientific Reports* 3, 1–7. doi:10.1038/srep02100

Lancaster, M. C. and Sobie, E. A. (2016). Improved Prediction of Drug-Induced Torsades de Pointes Through Simulations of Dynamics and Machine Learning Algorithms. *Clinical Pharmacology and Therapeutics* 100, 371–379. doi:10.1002/cpt.367

Li, Z., Dutta, S., Sheng, J., Tran, P. N., Wu, W., Chang, K., et al. (2017). Improving the In Silico Assessment of Proarrhythmia Risk by Combining hERG (Human Ether-?-go-go-Related Gene) Channel?Drug Binding Kinetics and Multichannel Pharmacology. *Circulation: Arrhythmia and Electrophysiology* 10, e004628. doi:10.1161/CIRCEP.116.004628

Mirams, G. R., Cui, Y., Sher, A., Fink, M., Cooper, J., Heath, B. M., et al. (2011). Simulation of multiple ion channel block provides improved early prediction of compounds' clinical torsadogenic risk. *Cardiovascular Research* 91, 53–61. doi:10.1093/cvr/cvr044

Okada, J.-i. J., Yoshinaga, T., Kurokawa, J., Washio, T., Furukawa, T., Sawada, K., et al. (2015). Screening system for drug-induced arrhythmogenic risk combining a patch clamp and heart simulator. *Science Advances* 1, e1400142–e1400142. doi:10.1126/sciadv.1400142

Redfern, W., Carlsson, L., Davis, A., Lynch, W., Mackenzie, I., Palethrope, S., et al. (2003). Relationships between preclinical cardiac electrophysiology, clinical QT interval prolongation and torsade de pointes

for a broad range of drugs: evidence for a provisional safety margin in drug development. *Cardiovascular Research* 58, 32–45. doi:10.1016/S0008-6363(02)00846-5
